# Mechanistic basis of ligand efficacy in the calcium‐activated chloride channel TMEM16A

**DOI:** 10.15252/embj.2023115030

**Published:** 2023-11-20

**Authors:** Andy KM Lam, Raimund Dutzler

**Affiliations:** ^1^ Department of Biochemistry University of Zurich Zurich Switzerland

**Keywords:** chloride channel, cryo‐electron microscopy, efficacy, electrophysiology, ligand gating, Structural Biology

## Abstract

Agonist binding in ligand‐gated ion channels is coupled to structural rearrangements around the binding site, followed by the opening of the channel pore. In this process, agonist efficacy describes the equilibrium between open and closed conformations in a fully ligand‐bound state. Calcium‐activated chloride channels in the TMEM16 family are important sensors of intracellular calcium signals and are targets for pharmacological modulators, yet a mechanistic understanding of agonist efficacy has remained elusive. Using a combination of cryo‐electron microscopy, electrophysiology, and autocorrelation analysis, we now show that agonist efficacy in the ligand‐gated channel TMEM16A is dictated by the conformation of the pore‐lining helix α6 around the Ca^2+^‐binding site. The closure of the binding site, which involves the formation of a π‐helix below a hinge region in α6, appears to be coupled to the opening of the inner pore gate, thereby governing the channel's open probability and conductance. Our results provide a mechanism for agonist binding and efficacy and a structural basis for the design of potentiators and partial agonists in the TMEM16 family.

## Graphical abstract

The conformation of a pore‐lining helix in a key cellular calcium sensor coordinates calcium binding and chloride conduction.

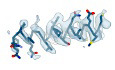

## Introduction

Agonist efficacy reflects the ability of the ligand to elicit a maximum response upon binding to its receptor. In ligand‐gated ion channels (LGICs), this is manifested in the maximum open probability of the channel when the binding sites are saturated. Efficacy is governed by the equilibrium between open and closed states and their relative stability when the agonist is bound. The long‐persisted view is that agonists work by shifting the equilibrium toward the open state for which they display a higher affinity, with more efficacious agonists necessarily being more selective for the open state (Colquhoun, 1998). Recent studies on nicotinic and glycine receptors have, however, shown that, instead of a concerted transition, channel activation involves an intermediate conformation that represents an activated, pre‐open state where rearrangements at the ligand‐binding site have likely occurred (Lape *et al*, 2008; Mukhtasimova *et al*, 2009; Jadey & Auerbach, 2012). Once this state is reached, the channel pore is able to open with similar efficiency even when bound to agonists with different efficacy that might elicit distinct conformations at the occupied binding site (Lape *et al*, 2008; Yu *et al*, 2021).

A similar mechanism underlying channel activation also applies to the ion channel TMEM16A, which opens in response to an increase in the intracellular Ca^2+^ concentration (Caputo *et al*, 2008; Schroeder *et al*, 2008; Yang *et al*, 2008). TMEM16A mediates important physiological processes such as epithelial chloride transport and smooth muscle contraction and has been proposed to be a therapeutic target for diseases including asthma, hypertension, stroke, and cystic fibrosis (Huang *et al*, 2012; Danahay *et al*, 2020; Al‐Hosni *et al*, 2022; Galietta, 2022). The protein is a homodimer, with each subunit containing an ion conduction pore and a principal Ca^2+^‐binding site that are closely apposed (Dang *et al*, 2017; Paulino *et al*, 2017a, 2017b). Both pores act independently and are activated by the binding of two Ca^2+^ ions to the principal site (Jeng *et al*, 2016; Lim *et al*, 2016). Additional regulation is conferred by a proposed allosteric Ca^2+^‐binding site located remote from the ion conduction path and the lipid PIP_2_ (Ta *et al*, 2017; Arreola & Hartzell, 2019; Le *et al*, 2019; Tembo *et al*, 2019; Yu *et al*, 2019; Le & Yang, 2020; Jia & Chen, 2021). In the absence of Ca^2+^, the vacant principal binding site acts as an electrostatic gate to impede anion conduction, but is neutralized by Ca^2+^ binding to enable the channel to conduct with higher capacity (Lam & Dutzler, 2018). An equivalent process was proposed to account for ion selectivity changes in the scramblase TMEM16F, which also shows features of an ion channel (Ye *et al*, 2019). Besides the reversal of pore electrostatics, Ca^2+^ binding triggers a conformational change of the pore‐lining helix α6, which rearranges to coordinate the bound Ca^2+^, leading to the closure of the binding site (Paulino *et al*, 2017a). This movement is then propagated to the channel pore to release a hydrophobic gate at the inner pore entrance and to enable structural rearrangements in the outer vestibule and narrow neck region of the hourglass‐shaped pore, which together open up a pathway to allow anion conduction (Lam *et al*, 2021, 2022; Lam & Dutzler, 2021). The central role of α6 in channel activation has also been observed by others (Peters *et al*, 2018).

Channel gating in TMEM16A is a multi‐state process that involves one or more intermediates prior to opening (Lam & Dutzler, 2021), akin to pentameric LGICs such as nicotinic and glycine receptors, where a pre‐open intermediate, referred to as flipped or primed, has been observed (Lape *et al*, 2008; Mukhtasimova *et al*, 2009; Gupta *et al*, 2017). In TMEM16A, the initial transition leads to the first intermediate that displays a higher affinity for Ca^2+^ where the channel is in an activated yet still nonconducting state, as presumably represented by a Ca^2+^‐bound conformation (PDBID 5OYB) where α6 has rearranged and the binding site is closed while the pore has not yet opened. A second transition results in a pre‐open state that is more accessible when two Ca^2+^ are bound and is plausibly a mechanistic counterpart of a conformation where the outer pore has expanded. This state is characterized by a movement of α3 and α4 where these pore‐lining helices adopt an upward and outward conformation and can be stabilized by channel blockers that access from the extracellular side (PDBID 7ZK3) (Lam *et al*, 2022). These structural changes are reminiscent of an outer‐pore gate that was proposed to open upon activation, which provides access to extracellular pore blockers (Dinsdale *et al*, 2021). It is notable that the “up” and “down” conformations of the α3/4 pair are both sampled in the Ca^2+^‐bound closed state (Paulino *et al*, 2017a; Lam *et al*, 2022), consistent with their relevance in channel gating. In this pre‐open state, the channel is presumably primed to open, a motion that is not structurally well understood but corresponds to the release of a hydrophobic gate located at the inner entrance of the pore near the principal Ca^2+^‐binding site (Lam *et al*, 2021). Once fully bound by Ca^2+^, channel opening is efficacious, indicating that the open state is energetically the most favorable amongst all accessible states (Lam & Dutzler, 2021).

Here, we studied the mechanisms underlying the efficacy of channel opening in TMEM16A by combining kinetic analysis and structural investigations. We show that ligand efficacy is dictated by the conformation of the pore‐lining helix α6 and that the closure of the binding site, which involves the formation of a π‐helix below a hinge region in α6, is coupled to the opening of the pore by weakening the interactions within the inner pore gate. Our results reveal the conformational coupling between different functional modules along the channel's activation pathway and identify α6 as a potential site for pharmacological intervention. Collectively, they provide a structural and mechanistic basis for the design of potentiators and partial agonists in the TMEM16 family.

## Results

### Agonist‐coupled rearrangements at the binding site, efficacy, and cooperativity

**Figure 1:**
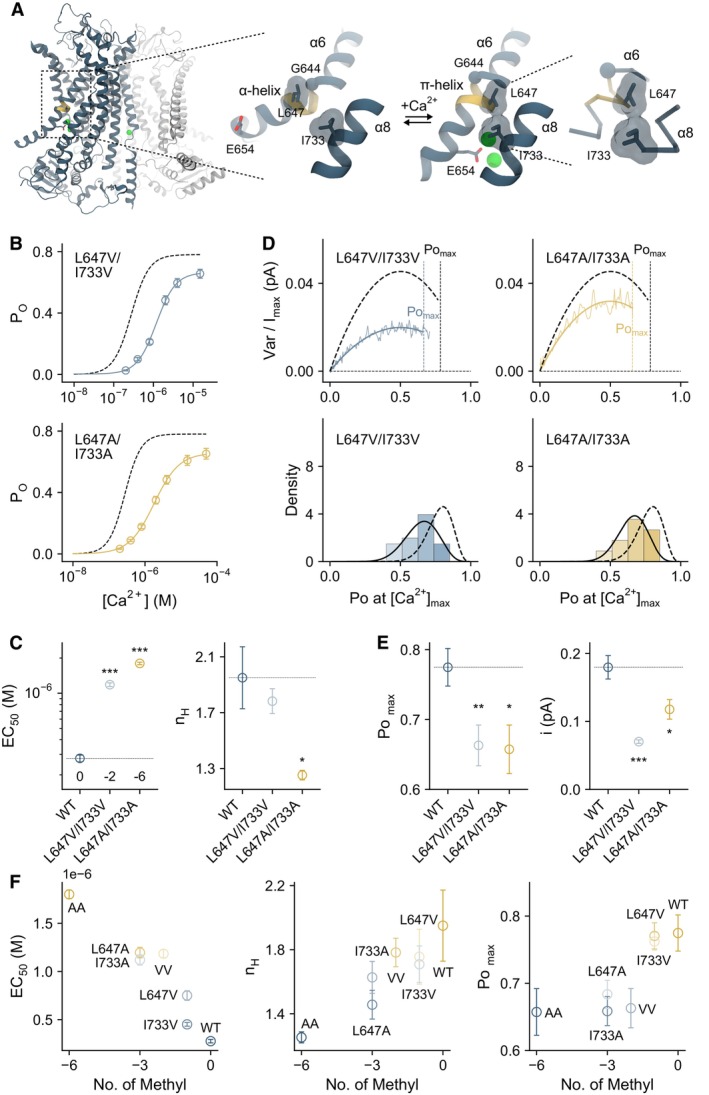
Functional characterization of binding site rearrangement Conformational rearrangement of α6 associated with Ca^2+^ binding. Left, structure of TMEM16A in a Ca^2+^‐bound state (Lam *et al*, 2022) viewed from within the membrane. The two subunits are displayed in unique colors. Middle, rearrangement of α6 upon Ca^2+^ binding. Selected helices are shown as ribbon, sidechains as sticks, the Cα of Gly 644 as sphere, and bound Ca^2+^ as green spheres. Right, close‐up view of the π‐helical region in Cα representation. The volume of the sidechains of Leu 647 and Ile 733 is shown in a surface representation. The region undergoing the α‐to‐π‐helix transition is shown in yellow. PDBID 5OYG (Ca^2+^‐free) and 7ZK3 (Ca^2+^‐ and 1PBC‐bound) are displayed.Concentration‐Po relations for the indicated mutants at +80 mV. Data are averages of 8 and 5 patches for L647V/I733V and L647A/I733A respectively, and errors are SEM. Solid line is a fit to the Hill equation. Dashed line is the relation of wild‐type.EC_50_ and n_H_ of the indicated constructs. Shown are the averages of the data displayed in (B) and errors are SEM. The number of methyl groups truncated relative to wild‐type is indicated in the left panel. *n* = 8, 8, and 5 for WT, L647V/I733V, and L647A/I733A respectively. *t*‐test: ***P < 0.005.Top, merged and averaged variance‐current relations at a saturating Ca^2+^ concentration at +80 mV. Data are averages of 20 and 10 patches for L647V/I733V and L647A/I733A recorded at 15 μM and 50 μM Ca^2+^ respectively. Solid line is a fit to Equation 5 in Supplementary Methods. Dashed line is the relation of wild‐type. Dotted lines indicate the respective maximum Po's. Bottom, histograms of the maximum Po obtained from individual measurements. Solid line is a fit to the beta distribution. Dashed line is the distribution of wild‐type.Po_max_ and i of the indicated constructs. Shown are the averages of the data displayed in (D), and errors are SEM. *n* = 11, 20, and 10 for WT, L647V/I733V, and L647A/I733A respectively. *t*‐test: *P < 0.05; **P < 0.01; ***P < 0.005.EC_50_, n_H_, and maximum Po as a function of hydrophobic volume. For EC_50_ and n_H_, data are best‐fit values, and errors are standard errors. For maximum Po, data are averages, and errors are SEM. Each data point corresponds to the properties of a mutant in the L647/I733 series shown in this figure, Fig EV1, and Appendix Tables S1 and S2. WT, *n* = 8; L647V, *n* = 7; I733V, *n* = 8; L647V/I733V, *n* = 8; L647A, *n* = 8; I733A, *n* = 8; L647A/I733A, *n* = 5. VV, L647V/I733V; AA, L647A/I733A. Source data are available online for this figure.

**Figure EV1:**
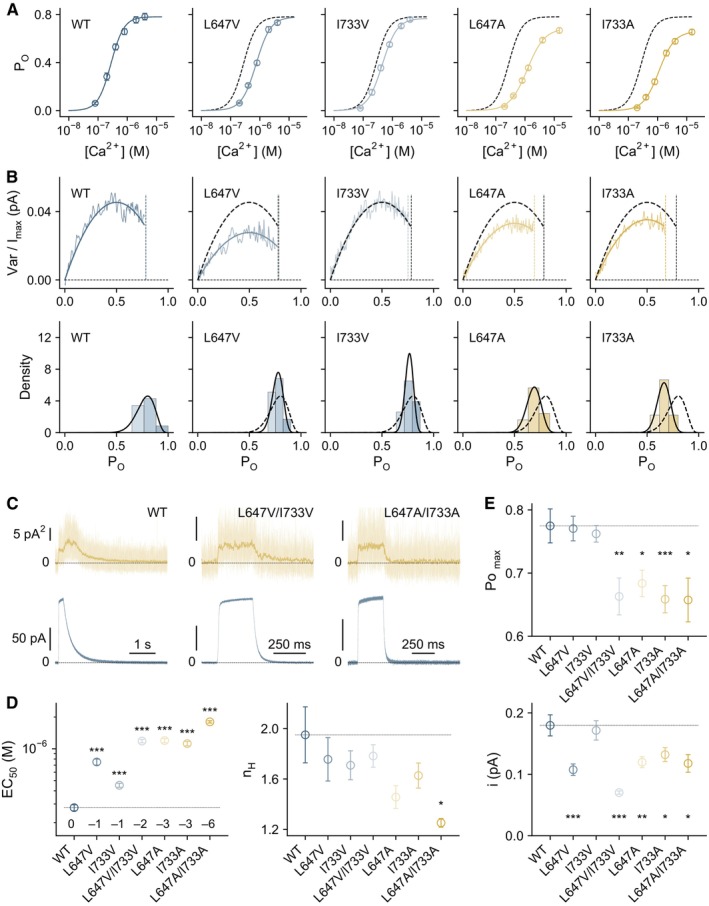
Activation properties of mutants Concentration‐Po relations for the indicated mutants at +80 mV. Data are averages of 8, 7, 8, 8, and 8 patches for WT, L647V, I733V, L647A, and I733A respectively, and errors are SEM. Solid line is a fit to the Hill equation. Dashed line is the relation of wild‐type.Top, merged and averaged variance‐current relations at a saturating Ca^2+^ concentration at +80 mV. Data are averages of 11, 8, 10, 12, and 10 patches for WT, L647V, I733V, L647A, and I733A respectively. Solid line is a fit to Equation 5 in Supplementary Methods. Dashed line is the relation of wild‐type. Dotted lines indicate the maximum Po. Bottom, histograms of the maximum Po obtained from individual measurements. Solid line is a fit to the beta distribution. Dashed line is the distribution of wild‐type.Representative mean current and variance upon a step‐exchange from zero to saturating Ca^2+^ and back. The raw variance is overlaid with its Gaussian moving average. Dashed lines indicate the zero current/variance levels.EC_50_ and n_H_ of the indicated constructs. Data are averages of the indicated number of patches shown in Appendix Table S1, and errors are SEM. The number of methyl groups truncated relative to wild‐type is indicated in the left panel. WT, *n* = 8; L647V, *n* = 7; I733V, *n* = 8; L647V/I733V, *n* = 8; L647A, *n* = 8; I733A, *n* = 8; L647A/I733A, *n* = 5. *t*‐test: *P < 0.05; ***P < 0.005.Po_max_ and i of the indicated constructs. Data are averages of the indicated number of patches shown in Appendix Table S2, and errors are SEM. WT, *n* = 11; L647V, *n* = 8; I733V, *n* = 10; L647V/I733V, *n* = 20; L647A, *n* = 12; I733A, *n* = 10; L647A/I733A, *n* = 10. *t*‐test: *P < 0.05; **P < 0.01; ***P < 0.005.

Activation of LGICs is initiated by conformational changes around the ligand‐binding site in response to agonist binding (Plested, 2016). In TMEM16A, this is manifested in a structural rearrangement of the pore‐lining helix α6, which harbors residues that coordinate the bound agonist Ca^2+^ (Paulino *et al*, 2017a). Accompanying this hinge‐like movement around a conserved glycine residue, this region transitions from an α‐helical configuration to a π‐helix, bringing an essential glutamate, Glu 654, into direct contact with the bound Ca^2+^ (Fig 1A). In addition to this ionic interaction, our previous study has identified several residues around the Ca^2+^‐binding site as important determinants of channel activation, which is manifested by a profound decrease in the potency of Ca^2+^ upon mutation to alanine (Lam *et al*, 2021). Here, we focus on Leu 647 on α6 and Ile 733 on α8, both within the π‐helical region, which are brought into van der Waals contact in the Ca^2+^‐bound state upon α6 activation (Figs 1 and EV1). Decreasing the sidechain volume of these residues by mutation to valine or alanine severely impairs channel activation, as indicated by a pronounced right‐shift in the potency of Ca^2+^ (Fig 1B and C, Appendix Table S1). The same mutations also reduce cooperativity as illustrated in the lowered Hill coefficient (n_H_), which in the double mutant L647A/I733A approaches one (i.e., 1.3, Fig 1B and C, Appendix Table S1). While the potency is influenced by both Ca^2+^ binding and channel gating, nonstationary noise analysis indicates that this impairment in activation is partly due to a reduction in the channel's maximum open probability (Po_max_) measured at a saturating ligand concentration, suggesting that Ca^2+^ has become less efficacious in these mutants (Figs 1D and E, and EV1C and E, Appendix Table S2). The reduction in Po_max_, is accompanied by a decrease in the unitary current, as reflected in the initial slope of the noise parabola when Leu 647 and Ile 733 are mutated (Figs 1D and E, and EV1C and E, Appendix Table S2), which indicates a simultaneous perturbation of the anion conduction pathway. By analyzing different substituted aliphatic sidechains, we further observed that the severity of the gating defects correlates with the hydrophobic volume mediating this interaction, as exemplified in the trends of the potency (EC_50_), cooperativity (n_H_), and Po_max_ changes (Figs 1F and EV1D and E). Despite the general reduction in their cooperativity of activation, most of these mutants likely retain the ability to bind two Ca^2+^, as reflected in their Hill coefficients, which remain consistently above one (Fig 1F, middle, and Fig EV1D, right). Together, these results suggest a hydrophobic interaction within the π‐helical region close to the Ca^2+^‐binding site, which is mediated by Leu 647 on α6 and Ile 733 on α8, to be crucial in stabilizing the agonist‐coupled rearrangement of α6. The impairment of this interaction has evidently structural consequences that impact on the efficacy of pore opening, the agonist binding process, and channel conductance in TMEM16A.

### Agonist‐coupled rearrangements stabilize the open state

**Figure 2:**
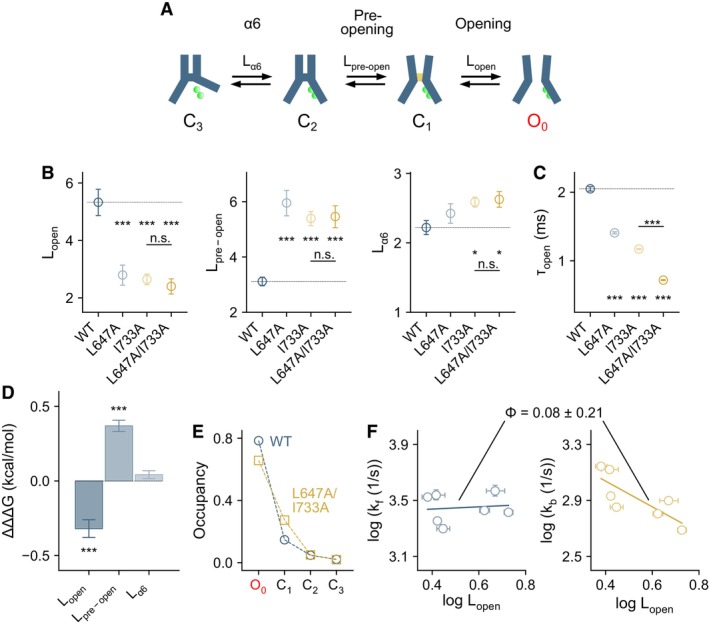
Binding site closure, efficacy, and gating mechanism Mechanism depicting gating transitions at saturating Ca^2+^ concentrations. The minimum number of states that can account for the gating properties of the wild‐type channel is shown (Lam & Dutzler, 2021). This scheme comprises three closed states, interpreted as α6‐resting (C_3_), α6‐activated (C_2_), and nonconductive outer‐pore‐open (C_1_), and one open state in which the inner pore gate is opened (O_0_). A sequential model is likely approximative, but is reasonable given that channel opening is minimal in the α6‐resting (C_3_) state. Additional states/transitions might exist, although they likely occur with a much lower probability and are therefore difficult to detect and characterize functionally.Forward equilibrium constants for the indicated transitions estimated from autocorrelation analysis (Equations 7–9 in Supplementary Methods, see Appendix Fig S1). Data are best‐fit values of the averaged spectra from 7, 5, 6, and 7 patches for WT, L647A, I733A, and L647A/I733A respectively, and errors are 95% confidence intervals. Dashed line indicates the value of wild‐type. *t*‐test: n.s., nonsignificant; *P < 0.05; ***P < 0.005.Mean open time calculated from the estimated rate constant $k_{01}$. Data were calculated from the best‐fit values, and errors are 95% confidence intervals. The number of replicates is as in (B). *t*‐test: ***P < 0.005.Coupling energies (ΔΔΔG) for the indicated transitions. Bars indicate quantities calculated using Equation 10 in Supplementary Methods, and errors are standard errors. The number of replicates is as in (B). *t*‐test: ***P < 0.005.Equilibrium occupancy of states calculated, respectively, with the best‐fit values of wild‐type and L647A/I733A using Equation 7 in Supplementary Methods.Rate‐equilibrium relations for the opening transition. Data are best‐fit values, and errors are 95% confidence intervals. Error bars are not visible when they are smaller than the symbols. k_f_ and k_b_ are the forward and backward rate constants respectively. Solid lines are a simultaneous fit to a pair of rate‐equilibrium relations (Equation 11 in Supplementary Methods), yielding a single phi value (best‐fit ± standard error) for the forward and backward transitions. Each data point corresponds to the properties of a mutant in the L647/I733 series shown in Appendix Fig S1 and Appendix Table S3. Source data are available online for this figure.

As shown previously, channel gating in TMEM16A is a multi‐state process that starts with a conformational change at the Ca^2+^‐binding site, followed by a transition into a pre‐open state that is characterized by a widening of the outer pore, and finally the opening of the channel upon the release of a hydrophobic gate at the inner pore entrance (Lam & Dutzler, 2021) (Fig 2A). To understand how gating is affected when the agonist‐coupled rearrangements are disrupted, we analyzed the transitions amongst these states in the L647/I733 series of mutants via autocorrelation analysis that we described previously (Lam & Dutzler, 2021) (Fig 2, Appendix Fig S1). In combination with nonstationary noise analysis, this approach allows the determination of the rate and equilibrium constants from steady‐state macroscopic currents for a given mechanism, and thus the characterization of transition probabilities between states and their lifetimes (Lam & Dutzler, 2021). For the described mechanism, the estimated parameters likely represent a unique solution as a single, well‐defined minimum of the sum‐of‐squares error, calculated using the experimental data, exists for each of the directly fitted rate constants within the observed time scales (Appendix Fig S2). Consistent with these properties, the confidence intervals of these parameters are typically within 10% of the fitted values (Appendix Table S3).

We found that disrupting the interaction between Leu 647 and Ile 733 energetically destabilizes the open state as the forward equilibrium constant (L_open_) is considerably lowered in the L647/I733 series of mutants (Fig 2B, Appendix Table S3). An opposite effect was observed for the pre‐opening step (described by L_pre‐open_), where perturbing the L647/I733 interaction promotes the transition into this intermediate state (Fig 2B, Appendix Table S3). Unexpectedly, the equilibrium of the initial agonist‐coupled transition (described by L_α6_) is only minimally affected (Fig 2B, Appendix Table S3), which might point toward a negligible role of both residues in this step, or alternatively a structurally distinct but energetically similar rearrangement at the Ca^2+^‐binding site. The latter could be due to a compensatory effect where the absence of the energetically costly π‐helix conformation (see later) might be offset by an inadequate coordination of the bound Ca^2+^ in the mutants. The decreased stability of channel opening is primarily attributable to the shortening of the open‐state lifetime (τ_open_) (Fig 2C), resulting in a higher tendency to transition into closed states. By analyzing single and double mutants in a double‐mutant cycle, our data further confirm a functional interaction between Leu 647 and Ile 733 in stabilizing the agonist‐coupled rearrangement of α6 as the individual mutations L647A and I733A perturb the opening and pre‐opening transitions almost as much as their combination (Fig 2B). The polarity of the resulting coupling energies reflects the contribution of the L647/I733 interaction in stabilizing the open state while limiting the pre‐opening transition (Fig 2D). The calculated equilibrium occupancies, using the estimated parameters, suggest that the mutant L647A/I733A is more likely to reside in the pre‐open state in expense of the open state (Fig 2E).

To gain insights into the timing of the affected rearrangements during the opening step, we analyzed the relationship between the rate and equilibrium constants of this transition in the L647/I733 series of mutants (Fig 2F). The relative timing of the perturbed motion can be characterized by its phi value (ranging between 0 and 1), which is reflected in the slope of the rate‐equilibrium relation (Leffler, 1953; Grosman *et al*, 2000; Sorum *et al*, 2015). A phi value of close to zero corresponds to a scenario where the mutations affect primarily the final state, indicative of a rearrangement that occurs relatively late on the reaction coordinate, whereas a value close to one suggests a rearrangement that occurs early on the reaction coordinate. In the L647/I733 series of mutants, the closing rate (the backward rate constant k_b_) is affected almost as much as the forward equilibrium constant (Fig 2F), indicating a rather selective perturbation of the open state. The experimental phi value of 0.08 for L_open_ suggests that the L647/I733 interaction and α6 rearrangement facilitate a structural change that occurs relatively late in the opening transition (Fig 2F). Notably, a direct disruption of the gate results in a phi value of zero for the opening transition (L_open_) as observed in our previous study (Lam & Dutzler, 2021), showing a close correspondence with the motion facilitated by the L647/I733 interaction during this same transition. This further emphasizes the role of these two residues in allosterically stabilizing the open state.

### Agonist‐coupled rearrangements stabilize an open‐pore geometry

**Figure 3:**
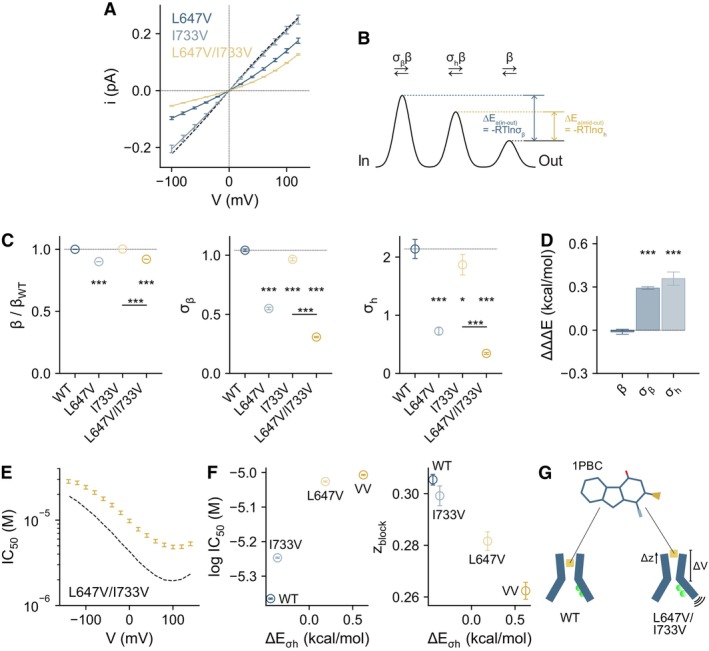
Binding site closure, ion conduction, and pore conformation Instantaneous current–voltage (I‐V) relations of the indicated mutants at a saturating Ca^2+^ concentration on a unitary scale. Data were scaled according to the estimated unitary current from nonstationary noise analysis at +80 mV (Figs 1 and EV1, Appendix Table S2). Data are averages of 6, 8, 8, and 6 patches for WT, L647V, I733V, and L647V/I733V respectively, and errors are SEM. Solid lines are fits to a model of ion permeation shown in (B) (Equation 1 in Supplementary Methods). Dashed line is the relation of wild‐type.Energy profile of a minimal ion permeation model to account for the I‐V relations (Paulino *et al*, 2017b).Conduction parameters for the indicated barriers. Data are best‐fit values, and errors are 95% confidence intervals. Dashed line indicates the value of wild‐type. The number of replicates is as in (A). *t*‐test: *P < 0.05; ***P < 0.005.Coupling energies (ΔΔΔE) for the indicated barriers. Bars indicate quantities calculated using Equations 2, 3, and 10 in Supplementary Methods, and errors are standard errors. The number of replicates is as in (A). *t*‐test: ***P < 0.005.Inhibition by the pore blocker 1PBC as a function of voltage in L647V/I733V. Data are best‐fit values from a fit to the Hill equation using averaged concentration‐response curves from 7 patches at the indicated voltages, and errors are 95% confidence intervals. Dashed line is the relation of wild‐type.Correlation between the blocking properties of 1PBC and the magnitude of the energy barrier in the narrow neck region of the pore (ΔE_σh_, calculated from the data displayed in (C)). The IC_50_ values at 0 mV are plotted. z_block_ is equivalent to the fraction of the transmembrane electric field operating on the blocker at its binding site. Data are the best‐fit values from a fit to Equation 4 in Supplementary Methods in the exponential region (±40 mV) of the relations shown in (E) and Appendix Fig S3E and errors are standard errors determined from 6, 6, 6, and 7 patches for WT, L647V, I733V, and L647V/I733V respectively. The number of replicates for ΔE_σh_ is as in (A).Schematic summarizing the data. Disrupting binding site closure in the mutant L647V/I733V leads to a partial collapse of the pore and structural perturbations at the 1PBC‐binding pocket. The narrowing of the pore is accompanied by a shift of the blocker binding site towards the extracellular side. Source data are available online for this figure.

Nonstationary noise analysis of the investigated mutants indicates that, in addition to a decrease in the Po_max_, the conductance of the channel is considerably lower when the agonist‐coupled rearrangements are disrupted (Figs 1D and E, and EV1B and E, Appendix Table S2). To understand how the properties of the pore are affected, we analyzed the current–voltage (I‐V) relations and extracted the energetic effects of the L647/I733 series of mutations on ion conduction (Fig 3A, Appendix Table S4). Changes in the energetics of anion diffusion and the nature of the resulting current rectification depend on the position of the perturbations with respect to the ion conduction path, allowing the localization of the underlying structural changes (Paulino *et al*, 2017b). Energies were obtained from a fit to a three‐barrier model that was introduced previously to approximate the rate‐limiting steps for the diffusion of an ion across the narrow region of the hourglass‐shaped pore. This analysis yields kinetic parameters (σ_β_ and σ_h_) that reflect the energy difference of the inner and central barrier, related to the entry of the anion from the inside and its passage through the neck region, relative to the outer barrier that describes the release of the anion to the extracellular part of the pore (Fig 3B and C). When the I‐V plots are placed on a unitary scale, this analysis further allows the estimation of changes in the outer barrier (β/β_WT_), and thus a more complete description of the ion conduction path (Fig 3C).

We found that disrupting the L647/I733 interaction lowers the overall conductance of the channel (Figs 3A and EV1E, Appendix Fig S3A, Appendix Table S2), owing to a considerable elevation of energy barriers at the intracellular entrance of the neck (σ_β_) and inside the narrow pore (σ_h_), whereas the outer barrier (β/β_WT_) is largely unaffected for sidechain replacements with valine and is somewhat decreased in the case of alanine mutations (Fig 3C, Appendix Fig S3B). This is manifested in the pronounced outward rectification of the mutants L647V and L647V/I733V, indicating a more constricted geometry both at the inner pore and the narrow neck region (Fig 3A and C). We analyzed these energetic effects in a double‐mutant cycle and observed a functional interaction between Leu 647 and Ile 733 in stabilizing a more dilated pore geometry, as reflected in the nonadditive effects of these mutations (Fig 3D, Appendix Fig S3C). These results suggest an allosteric process that relays the conformation of the binding site to the widening of the ion conduction path in the open state, as both residues are located away from the pore.

A change in the pore geometry in the narrow neck region in this set of mutants is further supported by the binding properties of the blocker 1PBC (Peters *et al*, 2015) in the open pore. We previously showed that 1PBC binds at the extracellular opening of the narrow neck region, and that the binding of 1PBC is in part governed by steric complementarity (Lam *et al*, 2022). Consistent with a coordinated conformational change of the pore helices, impaired α6 rearrangement is associated with putative structural changes at the outer entrance of the narrow neck, as suggested by the lower affinity of 1PBC in L647V/I733V and related mutants, with varying degrees of severity (Fig 3E and F, Appendix Fig S3D–F). This is accompanied by a decrease in the voltage sensitivity and the valence of block, z_block_, which reflects the location of the binding site perpendicular to the membrane plane, indicating that 1PBC penetrates less into the channel pore from the extracellular side when α6 rearrangement is disrupted (Fig 3E and F, Appendix Fig S3D–F). In this group of mutants, the lowering of z_block_ correlates strongly with the elevation of the energy barrier inside the narrow neck, confirming a partial collapse of the pore and a steric origin of the elevated barriers for ion conduction (Fig 3F). Together, these results show that the conformational state of α6 does not only dictate the channel's opening efficacy but also the degree of pore opening (Fig 3G).

### α6 conformation dictates efficacy

**Figure 4:**
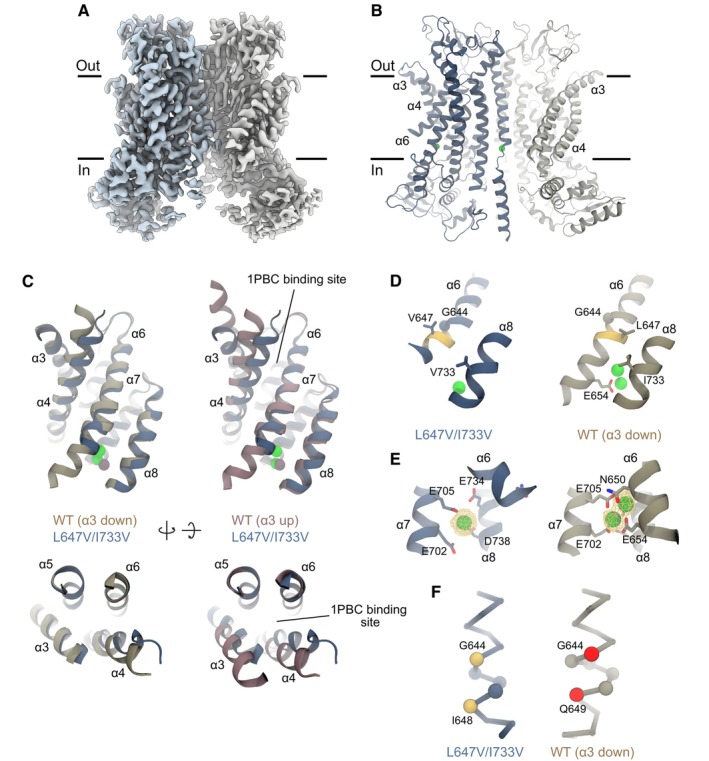
Structural basis for partial efficacy A, BCryo‐EM map (A) and ribbon representation (B) of L647V/I733V at a saturating Ca^2+^ concentration in the detergent GDN viewed from within the membrane. Black lines indicate membrane boundaries.CSuperposition of the pore region of the L647V/I733V with the wild‐type Ca^2+^‐bound (PDBID 5OYB, “α3 down” conformation rebuilt in Lam *et al*, 2022) and the wild‐type Ca^2+^/1PBC‐bound (PDBID 7ZK3, “α3 up” conformation where the outer pore is open; Lam *et al*, 2022) structures viewed from within the membrane (Top) and from the extracellular side (Bottom). Bound Ca^2+^ are shown as purple and green spheres in the L647V/I733V and wild‐type models respectively.D, Eα6 conformations in the Ca^2+^‐bound state (D) and Ca^2+^‐binding site (E). Selected helices are shown as ribbon, sidechains as sticks, the Cα of Gly 644 as sphere, and bound Ca^2+^ as green spheres. The region undergoing an α‐to‐π‐helix transition in wild‐type is highlighted in yellow. The densities for the bound Ca^2+^ ions are shown in (E).FSection of α6 around Gly 644 in Cα representation. Yellow and red spheres depict pairs of hydrogen‐bonded positions in α‐helix and π‐helix conformations respectively. The Cα in between are shown as spheres.

**Figure EV2:**
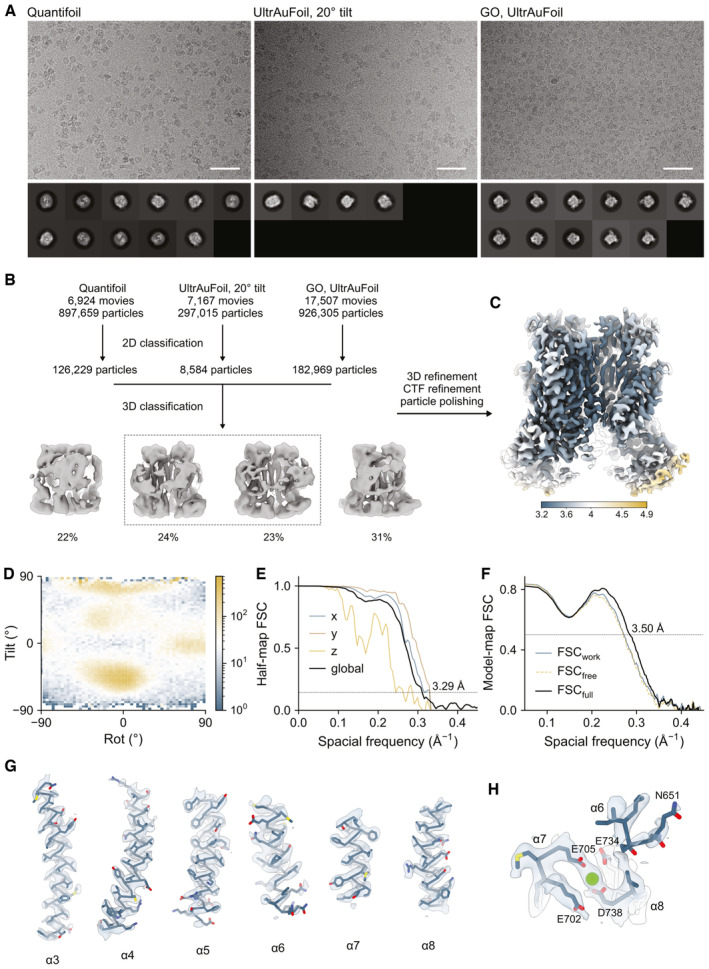
Cryo‐EM reconstruction of Ca^2+^‐bound L647V/I733V ARepresentative micrographs (scale bar: 50 nm) and 2D class averages of L647V/I733V in the presence of Ca^2+^ for the indicated samples.BData processing workflow.CLocal resolution of the final map estimated using RELION.DAngular distribution of particle projections used in the final refinement. Scale bar indicates the number of particle images.EHalf‐map FSCs.FModel‐map FSCs.G, HSections of cryo‐EM densities of (G) selected transmembrane helices and (H) the principal Ca^2+^‐binding site superimposed on the refined model.

To understand the rearrangements underlying the efficacy of pore opening, we determined a cryo‐EM structure of L647V/I733V, whose gating properties are similarly affected compared to the less conservative mutant L647A/I733A but which is characterized by a more severely perturbed ion conduction pathway (Figs 1B–E and 3A, Appendix Fig S3A), in the presence of a saturating Ca^2+^ concentration (Figs 4A and B, and Fig EV2). The reconstruction was obtained by combining datasets collected from samples prepared on cryo‐EM grids with distinct chemical properties, each showing a distinct preferred orientation of particles and at different tilts. Together, the pooled datasets yielded complementary particle views for the final map which, despite the remaining anisotropy, was much improved and provided a largely undistorted view of the protein (Table 1, Fig EV2).

**Table 1:** Cryo‐EM data collection, processing, refinement, and validation statistics.

	Quantifoil	UltrAuFoil, 20°	GO, UltrAuFoil
Data collection and processing			
Microscope	FEI Titan Krios G3i	FEI Titan Krios G3i	FEI Titan Krios G3i
Camera	Gatan K3 GIF	Gatan K3 GIF	Gatan K3 GIF
Imaging mode	Super‐resolution counted	Super‐resolution counted	Super‐resolution counted
Magnification	130,000	130,000	130,000
Voltage (kV)	300	300	300
Energy filter slit width (eV)	20	20	20
Electron dose (e^−^/Å^2^)	66.1	66.1	62.5
Defocus range (μm)	−2.4 to −1.0	−2.4 to −1.0	−2.4 to −1.0
Pixel size (Å)^a^	0.659 (0.3295)	0.659 (0.3295)	0.659 (0.3295)
Initial particle images (no.)	897,659	297,015	926,305
Final particle images (no.)	103,964	6,242	34,728
Symmetry imposed	C2		
Map resolution (Å) FSC threshold 0.143	3.29		
Map resolution range (Å)	3.2–4.9		
Refinement			
Initial model	PDBID 7B5D		
Model resolution (Å) FSC threshold 0.5	3.50		
Map sharpening B factor (Å^2^)	−91.3		
Model composition			
Non‐hydrogen atoms	11,468		
Protein residues	1,402		
Ligands	Ca^2+^: 4		
B factors (Å^2^)			
Protein	54.4		
Ligand	33.7		
r.m.s. deviations			
Bond lengths (Å)	0.004		
Bond angles (°)	0.813		
Validation			
MolProbity score	1.46		
Clash score	3.67		
Poor rotamers (%)	0.00		
Ramachandran plot			
Favored (%)	95.6		
Allowed (%)	4.40		
Disallowed (%)	0.00		

^a^ Values in parentheses indicate the pixel size in super‐resolution.

**Figure EV3:**
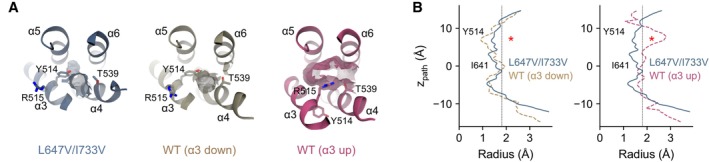
Pore dimension Molecular surface of the extracellular vestibule viewed from the top of the membrane. Selected residues lining the volume are shown. The bound 1PBC molecule in the WT (α3 up) model is omitted in the display for clarity. The models are as in Fig 4C.Pore radius along the z‐axis relative to the position of Ile 641 (gate). The locations of constrictions are indicated. Asterisk indicates the location of the 1PBC‐binding site. Dashed line denotes the ionic radius of a Cl^−^ ion.

Single‐particle analysis of L647V/I733V reveals a major conformation that is distinct from the wild‐type channel (Fig EV2). With an overall resolution of 3.29 Å, the final map shows well‐defined density for the entire protein, including the bound agonist Ca^2+^ in its principal binding site (Fig EV2G and H). In this structure, the disruption of the L647/I733 interaction results in an incomplete rearrangement of α6, with the helix adopting an intermediate, partially activated conformation (Fig 4C and D). The described change in conformation is accompanied by a loss of the density corresponding to the Ca^2+^‐binding part of α6, indicating that the helix has become more mobile and that the interaction with bound Ca^2+^ is substantially weakened (Fig EV2G and H). The incomplete rearrangement of α6 is accompanied by a partial collapse of the extracellular part of the pore‐lining helix α4 toward α6 (Fig 4C, left and Fig EV3), which is evident when compared with the 1PBC‐bound, outer‐pore‐open conformation (Fig 4C, right and Fig EV3). This might in part explain the elevated steric barriers for ion conduction and iblocker access in this mutant as observed in electrophysiological experiments (Fig 3A, C and F).

As a consequence of the weakened interaction between α6 and α8, the protein is in a state containing a single well‐resolved Ca^2+^ ion in the principal site, even at a saturating Ca^2+^concentration, suggesting that in the mutant, this site might accommodate only one ligand (Figs 4E and EV2H). The concomitance of these structural changes has also been observed for the wild‐type channel solubilized in LMNG, although its relevance to channel function has remained unclear (Dang *et al*, 2017). The single‐bound Ca^2+^ is in a position similar to the lower ion in the doubly bound state and is coordinated by two acidic residues, Glu 705 on α7 and Asp 738 on α8, with no coordinating residues from α6 since its rearrangement is impaired (Fig 4D and E). Despite in a Ca^2+^‐bound state, the region below the conserved glycine hinge remains an α‐helix (Fig 4F), suggesting that the L647/I733 interaction and the second bound Ca^2+^ in the upper position are needed to stabilize the strained π‐helix conformation corresponding to α6 in a fully activated state. Reciprocally, the formation of a π‐helix in this region would bring these residues into the correct register that maximizes the respective protein‐ligand and α6‐α8 interactions. Together, these structural data suggest that the L647/I733 interaction promotes pore opening and contributes to the positive cooperativity of ligand binding by priming and stabilizing the complete closure of the binding site.

### Conformational coupling between the agonist‐binding site and the gate underlies ligand efficacy

**Figure 5:**
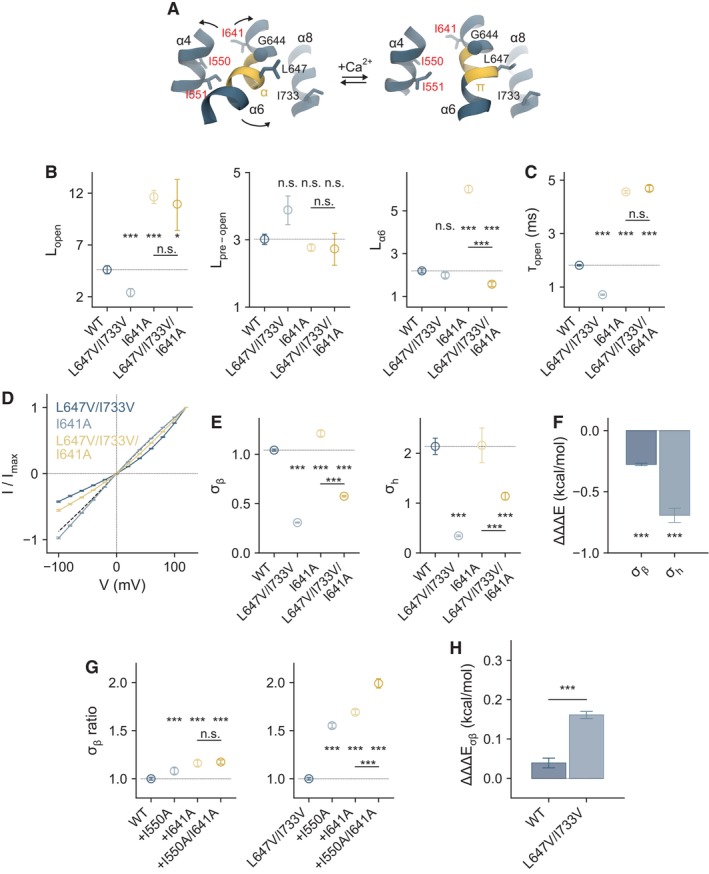
Coupling binding site closure and α6 rearrangement to channel opening Rearrangements of the gate region and around the binding site upon Ca^2+^ binding. Selected helices are shown as ribbon, sidechains as sticks, and the Cα of Gly 644 as sphere. The region undergoing an α‐to‐π‐helix transition is highlighted in yellow. Residues forming the gate are labeled in red. PDBID 5OYG (Ca^2+^‐free) and 5OYB (Ca^2+^‐bound) (Paulino *et al*, 2017a) are displayed.Forward equilibrium constants for the indicated transitions obtained from autocorrelation analysis (Equations 7–9 in Supplementary Methods, see Appendix Fig S1). Data are best‐fit values of the averaged spectra from 7, 5, 7, and 6 patches for WT, L647V/I733V, I641A, and L647V/I733V/I641A respectively, and errors are 95% confidence intervals. Dashed line indicates the value of wild‐type. *t*‐test: n.s., nonsignificant; *P < 0.05; ***P < 0.005.Mean open time calculated from the estimated rate constant $k_{01}$. Data were calculated from the best‐fit values, and errors are 95% confidence intervals. The number of replicates is as in (B). *t*‐test: n.s., nonsignificant; ***P < 0.005.Instantaneous I‐V relations of the indicated mutants at a saturating Ca^2+^ concentration. Data are averages of 6, 6, 7, and 13 patches for WT, L647V/I733V, I641A, and L647V/I733V/I641A respectively, and errors are SEM. Solid lines are fits to a model of ion permeation (Equation 1 in Supplementary Methods) shown in Fig 3B. Dashed line is the relation of wild‐type.Conduction parameters for the indicated barriers. Data are best‐fit values, and errors are 95% confidence intervals. Dashed line indicates the value of wild‐type. The number of replicates is as in (D). *t*‐test: ***P < 0.005.Coupling energies (ΔΔΔE) for the indicated barriers. Bars indicate quantities calculated using Equations 2 and 10 in Supplementary Methods, and errors are standard errors. The number of replicates is as in (D). *t*‐test: ***P < 0.005.σ_β_ ratios of the indicated mutants on the wild‐type or L647V/I733V background. Data are calculated from the best‐fit values, and errors are 95% confidence intervals. Data were obtained from 6, 6, 7, and 10 patches for WT, I550A, I641A, and I550A/I641A respectively (Left) and from 10, 8, 13, and 9 patches for L647V/I733V, L647V/I733V/I550A, L647V/I733V/I641A, and L647V/I733V/I550A/I641A respectively (Right). *t*‐test: n.s., nonsignificant; ***P < 0.005.Coupling energies (ΔΔΔE) for the inner barrier. Bars indicate quantities calculated using Equations 2 and 10 in Supplementary Methods, and errors are standard errors. The number of replicates is as in (G). *t*‐test: ***P < 0.005. Source data are available online for this figure.

**Figure EV4:**
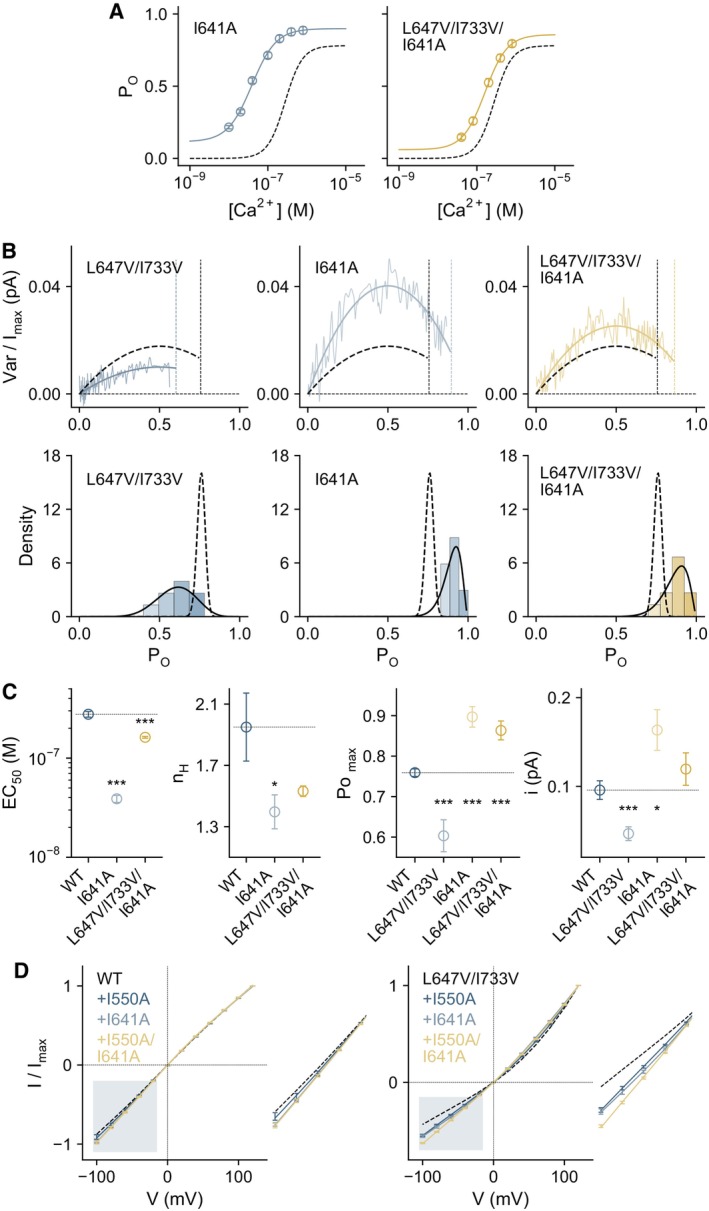
Activation and conduction properties of mutants Concentration‐Po relations for the indicated mutants at +80 mV. Data are averages of 10 and 7 patches for I641A and L647V/I733V/I641A respectively, and errors are SEM. Solid line is a fit to the Hill equation. Dashed line is the relation of wild‐type.Top, merged and averaged variance‐current relations at a saturating Ca^2+^ concentration at −40 mV. Data are averages of 10, 6, and 10 patches for L647V/I733V, I641A, and L647V/I733V/I641A respectively. Solid line is a fit to Equation 5 in Supplementary Methods. Dashed line is the relation of wild‐type. Dotted lines indicate the maximum Po. Bottom, histograms of the maximum Po obtained from individual measurements. Solid line is a fit to the beta distribution. Dashed line is the distribution of wild‐type.EC_50_, n_H_, Po_max_, and i of the indicated constructs. For EC_50_ and n_H_, data are averages of 8, 10, and 7 patches for WT, I641A, and L647V/I733V/I641A respectively, and errors are SEM. For Po_max_ and i, data are averages of 8, 10, 6, and 10 patches for WT, L647V/I733V, I641A, and L647V/I733V/I641A respectively, and errors are SEM. *t*‐test: *P < 0.05; ***P < 0.005.Instantaneous I‐V relations of the indicated mutants at a saturating Ca^2+^ concentration. Data are averages of 6, 6, 7, and 10 patches for WT, I550A, I641A, and I550A/I641A respectively (Left) and of 10, 8, 13, and 9 patches for L647V/I733V, L647V/I733V/I550A, L647V/I733V/I641A, and L647V/I733V/I550A/I641A respectively (Right), and errors are SEM. Solid lines are fits to a model of ion permeation (Equation 1 in Supplementary Methods) shown in Fig 3B. Dashed line is the relation of wild‐type.

In TMEM16A, the channel gate is located at the intracellular pore entrance and is formed by three pore‐lining hydrophobic residues (Ile 550, 551, and 641) residing on α‐helices 4 and 6, with Ile 641 being the most prominent contributor (Lam *et al*, 2021; Lam & Dutzler, 2021). Since Ile 641 is one helix turn above the glycine hinge on α6 and the L647/I733 interaction takes place one helix turn below, we hypothesized that defective rearrangements below the hinge may exert a direct influence on the functioning of the gate (Fig 5A). We investigated this scenario by constructing a double‐mutant cycle consisting of L647V/I733V and I641A and analyzed whether the effect of L647V/I733V on decreasing efficacy depends on the stability of the gate (Figs 5 and EV4). Consistent with this hypothesis, L647V/I733V is unable to reduce the efficacy of pore opening and shorten the open state lifetime in the mutant I641A, where the disruption of the gate has profoundly stabilized the open state. This is reflected in the similar values of the respective L_open_ and τ_open_, revealing a functional coupling between the agonist‐coupled movements and the opening of the gate (Fig 5B and C, Appendix Table S3). Reciprocally, I641A, which opens the gate, fails to promote agonist‐coupled rearrangements when the L647/I733 interaction is impaired in the mutant L647V/I733V, yielding a similar value of L_α6_ as WT (Fig 5B, Appendix Table S3). This is in sharp contrast to the strongly increased transition observed in the single alanine mutant of this residue.

The interdependence between the gate and α6 rearrangement extends to the extent of pore opening (i.e., the size of the open pore), in which I641A partially reversed the inner pore collapse caused by L647V/I733V (Fig 5D–F). This is manifested in the somewhat reduced current rectification of the triple mutant (Fig 5D) and the magnitudes of the inner and central barriers for ion conduction (Fig 5E), where the effect of I641A is more pronounced on the L647V/I733V background compared to WT. This indicates that the more constricted pore in L647V/I733V is due to a partial obstruction by the gate residue Ile 641, which in turn suggests an incomplete release of the gate in the open state of this mutant. The disparity in the effect of I641A on the L647V/I733V and WT backgrounds in both gating (Fig 5B and C) and ion conduction (Fig 5D and E) results in nonzero coupling energies (Fig 5F), indicating that the opening of the gate and α6 rearrangement do not operate independently but that their conformational transitions are coupled.

The decrease in the opening efficacy and the incomplete release of the gate prompted us to investigate whether these are associated with a tighter interaction within the gate when α6 rearrangement is impaired. To test this, we constructed a double‐mutant cycle consisting of the two gate residues I550A on α4 and I641A on α6 on the L647V/I733V and WT backgrounds and compared the respective coupling energies between Ile 550 and Ile 641 derived from the magnitudes of the inner barrier in the open pore (Figs 5G and EV4D). Compared to the WT background, the coupling energy between the gate residues Ile 550 and Ile 641 is increased by about 4‐fold in the L647V/I733V background (Fig 5H), indicating that these gate residues indeed interact more tightly when α6 rearrangement is disrupted. Together, these results suggest a mechanism in which the agonist‐coupled rearrangements weaken interactions within the inner pore gate and vice versa.

## Discussion

**Figure 6:**
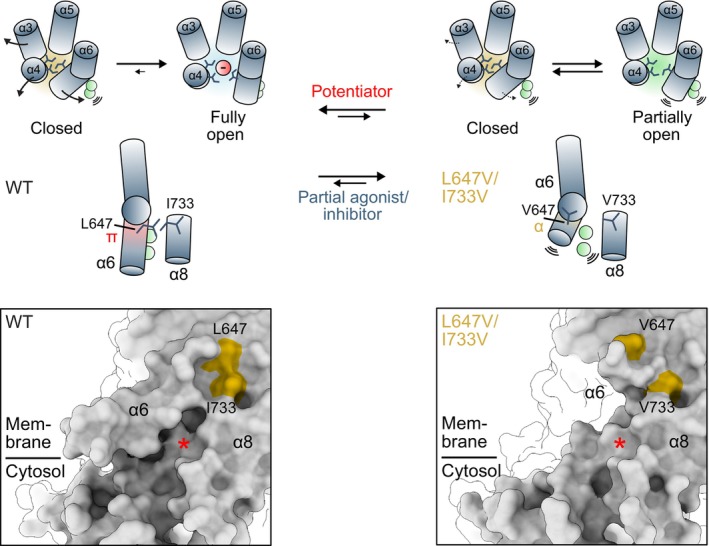
Mechanism and pharmacological implications Achieving maximum Po in the fully liganded state (i.e. at saturating Ca^2+^ concentrations) requires α6 in its fully activated conformation, which involves a transition into a π‐helix that is stabilized by Ca^2+^ binding and the interaction between Leu 647 and Ile 733. This creates a local structural environment that favors a complete release of the inner pore gate, ensuring a full open probability and channel conductance. Stabilization of the activated conformation of α6 is expected to potentiate TMEM16A activity, while destabilizing α6 would result in partial agonism or inhibition, through modulation of both the gating equilibrium and channel conductance. Potential druggable region for rationally designed TMEM16A modulators is shown in the inset (red asterisk). Left, PDBID 7ZK3 (Lam *et al*, 2022).

**Figure EV5:**
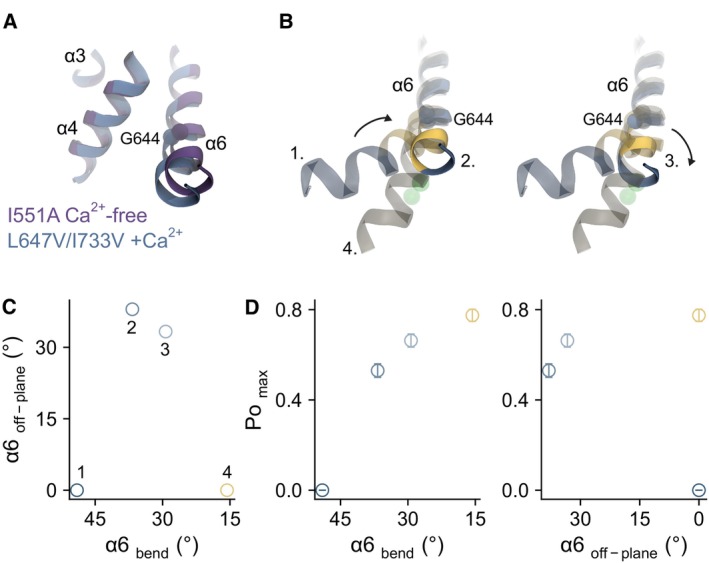
α6 conformation and efficacy Superposition of the I551A Ca^2+^‐free (PDBID 7B5D) and L647V/I733V + Ca^2+^ models. Selected helices are shown. The Cα of Gly 644 is shown as sphere.α6 conformations along its activation pathway. 1. WT Ca^2+^‐free (PDBID 5OYG), 2. I551A Ca^2+^‐free (PDBID 7B5D), 3. L647V/I733V + Ca^2+^, and 4. WT + Ca^2+^ (PDBID 5OYB). The Cα of Gly 644 is shown as sphere. The bound Ca^2+^ in the wild‐type model are shown as green spheres.Bending and off‐plane movements below Gly 644 along the activation trajectory of α6. The plane defined by WT Ca^2+^‐free and WT + Ca^2+^ was used as a reference (0°).Relationship between efficacy and α6 conformations.

In this study, we combined kinetic analysis and structural investigations to address the molecular basis governing agonist efficacy in the ligand‐gated channel TMEM16A. Using mutagenesis and autocorrelation analysis, we have shown that disrupting agonist‐coupled rearrangements at the binding site allosterically impairs channel opening by decreasing the stability of the open state (Figs 1 and 2). The structure of a mutant with defective binding site rearrangement reveals a distinct conformational change that underlies the decrease in the channel's opening efficacy and suggests a role for the α‐to‐π‐helix transition of α6 in structurally coupling binding site closure and the release of the inner pore gate (Fig 4). This mechanism is supported by the analysis of several double‐mutant cycles, showing that agonist‐dependent α6 closure destabilizes the hydrophobic gate at the inner pore, thereby enhancing the efficacy and the degree of channel opening (Figs 3 and 5). Our data, therefore, provide direct experimental evidence that binding site closure is linked to agonist efficacy via the conformation of α6 (Figs 6 and EV5).

Binding site closure upon ligand binding is a general phenomenon in LGICs, notably in pentameric neurotransmitter‐gated channels (pLGICs) and ionotropic glutamate receptors (Du *et al*, 2015; Plested, 2016; Twomey & Sobolevsky, 2018; Yu *et al*, 2021). In TMEM16A, this process involves an agonist‐coupled rearrangement of α6 that brings an essential glutamate (Glu 654) into direct contact with the bound Ca^2+^ (Paulino *et al*, 2017a). This movement is enabled by an α‐to‐π‐helix transition below the conserved glycine hinge and is additionally stabilized by a hydrophobic interaction between α6 and α8 in the π‐helical region, mediated by Leu 647 and Ile 733 (Fig 1). Van der Waals attraction between these two residues, which are closely apposed, likely stabilizes the fully activated conformation of α6 as its rearrangement cannot be completed when their sidechains are truncated, with the ensuing portion of this helix becoming highly flexible (Fig 4). This primes and couples the α‐to‐π‐helix transition and the binding of the second Ca^2+^, both of which are subsequently stabilized by the Ca^2+^‐binding residues on α6, Asn 650 and Glu 654 (Fig 4). The straightened π‐helical conformation of α6 likely relieves structural constraints in the gate region, thereby allowing a more extended opening and a prolongation of the open lifetime (Figs 2, 3 and 5).

The coupling between α6 rearrangement and the gate is likely bidirectional, as loosening the gate reciprocally enhances this agonist‐promoted transition (Fig 5) and a direct disruption of the gate region results in an almost superimposable, partially activated conformation of α6 even in the absence of Ca^2+^ (Lam *et al*, 2021). A comparison of the available structures of TMEM16A with different open probabilities reveals a correlation between the degree of binding site closure and opening efficacy, with the π‐helical conformation of α6 appearing to be central to this process (Fig EV5). The correspondence between incomplete binding site closure and impeded channel opening in L647V/I733V, where α6 rearrangement is impaired, and the previously described apo I551A (Lam *et al*, 2021), where the disruption of the gate results in a partial activation of α6 even in the absence of Ca^2+^ (Fig EV5A), suggests a general mechanism that governs agonist efficacy in TMEM16A. The immediate relevance of the tight interactions between α6 and α8 to channel opening highlights the combined molecular surface of both helices intracellular to the Leu 647/Ile 733 pair as a promising druggable region for the design of channel modulators (Fig 6), where molecules stabilizing this interaction would presumably potentiate channel activity at physiological Ca^2+^ concentrations. Conversely, destabilization of this conformation is likely to result in partial agonism/antagonism through a combined effect on both the gating equilibrium and channel conductance.

Agonist‐bound intermediate closed states have been observed in several pLGICs, notably in nicotinic and glycine receptors (Lape *et al*, 2008; Mukhtasimova *et al*, 2009). These flipped or primed states display a higher affinity for the ligand, likely reflecting a rearrangement of the binding site. The ability of these channels to open in response to different ligands appears to be governed by the stability of these activated, pre‐open states relative to that of the agonist‐bound resting state, while the opening step remains equally efficacious irrespective of the efficacy of the ligands (Lape *et al*, 2008). Despite its distinct structural organization, channel opening in TMEM16A is also preceded by pre‐open or flipped states that reflect the activation of α6 and the closure of the binding site; a conformational change that likely underlies the higher Ca^2+^ affinity in these states (Lam & Dutzler, 2021). By contrast, the relative stability of the open state in TMEM16A is dictated by the degree of binding site closure, with incomplete closure resulting in channels that open with lower probability (Figs 1, 2, 4 and EV5). It seems, therefore, that unlike the robustness of the ECD‐TMD interface in pLGICs when bound to ligands with different efficacy (Yu *et al*, 2021; Ivica *et al*, 2022), the profound local structural changes between the closely apposed ligand binding and pore modules in TMEM16A may naturally give rise to a more obligatory coupling between binding site closure and channel opening.

In summary, our kinetic and structural analyses have allowed us to establish a relationship between agonist efficacy and binding site rearrangements in TMEM16A (Fig 6), a ligand‐gated channel built on a distinct architecture that is conserved throughout the TMEM16 family. The coupling between channel opening and the closure of the binding site originates from the ability of Ca^2+^ to stabilize an otherwise energetically costly π‐helical conformation to create a structural environment that favors channel opening. A related mechanism might underlie agonist binding and gating in TMEM16 scramblases, where more global conformational changes are propagated from the Ca^2+^‐binding site (Bushell *et al*, 2019; Falzone *et al*, 2019; Kalienkova *et al*, 2019; Arndt *et al*, 2022), and channels sharing a similar molecular scaffold such as the OSCA and TMC families of ion channels (Jojoa‐Cruz *et al*, 2018; Zhang *et al*, 2018; Maity *et al*, 2019; Jeong *et al*, 2022). The structures of TMEM16A and its mutants with different efficacy, described here and previously, thus provide an important basis for the development of novel potentiators and partial agonists in the TMEM16 family.

## Materials and Methods

### Molecular biology and cell culture

HEK293T cells (ATCC CRL‐1573) were maintained in Dulbecco's modified Eagle's medium (DMEM; Sigma‐Aldrich) supplemented with 100 U/ml penicillin, 0.1 mg/ml streptomycin (Sigma‐Aldrich), 2 mM L‐glutamine (Sigma‐Aldrich), and 10% FBS (Sigma‐Aldrich) in a humidified atmosphere containing 5% CO_2_ at 37°C. HEK293S GnTI^−^ cells (ATCC CRL‐3022) were maintained in HyClone HyCell TransFx‐H medium (Cytiva) supplemented with 100 U/ml penicillin, 0.1 mg/ml streptomycin, 4 mM L‐glutamine, 0.15% poloxamer 188 (Sigma‐Aldrich), and 1% FBS in an atmosphere containing 5% CO_2_ at 185 rpm at 37°C. The cell lines were obtained from commercial sources and further authentication was not performed. The cell lines were tested and are free from mycoplasma contamination. The *ac* splice variant of mouse TMEM16A (UniProt ID: Q8BHY3) bearing a 3C cleavage site, a Venus YFP, a Myc tag, and a Streptavidin‐binding peptide (SBP) downstream of the open reading frame in a modified pcDNA3.1 vector (Invitrogen) was used as described previously (Lim *et al*, 2016). Mutations were introduced using a modified QuikChange method (Zheng *et al*, 2004) with primers described previously (Lam *et al*, 2021) and were verified by sequencing.

### Protein expression and purification

HEK293S GnTI^−^ cells were transiently transfected with mouse TMEM16A‐L647V/I733V complexed with polyethylenimine MAX 40 K (formed in nonsupplemented DMEM medium at a w/w ratio of 1:2.5 for 30 min). Immediately after transfection, the culture was supplemented with 3.5 mM valproic acid. Cells were collected 48 h post‐transfection, washed with PBS, and stored at −80°C until further use. Protein purification was carried out at 4°C and was completed within 12 h. The protein was purified in Ca^2+^‐free buffers and was supplemented with 1 mM free Ca^2+^ when indicated during cryo‐EM sample preparation. Cells were resuspended and solubilized in 150 mM NaCl, 5 mM EGTA, 20 mM HEPES, 1x cOmplete protease inhibitors (Roche), 40 μg/ml DNase (AppliChem), and 2% GDN (Anatrace) at pH 7.4 by gentle mixing for 2 h. The solubilized fraction was obtained by centrifugation at 16,000 *g* for 30 min. After filtration with 0.5 μm filters (Sartorius), the supernatant was incubated with streptavidin UltraLink resin (Pierce, Thermo Fisher Scientific) for 2 h under gentle agitation. The beads were loaded onto a gravity column and were washed with 60 column volumes of SEC buffer containing 150 mM NaCl, 2 mM EGTA, 20 mM HEPES, 0.01% GDN at pH 7.4. The bound protein was eluted by incubating the beads with 3 column volumes of SEC buffer supplemented with 0.25 mg/ml 3C protease for 30 min. The eluate was concentrated using a 100 kDa cutoff filter, filtered through a 0.22 μm filter, and loaded onto a Superose 6 10/300 GL column (Cytiva) pre‐equilibrated with SEC buffer. Peak fractions containing the protein were pooled, concentrated, and used immediately for cryo‐EM sample preparation.

### Cryo‐EM sample preparation

2.5 μl of purified protein, concentrated to ~1.7 mg/ml and supplemented with 1 mM free Ca^2+^ immediately before plunge‐freezing, was applied onto holey carbon grids (Quantifoil Au R1.2/1.3, 300 mesh) or holey gold grids (UltrAuFoil R1.2/1.3, 300 mesh) for the noncoated datasets collected at 0° and 20° tilt, respectively. Immediately prior to sample application, the grids were glow discharged at 15 mA for 30 s. After sample application, the grids were blotted for 2–4 s with a blot force setting of 0 at 4°C at 100% humidity, plunge‐frozen in a liquid propane/ethane mixture using Vitrobot Mark IV (Thermo Fisher Scientific), and stored in liquid nitrogen until further use. For the dataset collected on graphene oxide (GO) support, samples were applied to the back side of UltrAuFoil grids deposited with GO (Sigma‐Aldrich) on the front side at the Vitrobot according to (Cheung *et al*, 2018). In this case, the samples and grids were prepared as above except that the purified protein was used at ~0.5 mg/ml and that the grids were blotted for 2 s with a blot force setting of 7.

### Cryo‐EM data acquisition

Data collection was performed on a 300 kV Titan Krios G3i (Thermo Fisher Scientific) equipped with a post‐column quantum energy filter (20 eV slit width) and a K3 summit direct electron detector (Gatan) in super‐resolution mode. Dose‐fractionated micrographs were collected at a nominal magnification of 130,000× corresponding to a pixel size of 0.659 Å/pixel (0.3295 Å/pixel in super‐resolution) and a nominal defocus range of −1 to −2.4 μm using EPU 2.9 (Thermo Fisher Scientific). For datasets collected on noncoated grids at 0° and 20° tilt, each movie contained 36 frames with a total exposure time of 1 s and a total dose of approximately 66.1 e^−^ Å^−2^ (1.85 e^−^ Å^−2^ frame^−1^). For datasets collected on GO support, each movie contained 47 frames and were binned two times on the fly, with a total exposure time of 1.26 s and a total dose of approximately 62.5 e^−^ Å^−2^ (1.33 e^−^ Å^−2^ frame^−1^).

### Cryo‐EM data processing

The datasets were processed in RELION 3.1 (Zivanov *et al*, 2018). Micrographs were preprocessed using RELION's own implementation of MotionCor2 (Zheng *et al*, 2017), and Gctf (Zhang, 2016). crYOLO (Wagner *et al*, 2019) was used for automated particle picking, resulting in 896,659, 297,015, and 926,305 particle images, respectively, for the 0°, 20°, and GO datasets (6,924 (binned 2×), 7,167 (binned 2×), and 17,507 movies respectively). Particles were extracted with a box size of 480 pixels with 3× binning (160‐pixel box, 1.977 Å/pixel) and were subjected to two to three rounds of 2D classification, separately for each dataset. Selected classes were pooled, resulting in 317,782 particles (126,229, 8,584, and 182,969 from the 0°, 20°, and GO datasets respectively) and were 3D‐classified without symmetry applied using a previous Ca^2+^‐bound TMEM16A map low‐pass filtered to 20 Å as a reference. Particles from the best classes (103,964, 6,242, and 34,728 from the 0°, 20°, and GO datasets respectively) were re‐extracted with a box size of 400 pixels unbinned (0.659 Å/pixel) and were refined with C2 symmetry applied. A final map resolved to 3.29 Å was obtained after several rounds of CTF refinement and Bayesian polishing (separately for datasets with different electron dose), and a masked refinement excluding the detergent micelle upon convergence in the final refinement.

### Model building, refinement, and validation

The initial model was obtained by fitting the previously determined Ca^2+^‐free TMEM16A‐I551A structure (Lam *et al*, 2021) (PDBID 7B5D) into the density of the Ca^2+^‐bound TMEM16A‐L647V/I733V using Chimera (Pettersen *et al*, 2004), which was then iteratively rebuilt in Coot (Emsley & Cowtan, 2004) and refined in Phenix (Afonine *et al*, 2018). The geometry of the final models was evaluated using MolProbity (Williams *et al*, 2018). Global and directional Fourier shell correlations (FSCs) between the half‐maps were estimated using the 3DFSC server (https://3dfsc.salk.edu/) (Tan *et al*, 2017). Cross‐validation was performed by evaluating FSC_work_, the FSC between the final model with random shifts of up to 3 Å applied refined against one of the half‐maps and this half‐map, and FSC_free_, the FSC between the resulting model and the other half‐map. Figures containing molecular structures and maps were prepared using VMD (Humphrey *et al*, 1996) and ChimeraX (Pettersen *et al*, 2021). Angular difference of the principal axes of selected helices in superposed structures was calculated in VMD.

### Electrophysiology

HEK293T cells were transfected with 3–4 μg DNA per 6 cm Petri dish using the calcium phosphate co‐precipitation method and were used within 24–96 h after transfection. Recordings were performed on inside‐out patches excised from HEK293T cells expressing the construct of interest. Patch pipettes were pulled from borosilicate glass capillaries (O.D. 1.5 mm, I.D. 0.86 mm, Sutter Instrument) and were fire‐polished with a microforge (Narishige) before use. Pipette resistance was typically 3–8 MΩ when filled with the recording solutions detailed below. Seal resistance was typically 4 GΩ or higher. Voltage‐clamp recordings were made using Axopatch 200B, Digidata 1550, and Clampex 10.7 (Molecular Devices). Analog signals were filtered with the in‐built 4‐pole Bessel filter at 10 kHz and were digitized at 20 kHz. Solution exchange was achieved using a gravity‐fed system through a theta glass pipette mounted on an ultra‐fast piezo‐driven stepper (Siskiyou). Liquid junction potential was found to be consistently negligible given the ionic composition of the solutions and was, therefore, not corrected. All recordings were performed at 20°C.

A symmetrical ionic condition was used throughout. Stock solution with Ca^2+^‐EGTA contained 150 mM NaCl, 5.99 mM Ca(OH)_2_, 5 mM EGTA, and 10 mM HEPES at pH 7.40. Stock solution with EGTA contained 150 mM NaCl, 5 mM EGTA, and 10 mM HEPES at pH 7.40. Free Ca^2+^ concentrations were adjusted by mixing the stock solutions at the required ratios calculated using the WEBMAXC program (http://web.stanford.edu/~cpatton/webmaxcS.htm). Patch pipettes were filled with the stock solution with Ca^2+^‐EGTA, which has a free Ca^2+^ concentration of 1 mM. Unless otherwise stated, experiments were performed at a saturating Ca^2+^ concentration, and the primary data were corrected for current rundown as described previously (Lim *et al*, 2016; Lam & Dutzler, 2018). Analysis of the electrophysiology data is described in detail in the Supplementary Methods.

## Author contributions

**Andy KM Lam:** Conceptualization; data curation; formal analysis; funding acquisition; validation; investigation; visualization; methodology; writing – original draft; writing – review and editing. **Raimund Dutzler:** Supervision; funding acquisition; project administration; writing – review and editing.

## Disclosure and competing interests statement

The authors declare that they have no conflict of interest.

## Supporting information



Appendix

Expanded View Figures PDF

PDF+

Source Data for Figure 1

Source Data for Figure 2

Source Data for Figure 3

Source Data for Figure 5

## Data Availability

Data supporting the findings of this study are available from the corresponding authors upon reasonable request. The cryo‐EM map, half‐maps, and mask have been deposited in the Electron Microscopy Data Bank under accession number EMD‐18774 (http://www.ebi.ac.uk/pdbe/entry/EMD‐18774). Coordinates for the model are available in the Protein Data Bank under PDB ID 8QZC (http://www.rcsb.org/pdb/explore/explore.do?structureId=8QZC). Source data are provided with this paper.

## References

[embj2023115030-bib-0001] Afonine PV , Poon BK , Read RJ , Sobolev OV , Terwilliger TC , Urzhumtsev A , Adams PD (2018) Real‐space refinement in PHENIX for cryo‐EM and crystallography. Acta Crystallogr D Struct Biol 74: 531–544 29872004 10.1107/S2059798318006551 PMC6096492

[embj2023115030-bib-0002] Al‐Hosni R , Ilkan Z , Agostinelli E , Tammaro P (2022) The pharmacology of the TMEM16A channel: therapeutic opportunities. Trends Pharmacol Sci 43: 712–725 35811176 10.1016/j.tips.2022.06.006

[embj2023115030-bib-0003] Arndt M , Alvadia C , Straub MS , Clerico Mosina V , Paulino C , Dutzler R (2022) Structural basis for the activation of the lipid scramblase TMEM16F. Nat Commun 13: 6692 36335104 10.1038/s41467-022-34497-x PMC9637102

[embj2023115030-bib-0004] Arreola J , Hartzell HC (2019) Wasted TMEM16A channels are rescued by phosphatidylinositol 4,5‐bisphosphate. Cell Calcium 84: 102103 31683182 10.1016/j.ceca.2019.102103 PMC6913893

[embj2023115030-bib-0005] Bushell SR , Pike ACW , Falzone ME , Rorsman NJG , Ta CM , Corey RA , Newport TD , Christianson JC , Scofano LF , Shintre CA *et al* (2019) The structural basis of lipid scrambling and inactivation in the endoplasmic reticulum scramblase TMEM16K. Nat Commun 10: 3956 31477691 10.1038/s41467-019-11753-1 PMC6718402

[embj2023115030-bib-0006] Caputo A , Caci E , Ferrera L , Pedemonte N , Barsanti C , Sondo E , Pfeffer U , Ravazzolo R , Zegarra‐Moran O , Galietta LJ (2008) TMEM16A, a membrane protein associated with calcium‐dependent chloride channel activity. Science 322: 590–594 18772398 10.1126/science.1163518

[embj2023115030-bib-0007] Cheung M , Adaniya H , Cassidy C , Yamashita M , Li KL , Taba S , Shintake T (2018) Improved sample dispersion in cryo‐EM using “perpetually‐hydrated” graphene oxide flakes. J Struct Biol 204: 75–79 30030043 10.1016/j.jsb.2018.07.008

[embj2023115030-bib-0008] Colquhoun D (1998) Binding, gating, affinity and efficacy: the interpretation of structure‐activity relationships for agonists and of the effects of mutating receptors. Br J Pharmacol 125: 924–947 9846630 10.1038/sj.bjp.0702164 PMC1565672

[embj2023115030-bib-0009] Danahay HL , Lilley S , Fox R , Charlton H , Sabater J , Button B , McCarthy C , Collingwood SP , Gosling M (2020) TMEM16A potentiation: a novel therapeutic approach for the treatment of cystic fibrosis. Am J Respir Crit Care Med 201: 946–954 31898911 10.1164/rccm.201908-1641OC PMC7159426

[embj2023115030-bib-0010] Dang S , Feng S , Tien J , Peters CJ , Bulkley D , Lolicato M , Zhao J , Zuberbuhler K , Ye W , Qi L *et al* (2017) Cryo‐EM structures of the TMEM16A calcium‐activated chloride channel. Nature 552: 426–429 29236684 10.1038/nature25024 PMC5750132

[embj2023115030-bib-0011] Dinsdale RL , Pipatpolkai T , Agostinelli E , Russell AJ , Stansfeld PJ , Tammaro P (2021) An outer‐pore gate modulates the pharmacology of the TMEM16A channel. Proc Natl Acad Sci USA 118: e2023572118 34413188 10.1073/pnas.2023572118 PMC8403959

[embj2023115030-bib-0012] Du J , Lu W , Wu S , Cheng Y , Gouaux E (2015) Glycine receptor mechanism elucidated by electron cryo‐microscopy. Nature 526: 224–229 26344198 10.1038/nature14853 PMC4659708

[embj2023115030-bib-0013] Emsley P , Cowtan K (2004) Coot: model‐building tools for molecular graphics. Acta Crystallogr D Biol Crystallogr 60: 2126–2132 15572765 10.1107/S0907444904019158

[embj2023115030-bib-0014] Falzone ME , Rheinberger J , Lee BC , Peyear T , Sasset L , Raczkowski AM , Eng ET , Di Lorenzo A , Andersen OS , Nimigean CM *et al* (2019) Structural basis of Ca^2+^‐dependent activation and lipid transport by a TMEM16 scramblase. Elife 8: e43229 30648972 10.7554/eLife.43229 PMC6355197

[embj2023115030-bib-0015] Galietta LJV (2022) TMEM16A (ANO1) as a therapeutic target in cystic fibrosis. Curr Opin Pharmacol 64: 102206 35364521 10.1016/j.coph.2022.102206

[embj2023115030-bib-0016] Grosman C , Zhou M , Auerbach A (2000) Mapping the conformational wave of acetylcholine receptor channel gating. Nature 403: 773–776 10693806 10.1038/35001586

[embj2023115030-bib-0017] Gupta S , Chakraborty S , Vij R , Auerbach A (2017) A mechanism for acetylcholine receptor gating based on structure, coupling, phi, and flip. J Gen Physiol 149: 85–103 27932572 10.1085/jgp.201611673 PMC5217088

[embj2023115030-bib-0018] Huang F , Zhang H , Wu M , Yang H , Kudo M , Peters CJ , Woodruff PG , Solberg OD , Donne ML , Huang X *et al* (2012) Calcium‐activated chloride channel TMEM16A modulates mucin secretion and airway smooth muscle contraction. Proc Natl Acad Sci USA 109: 16354–16359 22988107 10.1073/pnas.1214596109 PMC3479591

[embj2023115030-bib-0019] Humphrey W , Dalke A , Schulten K (1996) VMD: visual molecular dynamics. J Mol Graph 14: 27–38 10.1016/0263-7855(96)00018-5 8744570

[embj2023115030-bib-0020] Ivica J , Zhu H , Lape R , Gouaux E , Sivilotti LG (2022) Aminomethanesulfonic acid illuminates the boundary between full and partial agonists of the pentameric glycine receptor. Elife 11: e79148 35975975 10.7554/eLife.79148 PMC9462852

[embj2023115030-bib-0021] Jadey S , Auerbach A (2012) An integrated catch‐and‐hold mechanism activates nicotinic acetylcholine receptors. J Gen Physiol 140: 17–28 22732309 10.1085/jgp.201210801 PMC3382718

[embj2023115030-bib-0022] Jeng G , Aggarwal M , Yu WP , Chen TY (2016) Independent activation of distinct pores in dimeric TMEM16A channels. J Gen Physiol 148: 393–404 27799319 10.1085/jgp.201611651 PMC5089935

[embj2023115030-bib-0023] Jeong H , Clark S , Goehring A , Dehghani‐Ghahnaviyeh S , Rasouli A , Tajkhorshid E , Gouaux E (2022) Structures of the TMC‐1 complex illuminate mechanosensory transduction. Nature 610: 796–803 36224384 10.1038/s41586-022-05314-8 PMC9605866

[embj2023115030-bib-0024] Jia Z , Chen J (2021) Specific PIP2 binding promotes calcium activation of TMEM16A chloride channels. Commun Biol 4: 259 33637964 10.1038/s42003-021-01782-2 PMC7910439

[embj2023115030-bib-0025] Jojoa‐Cruz S , Saotome K , Murthy SE , Tsui CCA , Sansom MS , Patapoutian A , Ward AB (2018) Cryo‐EM structure of the mechanically activated ion channel OSCA1.2. Elife 7: e41845 30382939 10.7554/eLife.41845 PMC6235563

[embj2023115030-bib-0026] Kalienkova V , Clerico Mosina V , Bryner L , Oostergetel GT , Dutzler R , Paulino C (2019) Stepwise activation mechanism of the scramblase nhTMEM16 revealed by cryo‐EM. Elife 8: e44364 30785398 10.7554/eLife.44364 PMC6414200

[embj2023115030-bib-0027] Lam AK , Dutzler R (2018) Calcium‐dependent electrostatic control of anion access to the pore of the calcium‐activated chloride channel TMEM16A. Elife 7: e39122 30311910 10.7554/eLife.39122 PMC6195346

[embj2023115030-bib-0028] Lam AKM , Dutzler R (2021) Mechanism of pore opening in the calcium‐activated chloride channel TMEM16A. Nat Commun 12: 786 33542228 10.1038/s41467-020-20788-8 PMC7862263

[embj2023115030-bib-0029] Lam AKM , Rheinberger J , Paulino C , Dutzler R (2021) Gating the pore of the calcium‐activated chloride channel TMEM16A. Nat Commun 12: 785 33542223 10.1038/s41467-020-20787-9 PMC7862301

[embj2023115030-bib-0030] Lam AKM , Rutz S , Dutzler R (2022) Inhibition mechanism of the chloride channel TMEM16A by the pore blocker 1PBC. Nat Commun 13: 2798 35589730 10.1038/s41467-022-30479-1 PMC9120017

[embj2023115030-bib-0031] Lape R , Colquhoun D , Sivilotti LG (2008) On the nature of partial agonism in the nicotinic receptor superfamily. Nature 454: 722–727 18633353 10.1038/nature07139 PMC2629928

[embj2023115030-bib-0032] Le SC , Yang H (2020) An additional Ca^2+^ binding site allosterically controls TMEM16A activation. Cell Rep 33: 108570 33378669 10.1016/j.celrep.2020.108570 PMC7786149

[embj2023115030-bib-0033] Le SC , Jia Z , Chen J , Yang H (2019) Molecular basis of PIP2‐dependent regulation of the Ca^2+^‐activated chloride channel TMEM16A. Nat Commun 10: 3769 31434906 10.1038/s41467-019-11784-8 PMC6704070

[embj2023115030-bib-0034] Leffler JE (1953) Parameters for the description of transition states. Science 117: 340–341 17741025 10.1126/science.117.3039.340

[embj2023115030-bib-0035] Lim NK , Lam AK , Dutzler R (2016) Independent activation of ion conduction pores in the double‐barreled calcium‐activated chloride channel TMEM16A. J Gen Physiol 148: 375–392 27799318 10.1085/jgp.201611650 PMC5089934

[embj2023115030-bib-0036] Maity K , Heumann JM , McGrath AP , Kopcho NJ , Hsu PK , Lee CW , Mapes JH , Garza D , Krishnan S , Morgan GP *et al* (2019) Cryo‐EM structure of OSCA1.2 from *Oryza sativa* elucidates the mechanical basis of potential membrane hyperosmolality gating. Proc Natl Acad Sci USA 116: 14309–14318 31227607 10.1073/pnas.1900774116 PMC6628804

[embj2023115030-bib-0037] Mukhtasimova N , Lee WY , Wang HL , Sine SM (2009) Detection and trapping of intermediate states priming nicotinic receptor channel opening. Nature 459: 451–454 19339970 10.1038/nature07923 PMC2712348

[embj2023115030-bib-0038] Paulino C , Kalienkova V , Lam AKM , Neldner Y , Dutzler R (2017a) Activation mechanism of the calcium‐activated chloride channel TMEM16A revealed by cryo‐EM. Nature 552: 421–425 29236691 10.1038/nature24652

[embj2023115030-bib-0039] Paulino C , Neldner Y , Lam AK , Kalienkova V , Brunner JD , Schenck S , Dutzler R (2017b) Structural basis for anion conduction in the calcium‐activated chloride channel TMEM16A. Elife 6: e26232 28561733 10.7554/eLife.26232 PMC5470873

[embj2023115030-bib-0040] Peters CJ , Yu H , Tien J , Jan YN , Li M , Jan LY (2015) Four basic residues critical for the ion selectivity and pore blocker sensitivity of TMEM16A calcium‐activated chloride channels. Proc Natl Acad Sci USA 112: 3547–3552 25733897 10.1073/pnas.1502291112 PMC4371966

[embj2023115030-bib-0041] Peters CJ , Gilchrist JM , Tien J , Bethel NP , Qi LJ , Chen TX , Wang L , Jan YN , Grabe M , Jan LY (2018) The sixth transmembrane segment is a major gating component of the TMEM16A calcium‐activated chloride channel. Neuron 97: 1063–1077 29478917 10.1016/j.neuron.2018.01.048 PMC5860880

[embj2023115030-bib-0042] Pettersen EF , Goddard TD , Huang CC , Couch GS , Greenblatt DM , Meng EC , Ferrin TE (2004) UCSF Chimera—a visualization system for exploratory research and analysis. J Comput Chem 25: 1605–1612 15264254 10.1002/jcc.20084

[embj2023115030-bib-0043] Pettersen EF , Goddard TD , Huang CC , Meng EC , Couch GS , Croll TI , Morris JH , Ferrin TE (2021) UCSF ChimeraX: structure visualization for researchers, educators, and developers. Protein Sci 30: 70–82 32881101 10.1002/pro.3943 PMC7737788

[embj2023115030-bib-0044] Plested AJ (2016) Structural mechanisms of activation and desensitization in neurotransmitter‐gated ion channels. Nat Struct Mol Biol 23: 494–502 27273633 10.1038/nsmb.3214

[embj2023115030-bib-0045] Schroeder BC , Cheng T , Jan YN , Jan LY (2008) Expression cloning of TMEM16A as a calcium‐activated chloride channel subunit. Cell 134: 1019–1029 18805094 10.1016/j.cell.2008.09.003 PMC2651354

[embj2023115030-bib-0046] Sorum B , Czege D , Csanady L (2015) Timing of CFTR pore opening and structure of its transition state. Cell 163: 724–733 26496611 10.1016/j.cell.2015.09.052

[embj2023115030-bib-0047] Ta CM , Acheson KE , Rorsman NJG , Jongkind RC , Tammaro P (2017) Contrasting effects of phosphatidylinositol 4,5‐bisphosphate on cloned TMEM16A and TMEM16B channels. Br J Pharmacol 174: 2984–2999 28616863 10.1111/bph.13913 PMC5573538

[embj2023115030-bib-0048] Tan YZ , Baldwin PR , Davis JH , Williamson JR , Potter CS , Carragher B , Lyumkis D (2017) Addressing preferred specimen orientation in single‐particle cryo‐EM through tilting. Nat Methods 14: 793–796 28671674 10.1038/nmeth.4347 PMC5533649

[embj2023115030-bib-0049] Tembo M , Wozniak KL , Bainbridge RE , Carlson AE (2019) Phosphatidylinositol 4,5‐bisphosphate (PIP2) and Ca^2+^ are both required to open the Cl^−^ channel TMEM16A. J Biol Chem 294: 12556–12564 31266809 10.1074/jbc.RA118.007128 PMC6699839

[embj2023115030-bib-0050] Twomey EC , Sobolevsky AI (2018) Structural mechanisms of gating in ionotropic glutamate receptors. Biochemistry 57: 267–276 29037031 10.1021/acs.biochem.7b00891 PMC5780838

[embj2023115030-bib-0051] Wagner T , Merino F , Stabrin M , Moriya T , Antoni C , Apelbaum A , Hagel P , Sitsel O , Raisch T , Prumbaum D *et al* (2019) SPHIRE‐crYOLO is a fast and accurate fully automated particle picker for cryo‐EM. Commun Biol 2: 218 31240256 10.1038/s42003-019-0437-z PMC6584505

[embj2023115030-bib-0052] Williams CJ , Headd JJ , Moriarty NW , Prisant MG , Videau LL , Deis LN , Verma V , Keedy DA , Hintze BJ , Chen VB *et al* (2018) MolProbity: more and better reference data for improved all‐atom structure validation. Protein Sci 27: 293–315 29067766 10.1002/pro.3330 PMC5734394

[embj2023115030-bib-0053] Yang YD , Cho H , Koo JY , Tak MH , Cho Y , Shim WS , Park SP , Lee J , Lee B , Kim BM *et al* (2008) TMEM16A confers receptor‐activated calcium‐dependent chloride conductance. Nature 455: 1210–1215 18724360 10.1038/nature07313

[embj2023115030-bib-0054] Ye W , Han TW , He M , Jan YN , Jan LY (2019) Dynamic change of electrostatic field in TMEM16F permeation pathway shifts its ion selectivity. Elife 8: e45187 31318330 10.7554/eLife.45187 PMC6690719

[embj2023115030-bib-0055] Yu K , Jiang T , Cui Y , Tajkhorshid E , Hartzell HC (2019) A network of phosphatidylinositol 4,5‐bisphosphate binding sites regulates gating of the Ca^2+^‐activated Cl^−^ channel ANO1 (TMEM16A). Proc Natl Acad Sci USA 116: 19952–19962 31515451 10.1073/pnas.1904012116 PMC6778221

[embj2023115030-bib-0056] Yu J , Zhu H , Lape R , Greiner T , Du J , Lu W , Sivilotti L , Gouaux E (2021) Mechanism of gating and partial agonist action in the glycine receptor. Cell 184: 957–968 33567265 10.1016/j.cell.2021.01.026 PMC8115384

[embj2023115030-bib-0057] Zhang K (2016) Gctf: real‐time CTF determination and correction. J Struct Biol 193: 1–12 26592709 10.1016/j.jsb.2015.11.003 PMC4711343

[embj2023115030-bib-0058] Zhang M , Wang D , Kang Y , Wu JX , Yao F , Pan C , Yan Z , Song C , Chen L (2018) Structure of the mechanosensitive OSCA channels. Nat Struct Mol Biol 25: 850–858 30190597 10.1038/s41594-018-0117-6

[embj2023115030-bib-0059] Zheng L , Baumann U , Reymond JL (2004) An efficient one‐step site‐directed and site‐saturation mutagenesis protocol. Nucleic Acids Res 32: e115 15304544 10.1093/nar/gnh110 PMC514394

[embj2023115030-bib-0060] Zheng SQ , Palovcak E , Armache JP , Verba KA , Cheng Y , Agard DA (2017) MotionCor2: anisotropic correction of beam‐induced motion for improved cryo‐electron microscopy. Nat Methods 14: 331–332 28250466 10.1038/nmeth.4193 PMC5494038

[embj2023115030-bib-0061] Zivanov J , Nakane T , Forsberg BO , Kimanius D , Hagen WJ , Lindahl E , Scheres SH (2018) New tools for automated high‐resolution cryo‐EM structure determination in RELION‐3. Elife 7: e42166 30412051 10.7554/eLife.42166 PMC6250425

