# Supplementary material for: Mechanistic basis of ligand efficacy in the calcium‐activated chloride channel TMEM16A
Source: EMBO J. 2023 Nov 20;42(24):e115030. doi: 10.15252/embj.2023115030 (PMC10711664; doi:10.15252/embj.2023115030)
Supplement: Supplementary file 1 — Appendix [file EMBJ-42-e115030-s002.pdf]

# Appendix

## Mechanistic basis of ligand efficacy in the calcium-activated chloride channel TMEM16A

Andy K. M. Lam and Raimund Dutzler

**Includes:**

Appendix Supplementary Methods

Appendix Figure S1-3

Appendix Table S1-4

**Table of contents**


| | Page |
|---|---|
| Appendix Supplementary Methods | |
| Analysis of current-voltage (I-V) relations | 3 |
| Non-stationary noise analysis | 4 |
| Autocorrelation analysis | 5 |
| Double-mutant cycle analysis | 7 |
| Rate-equilibrium free-energy relation analysis | 8 |
| Statistics | 8 |
| Appendix Supplementary Figures | |
| Appendix Figure S1 | 10 |
| Appendix Figure S2 | 12 |
| Appendix Figure S3 | 13 |
| Appendix Supplementary Tables | |
| Appendix Table S1 | 15 |
| Appendix Table S2 | 15 |
| Appendix Table S3 | 15 |
| Appendix Table S4 | 16 |

**Appendix Supplementary Methods**

**Analysis of current-voltage (I-V) relations**

I-V data were fitted to a minimal permeation model that accounts for the fundamental biophysical behavior of mouse TMEM16A as described previously (Paulino *et al.*, 2017b),

$$I = zFAe^{\frac{zFV}{2nRT}} \frac{c_i - c_o e^{-\frac{zFV}{RT}}}{e^{-zFV\frac{n-1}{nRT}} + \left(\frac{1}{\sigma_h}\right)\frac{1 - e^{-zFV\frac{n-2}{nRT}}}{e^{\frac{zFV}{nRT}} - 1} + \frac{1}{\sigma_\beta}} \qquad [1]$$

where $I$ is the current, $n$ is the number of barriers, $c_i$ and $c_o$ are the intracellular and extracellular concentrations of the charge carrier, $z$ is the valence of $Cl^-$, $V$ is the membrane voltage, and $R$, $T$, and $F$ have their usual thermodynamic meanings. $A = \beta_0 v$ is a proportionality factor where $\beta_0$ is the value of $\beta$ when $V = 0$ and $v$ is a proportionality coefficient that has a dimension of volume. $\sigma_h$ and $\sigma_\beta$ are respectively the rate of barrier crossing at the middle and the innermost barriers relative to that at the outermost barrier ($\beta$). The best-fit values of $\sigma_\beta$ and $\sigma_h$ at a saturating $Ca^{2+}$ concentration were used to calculate $\Delta E_{a\,(\sigma_\beta)}$ and $\Delta E_{a\,(\sigma_h)}$, the difference between the activation energy at the innermost barrier and the middle barrier relative to that of the outermost respectively, using

$$\Delta E_{a\,(\sigma_\beta)} = -RT\ln\sigma_\beta$$
$$\Delta E_{a\,(\sigma_h)} = -RT\ln\sigma_h \qquad [2]$$

When analyzed on a unitary scale,

$$\beta_0/\beta_{0\mathrm{WT}} = \mathrm{A}/\mathrm{A}_{\mathrm{WT}}$$
$$\Delta E_{a\,(\beta)} = -RT\ln\frac{\beta_0}{\beta_{0\mathrm{WT}}} \qquad [3]$$

were used to estimate the elevation of the outer barrier.

The half-maximum inhibition concentrations ($IC_{50}$) of 1PBC at the indicated voltages were obtained from steady-state I-V relations at a saturating $Ca^{2+}$ concentration by fitting the concentration-response

relations to the Hill equation. The apparent valence of 1PBC ($z_{\text{block}}$) was estimated by fitting the voltage dependence of the $IC_{50}$ in the exponential range (±40 mV) using

$$\mathrm{IC}_{50} = \mathrm{IC}_{50(0)} e^{\delta_b z_b VF/RT}$$

$$z_{\text{block}} = \delta_b z_b \qquad [4]$$

where $\mathrm{IC}_{50(0)}$ is the $IC_{50}$ in the absence of voltage, $\delta_b$ is the fraction of the transmembrane electric field operating on the blocker at its binding site, and $z_b$ is the valence of the blocker.

**Non-stationary noise analysis**

Variance-current parabolas were calculated and analyzed as described previously (Lam & Dutzler, 2021). The current and variance were sampled by repeatedly activating and deactivating the channel using regularly spaced concentration jumps. The variance of such 50–100 aligned successive and kinetically identical currents at each time point was calculated by computing the mean of the squared successive difference (Heinemann & Conti, 1992), which mitigates the effect of non-stationarity at each isochrone and therefore allows the estimation of the variance in the presence of current rundown. The data were fitted to

$$\sigma_{\text{total}}^2 = \sigma_N^2 + \sigma_{\text{bg}}^2$$

$$\sigma_N^2 = i(\bar{I} - \bar{I}_{\text{bg}}) - \frac{(\bar{I} - \bar{I}_{\text{bg}})^2}{N} \qquad [5]$$

where $\sigma_N^2$ is the variance for $N$ channels, $i$ is the unitary current, $\bar{I}$ is the mean current, and the subscript $bg$ denotes background. Data from different patches were merged by normalizing both x and y data according to the patch-specific parameter $iN$, the maximum achievable $\bar{I}$ for each patch if the Po was 1. Each data pair ($\bar{I}_j, \sigma_{N\,j}^2$) was sorted according to the $\bar{I}$ values and were averaged using a Gaussian moving average filter. The averaged data were re-fitted to Eq. 5 without the $\sigma_{bg}^2$ and $\bar{I}_{bg}$ terms. This procedure allows the estimated Po to be directly read from the merged $\sigma_N^2$-$\bar{I}$ plots.

**Autocorrelation analysis**

Power spectra were calculated and analyzed as described previously (Lam & Dutzler, 2021). Time series of steady-state currents of 50 or 100 s, recorded at a saturating $Ca^{2+}$ concentration at +80 mV, were processed via Fast Fourier Transform (FFT) with a Hamming window applied to mitigate edge discontinuities. The data were digitized at a 20 kHz after passing through a 4-pole Bessel filter at 10 kHz. The background spectrum, recorded at 0 mV where the current reverses, was subtracted from the raw spectrum. The resulting spectrum was fitted to an empirical function consisting of a 1/f-like component, Lorentzian components, and a constant term,

$$P = \frac{a_0}{f^n} + \sum_i a_i \frac{1}{1 + \left(f/f_{c_i}\right)^2} + c \qquad [6]$$

where $f$ is frequency, $n$ is an exponent describing the decay, $a_0$ and $a_i$ are respectively the amplitude of the 1/f-like and the Lorentzian components, $f_{c_i}$ is the corner frequency, and $c$ is a constant. A linear combination of three Lorentzian components was used, and the 1/f-like and the constant terms were subtracted from the background-subtracted spectrum, yielding a spectrum corresponding to fluctuations that reflect gating transitions.

The final spectrum was fitted to the following mechanism,

$$C_3 \leftrightarrow C_2 \leftrightarrow C_1 \leftrightarrow O_0$$

where C and O correspond to closed and open states respectively, and the subscripts denote the number assigned to the states. The matrix notation of this mechanism (Colquohoun & Hawkes, 1995) is

$$\boldsymbol{Q} = \begin{bmatrix} -k_{01} & k_{01} & 0 & 0 \\ k_{10} & -k_{10} - k_{12} & k_{12} & 0 \\ 0 & k_{21} & -k_{21} - k_{23} & k_{23} \\ 0 & 0 & k_{32} & -k_{32} \end{bmatrix}$$

where the subscripts indicate the transition described by the rate constant $k$ in $s^{-1}$, for example $k_{01}$ corresponds to the rate constant of the transition from state 0 to 1. $k_{10}$ was calculated using

$$k_{10} = k_{01}\left(\frac{P_O}{1 - P_O}\right)\left(\frac{1 + L_{32} + L_{32}L_{21}}{L_{32}L_{21}}\right)$$

where $L$ is the forward equilibrium constant with the subscript indicating the transition, and $P_O$ was obtained from non-stationary noise analysis performed at a saturating $Ca^{2+}$ concentration ($Po_{\max}$).

The equilibrium occupancy of states was calculated from (Colquohoun & Hawkes, 1995)

$$\boldsymbol{P}(\infty) = \boldsymbol{Y_0}(\boldsymbol{V}_{\lambda=0}\boldsymbol{V}^{-1}{}_{\lambda=0}) \qquad [7]$$

where $\boldsymbol{Y_0}$ is the initial occupancy and $\boldsymbol{V}$ can be obtained from the Eigen decomposition of $\boldsymbol{Q}$

$$\boldsymbol{Q} = \boldsymbol{V\Lambda V}^{-1}$$

and

$$\boldsymbol{\Lambda} = \begin{bmatrix} \lambda_1 & & \\ & \ddots & \\ & & \lambda_n \end{bmatrix}$$

$$\boldsymbol{V} = \begin{bmatrix} v_{11} & \cdots & v_{n1} \\ \vdots & \ddots & \vdots \\ v_{1n} & \cdots & v_{nn} \end{bmatrix}$$

are the Eigenvalue and Eigenvector matrices respectively. The corresponding spectral matrices are given by

$$\boldsymbol{A_i} = \boldsymbol{V}_{i^{th}col}\boldsymbol{V}^{-1}{}_{i^{th}row}$$

The data were fitted to the single-sided power spectrum due to Markovian fluctuations (Colquhoun & Hawkes, 1977)

$$G(f) = 4NV^2\boldsymbol{P_o}(\infty)\boldsymbol{\Gamma_o}\left[\sum_{i=2}^{n}\boldsymbol{A_{i_{oo}}}\frac{-\lambda_i^{-1}}{1+\left(\frac{2\pi f}{\lambda_i}\right)^2}\right]\boldsymbol{\Gamma_o u_o} \qquad [8]$$

where $N$ is the number of conducting units, $V$ is the membrane potential,

$$\boldsymbol{P_o}(\infty) = \boldsymbol{P}(\infty)_{(o_1 \ldots o_k)}$$

is the steady-state occupancy of open states 1 to k,

$$\boldsymbol{A_{i_{oo}}} = \boldsymbol{A_{i}}_{(o_1 \ldots o_k,\, o_1 \ldots o_k)}$$

is a submatrix of the spectral matrix and $o_1 \ldots o_k$, $o_1 \ldots o_k$ denote the upper left elements,

$$\boldsymbol{\Gamma_o} = \begin{bmatrix} \gamma_{o_1} & & \\ & \ddots & \\ & & \gamma_{o_k} \end{bmatrix}$$

is the conductance of the states arranged in a matrix form, and

$$\boldsymbol{u_o} = \begin{bmatrix} 1 \\ \vdots \\ 1 \end{bmatrix}$$

is a unit vector of length corresponding to the number of open states. Because the amplitude of the power spectrum concerns the number of channels and their conductance, which are variables not related to mechanism, we fitted the experimental power spectra using a normalized form

$$G_{norm}(f) = \frac{G(f)}{G(0)} \qquad [9]$$

where $G(0)$ is a constant corresponding to the power at very low frequency. The parameters were estimated by minimizing the sum of squares.

The uniqueness in of the estimated parameters was evaluated by calculating the sum-of-square errors using the experimental spectrum as a function of each directly fitted rate constant with the other parameters fixed at their fitted values. The correlation between selected rate constants was evaluated by calculating the error surface on a two-dimensional grid with the remaining parameters fixed at their fitted values.

**Double-mutant cycle analysis**

The free energy of transition ($\Delta G$) was calculated from the forward equilibrium constant using

$$\Delta G_{ij} = -RT \ln L_{ij}$$

where $R$ and $T$ have their usual thermodynamic meanings, $L$ is the forward equilibrium constant and the subscript indicates the transition from state $i$ to $j$. A double-mutant cycle (Carter *et al*, 1984; Horovitz, 1996) can be described by the following scheme

$$
\begin{array}{ccccc}
 & & \Delta\Delta G_{ij}^{(0-X,Y)} & & \\
 & X,Y & \rightarrow & 0,Y & \\
\Delta\Delta G_{ij}^{(X,0-Y)} & \downarrow & & \downarrow & \Delta\Delta G_{ij}^{(0,0-Y)} \\
 & X,0 & \rightarrow & 0,0 & \\
 & & \Delta\Delta G_{ij}^{(0-X,0)} & &
\end{array}
$$

where $X$ and $Y$ are two residues of interest and 0 denotes a mutation. The coupling energy between $X$ and $Y$ ($\Delta\Delta\Delta G^{XY}$) was calculated using either the $X$ or $Y$ mutations

$$\Delta\Delta\Delta G_{ij}^{XY} = \Delta\Delta G_{ij}^{(0-X,0)} - \Delta\Delta G_{ij}^{(0-X,Y)} = \left(\Delta G_{ij}^{(0,0)} - \Delta G_{ij}^{(X,0)}\right) - \left(\Delta G_{ij}^{(0,Y)} - \Delta G_{ij}^{(X,Y)}\right) \quad [10]$$

The standard error ($\sigma$) of the parameter estimates for each subtraction was propagated as described in the Statistics section. Deviation of $\Delta\Delta\Delta G_{ij}^{XY}$ from zero was detected using a one-sample t-test with a significance level of 0.05.

**Rate-equilibrium free-energy relation analysis**

The rate-equilibrium free-energy relation (Leffler, 1953; Leffler & Grunwald, 1963) consists of the following pair of relations

$$
\begin{aligned}
\log k_f &= \log k_i + \phi \log L \\
\log k_b &= \log k_i + (\phi - 1) \log L
\end{aligned}
\quad [11]
$$

that describe the effect of a series of perturbations on the rate constants ($k_f$ and $k_b$) as a fraction ($\phi$ and $\phi - 1$) of their effect on the forward equilibrium constant $L$. $\phi$ can adopt values between 0 and 1. $k_i$ is the rate constant when $L$=1. The parameters $\phi$ and $k_i$ were estimated by minimizing the total sum of squares for the set of equations for each transition.

**Statistics**

Data analysis was performed using Clampfit 10.7 (Molecular Devices), Excel (Microsoft), NumPy (https://numpy.org), and SciPy (https://scipy.org). For numerical calculations, NumPy and SciPy were

used. Parameter optimization was performed by minimizing the sum of squares using the least_squares function in SciPy. The variance of the best-fit parameters was obtained from the diagonal elements of the variance-covariance matrix (Colquhoun *et al*, 2003; van de Geer, 2005)

$$\boldsymbol{H}^{-1} = (\boldsymbol{J}^T \cdot \boldsymbol{J})^{-1}$$

multiplied by

$$\sigma^2 = \frac{\epsilon(\theta_{\text{est}})}{n_d - n_p}$$

where $\boldsymbol{H}$ and $\boldsymbol{J}$ are the Hessian and Jacobian matrices at the least squares estimates respectively, the superscript $T$ indicates transpose, $\epsilon_G(\theta_{\text{est}})$ is the sum of squares given the estimated parameters, and $n_d$ and $n_p$ are the number of data points and parameters respectively. The square root of the variance was used to approximate the standard deviation error, from which the 95% confidence interval was calculated. Experimental data consisting of individual measurements are presented as mean ± SEM. Estimated parameters are presented as best-fit ± 95% confidence interval unless otherwise stated. Uncertainties were propagated using

$$\sigma_{(a+b\ or\ a-b)} = \sqrt{{\sigma_a}^2 + {\sigma_b}^2}$$

$$\frac{\sigma_{(ab\ or\ a/b)}}{|f(a,b)|} = \sqrt{\left(\frac{\sigma_a}{|a|}\right)^2 + \left(\frac{\sigma_b}{|b|}\right)^2}$$

The t-test, with a significance level of 0.05, was used for statistical comparison. No statistical methods were used to estimate the sample size. No blinding was performed.

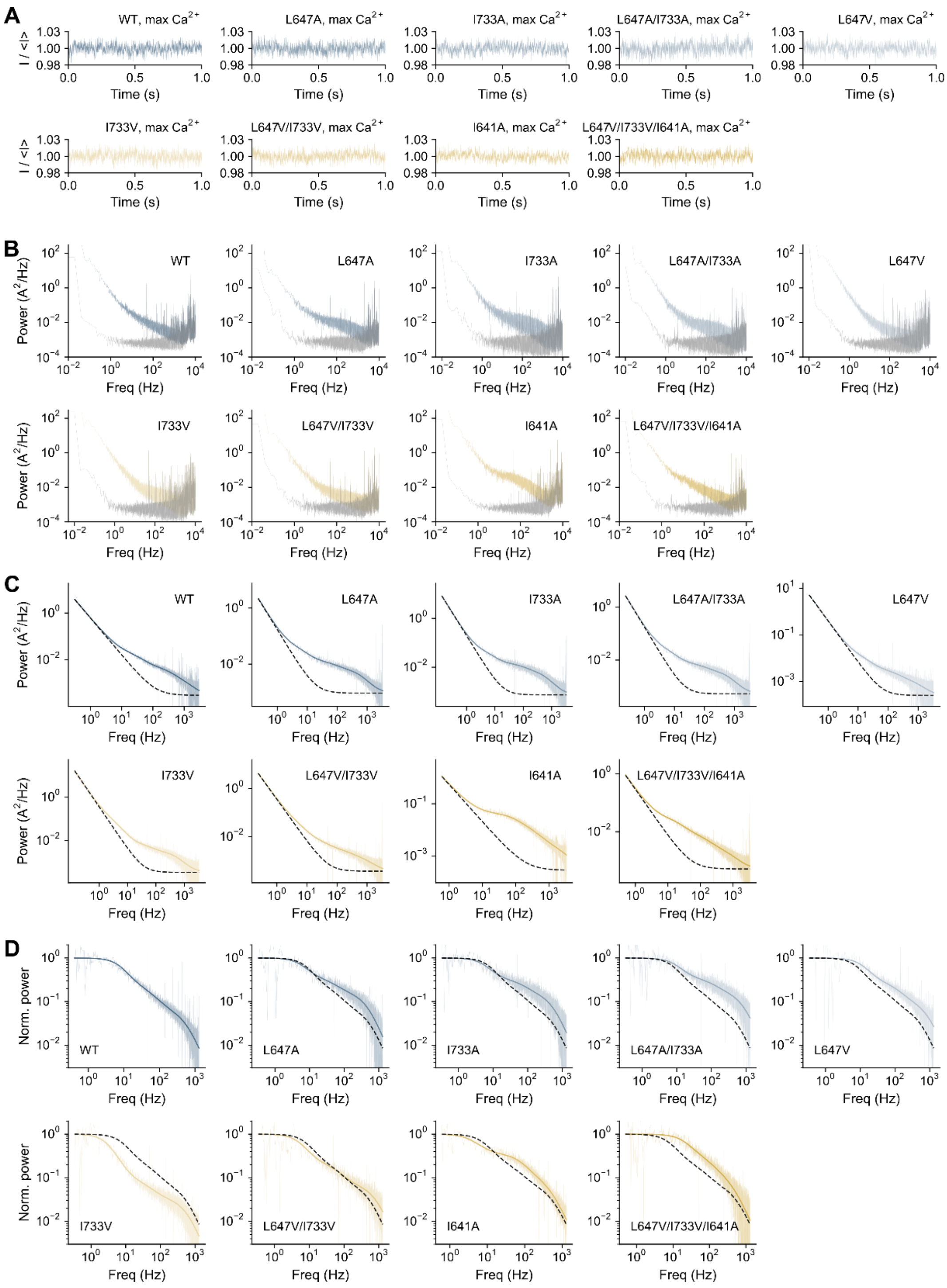


## Appendix Figure S1. Autocorrelation analysis.

A. Representative section of steady-state current at a saturating $Ca^{2+}$ concentration for the indicated mutants. For display, the traces were filtered at 2 kHz using a digital 4-pole Bessel lowpass filter.

B. Raw power spectra calculated from the steady-state currents shown in (A) and the corresponding background recorded at the reversal potential of $Cl^-$ (grey).

C. Background-subtracted power spectra. Solid line is an empirical fit to Eq. 6 comprising the 1/f and constant components and components corresponding to fluctuations associated with channel gating. Dashed lines correspond to the estimated 1/f and constant components that are subtracted from the total spectrum to yield the gating component.

D. Normalized power spectra reflecting fluctuations due to channel gating. Solid line is a fit to Eqs. 7-9. Dashed line corresponds to the wild-type spectrum.

B-D. Data are averages of the indicated number of patches (WT, n=7; L647A, n=5; I733A, n=6; L647A/I733A, n=7; L647V, n=10; I733V, n=8; L647V/I733V, n=5; I641A, n=7; L647V/I733V/I641A, n=6).

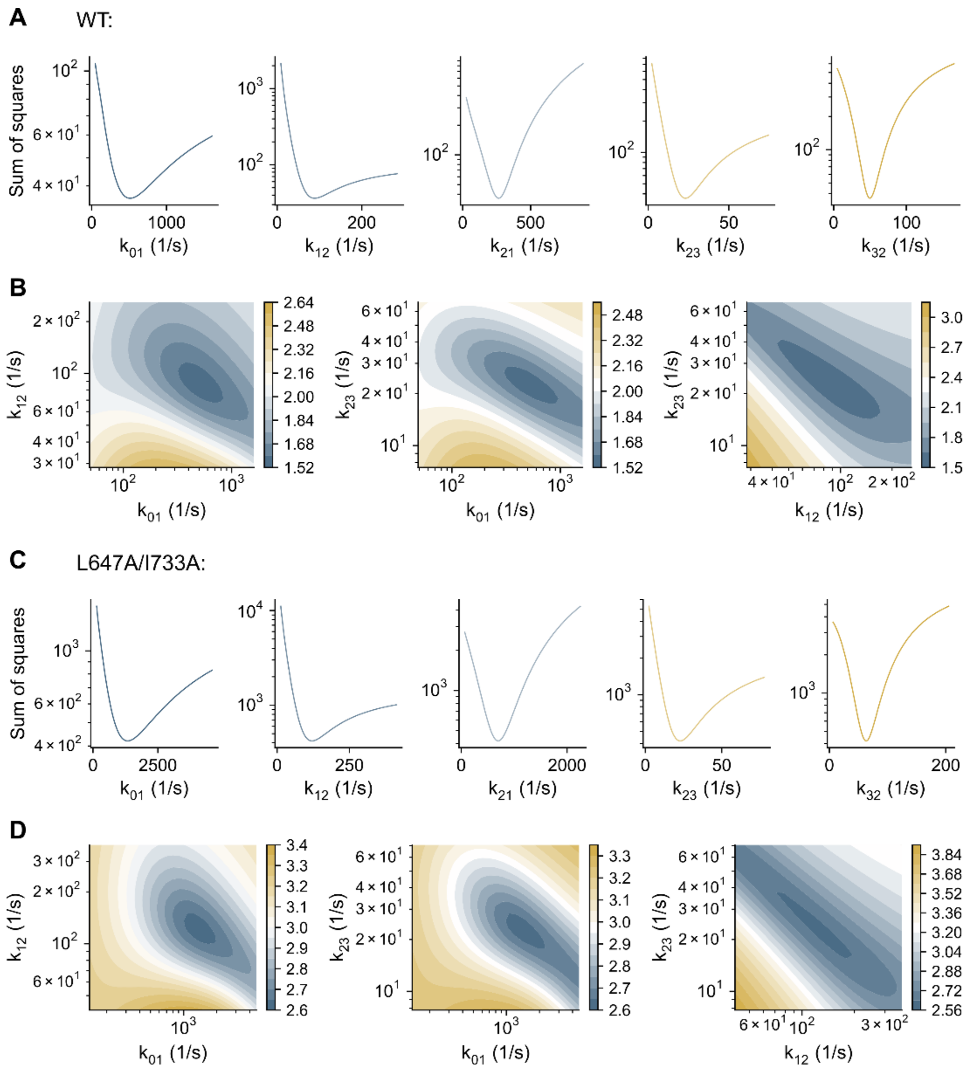


**Appendix Figure S2. Estimatability of parameters from autocorrelation analysis.**

A and C. Error function of the directly fitted rate constants of wild-type (A) and L647A/I733A (C). The errors were calculated using the corresponding experimental spectra with the other parameters fixed at their fitted values. A well-defined minimum is observed for each of the estimated parameters within the range consistent with the experimental characteristic frequencies.

B and D. Error surface of the backward rate constants of wild-type (B) and L647A/I733A (D). Plotted are the sum-of-squares errors calculated using the corresponding experimental spectra with the remaining three parameters fixed at their fitted values.

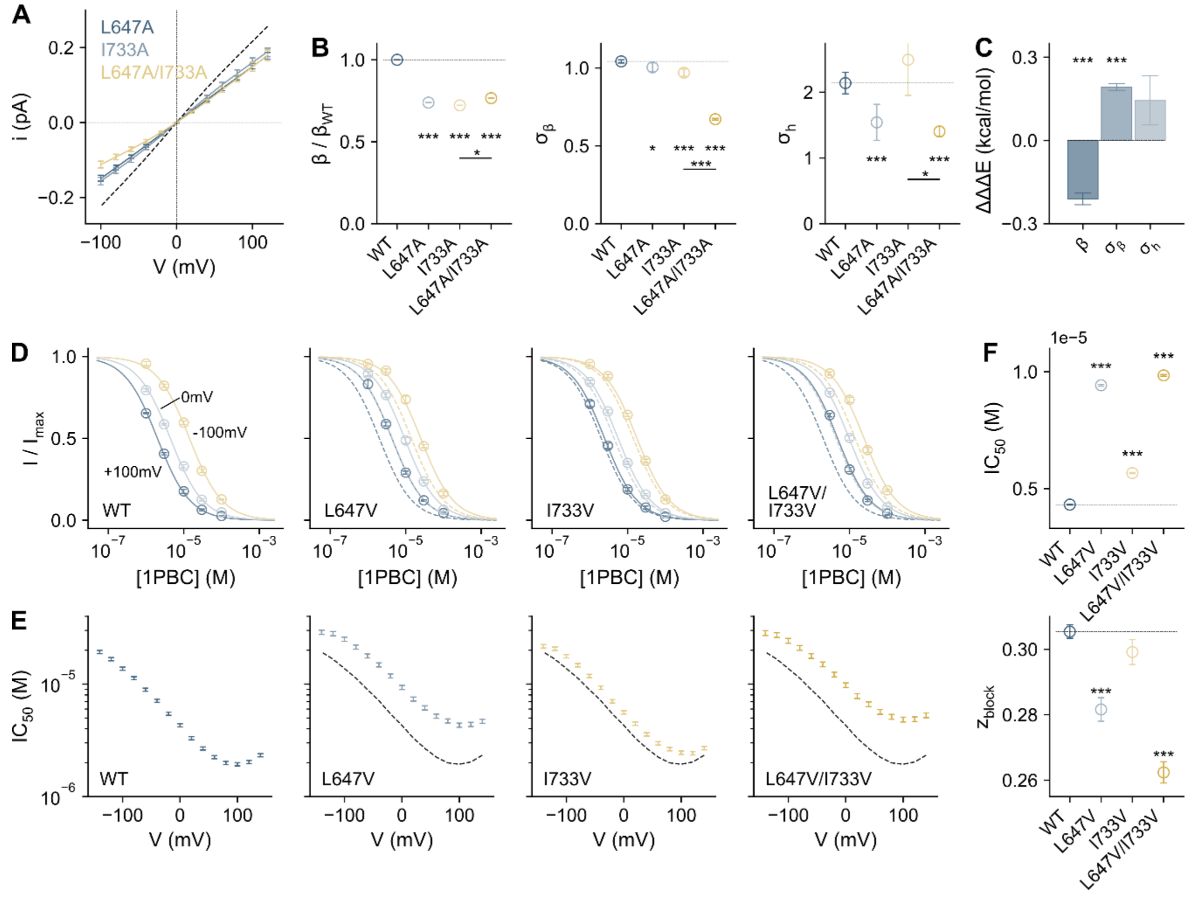

**Appendix Figure S3. Pore properties of mutants.**

A. Instantaneous current-voltage (I-V) relations of the indicated mutants at a saturating $Ca^{2+}$ concentration on a unitary scale. Data were scaled according to the estimated unitary current from non-stationary noise analysis at +80 mV (Fig 1, Appendix Fig S1, and Appendix Table S2). Data are averages of 6, 7, 4, and 4 patches for WT, L647A, I733A, and L647A/I733A respectively, and errors are SEM. Solid lines are fits to a model of ion permeation (Eq. 1) shown in Fig 3B. Dashed line is the relation of wild-type.

B. Conduction parameters for the indicated barriers. Data are best-fit values, and errors are 95% confidence intervals. Dashed line indicates the value of wild-type. t-test: *, $p < 0.05$; ***, $p < 0.005$

C. Coupling energies (ΔΔΔE) for the indicated barriers. Bars indicate quantities calculated using Eqs. 2, 3, and 10, and errors are standard errors. t-test: ***, $p < 0.005$

D. Concentration-response relations of 1PBC at -100, 0, and 100 mV of the indicated mutants. Data are averages of 6, 6, 6, and 7 patches for WT, L647V, I733V, and L647V/I733V respectively, and errors are SEM. Solid lines are fits to the Hill equation. Dashed lines are the relations of wild-type.

E. Inhibition by 1PBC as a function of voltage for the indicated mutants. Data are best-fit values from a fit to the Hill equation using averaged concentration-response curves at the indicated voltages from the data shown in (D), and errors are 95% confidence intervals. Dashed line is the relation of wild-type.

F. $IC_{50}$ at zero mV and $z_{block}$ of the indicated constructs estimated by fitting the data in (E) using Eq. 4. Data are best-fit values, and errors are standard errors. t-test: ***, $p < 0.005$

### Appendix Table S1. Concentration-response parameters at +80 mV

| | $EC_{50}$ (M) | SE | p-value | $n_H$ | SE | p-value | No. of patches |
|---|---|---|---|---|---|---|---|
| **WT** | 2.77e-07 | 1.83e-08 | - | 1.95 | 0.22 | - | 8 |
| **L647V** | 7.5E-07 | 4.91E-08 | *** | 1.76 | 0.17 | n.s. | 7 |
| **I733V** | 4.51E-07 | 1.92E-08 | *** | 1.71 | 0.11 | n.s. | 8 |
| **L647V/I733V** | 1.18E-06 | 4.25E-08 | *** | 1.78 | 0.09 | n.s. | 8 |
| **L647A** | 1.2E-06 | 5.49E-08 | *** | 1.46 | 0.09 | n.s | 8 |
| **I733A** | 1.12E-06 | 5.33E-08 | *** | 1.63 | 0.10 | n.s. | 8 |
| **L647A/I733A** | 1.8E-06 | 4.43E-08 | *** | 1.25 | 0.03 | * | 5 |

SE, standard error; n.s., non-significant; *, $p < 0.05$; ***, $p < 0.005$

### Appendix Table S2. Noise analysis parameters at +80 mV

| | $[Ca^{2+}]$ | $Po_{max}$ | SE | p-value | i (pA) | SE | p-value | No. of patches |
|---|---|---|---|---|---|---|---|---|
| **WT** | 4 µM | 0.775 | 0.027 | - | 0.18 | 0.017 | - | 11 |
| **L647V** | 15 µM | 0.771 | 0.019 | n.s. | 0.107 | 0.009 | *** | 8 |
| **I733V** | 4 µM | 0.762 | 0.013 | n.s. | 0.172 | 0.016 | n.s. | 10 |
| **L647V/I733V** | 15 µM | 0.663 | 0.029 | ** | 0.07 | 0.003 | *** | 20 |
| **L647A** | 15 µM | 0.684 | 0.021 | * | 0.12 | 0.009 | ** | 12 |
| **I733A** | 15 µM | 0.659 | 0.022 | *** | 0.132 | 0.011 | * | 10 |
| **L647A/I733A** | 50 µM | 0.657 | 0.035 | * | 0.118 | 0.014 | * | 10 |

SE, standard error; n.s., non-significant; *, $p < 0.05$; **, $p < 0.01$; ***, $p < 0.005$

### Appendix Table S3. Fitted gating parameters at saturating $Ca^{2+}$ at +80 mV

| | **WT** | | **L647A** | | **I733A** | | **L647A/I733A** | | **L647V** | |
|---|---|---|---|---|---|---|---|---|---|---|
| $[Ca^{2+}]$ | 4 µM | | 15 µM | | 15 µM | | 50 µM | | 15 µM | |
| | Best-fit | 95% CI | Best-fit | 95% CI | Best-fit | 95% CI | Best-fit | 95% CI | Best-fit | 95% CI |
| $k_{01}$ ($s^{-1}$) | 486.9 | 7.526 | 715.05 | 7.822 | 854.36 | 5.822 | 1398.4 | 17.406 | 846.5 | 32.564 |
| $k_{10}$ ($s^{-1}$) | 2593.6 | 221.10 | 2005.3 | 270.66 | 2258.7 | 159.58 | 3365.6 | 378.96 | 4052.6 | 836.07 |
| $k_{12}$ ($s^{-1}$) | 85.65 | 3.307 | 55.04 | 3.743 | 87.88 | 3.535 | 129.98 | 8.275 | 193.43 | 23.601 |
| $k_{21}$ ($s^{-1}$) | 267.31 | 8.842 | 328.35 | 16.68 | 474.07 | 11.913 | 704.76 | 28.311 | 740.96 | 58.7 |
| $k_{23}$ ($s^{-1}$) | 22.63 | 0.973 | 18.15 | 1.004 | 15.14 | 0.379 | 24.89 | 0.997 | 40 | 2.788 |
| $k_{32}$ ($s^{-1}$) | 50.25 | 0.856 | 41.61 | 1.079 | 39.16 | 0.468 | 64.77 | 1.243 | 83.45 | 2.551 |
| $L_{10}$ | 5.33 | 0.462 | 2.8 | 0.38 | 2.64 | 0.188 | 2.41 | 0.273 | 4.79 | 1.005 |
| $L_{21}$ | 3.12 | 0.159 | 5.97 | 0.506 | 5.39 | 0.256 | 5.42 | 0.408 | 3.83 | 0.556 |
| $L_{32}$ | 2.22 | 0.103 | 2.29 | 0.14 | 2.59 | 0.072 | 2.6 | 0.116 | 2.09 | 0.159 |

| | **I733V** | | **L647V/I733V** | | **I641A** | | **L647V/I733V/ I641A** | |
|---|---|---|---|---|---|---|---|---|
| $[Ca^{2+}]$ | 4 µM | | 15 µM | | 2 µM | | 15 µM | |
| | Best-fit | 95% CI | Best-fit | 95% CI | Best-fit | 95% CI | Best-fit | 95% CI |
| $k_{01}$ ($s^{-1}$) | 635.89 | 8.898 | 1320.0 | 44.532 | 219.28 | 2.403 | 203.98 | 6.344 |
| $k_{10}$ ($s^{-1}$) | 2669.1 | 250.76 | 3445.0 | 557.03 | 2552.3 | 137.06 | 2171.3 | 689.16 |
| $k_{12}$ ($s^{-1}$) | 51.98 | 2.959 | 101.7 | 9.106 | 199.15 | 6.024 | 234.89 | 44.638 |
| $k_{21}$ ($s^{-1}$) | 299.17 | 12.842 | 407.25 | 25.616 | 551.5 | 7.617 | 690.47 | 102.95 |
| $k_{23}$ ($s^{-1}$) | 26.21 | 0.607 | 18.01 | 1.069 | 4.53 | 0.105 | 108.72 | 11.579 |
| $k_{32}$ ($s^{-1}$) | 21.74 | 0.149 | 36.35 | 1.014 | 27.23 | 0.361 | 171.85 | 5.302 |
| $L_{10}$ | 4.2 | 0.399 | 2.61 | 0.431 | 11.64 | 0.638 | 10.65 | 3.395 |
| $L_{21}$ | 5.76 | 0.41 | 4 | 0.438 | 2.77 | 0.092 | 2.94 | 0.707 |

| | | | | | | | | |
|---|---|---|---|---|---|---|---|---|
| **$L_{32}$** | 0.83 | 0.02 | 2.02 | 0.132 | 6.01 | 0.16 | 1.58 | 0.175 |

Normalized power spectra were fitted; CI, confidence interval.

## Appendix Table S4. Fitted conduction parameters at saturating $Ca^{2+}$

| | **A** | | **$\sigma_\beta$** | | **$\sigma_h$** | |
|---|---|---|---|---|---|---|
| | Best-fit | 95% CI | Best-fit | 95% CI | Best-fit | 95% CI |
| **WT** | 0.12 | 0.0007 | 1.042 | 0.0038 | 2.139 | 0.0592 |
| **L647V** | 0.108 | 0.0011 | 0.549 | 0.0034 | 0.727 | 0.0193 |
| **I733V** | 0.12 | 0.0008 | 0.965 | 0.0039 | 1.868 | 0.0548 |
| **L647V/I733V** | 0.11 | 0.001 | 0.308 | 0.002 | 0.343 | 0.0057 |
| **L647A** | 0.088 | 0.0013 | 1.004 | 0.0097 | 1.541 | 0.0913 |
| **I733A** | 0.086 | 0.0015 | 0.97 | 0.0105 | 2.494 | 0.2423 |
| **L647A/I733A** | 0.092 | 0.0006 | 0.671 | 0.0025 | 1.403 | 0.0334 |

Instantaneous currents on the absolute scale were fitted; CI, confidence interval.
