# Supplementary material for: Mechanistic basis of ligand efficacy in the calcium‐activated chloride channel TMEM16A
Source: EMBO J. 2023 Nov 20;42(24):e115030. doi: 10.15252/embj.2023115030 (PMC10711664; doi:10.15252/embj.2023115030)
Supplement: Supplementary file 2 — Expanded View Figures PDF [file EMBJ-42-e115030-s005.pdf]

Expanded View Figures

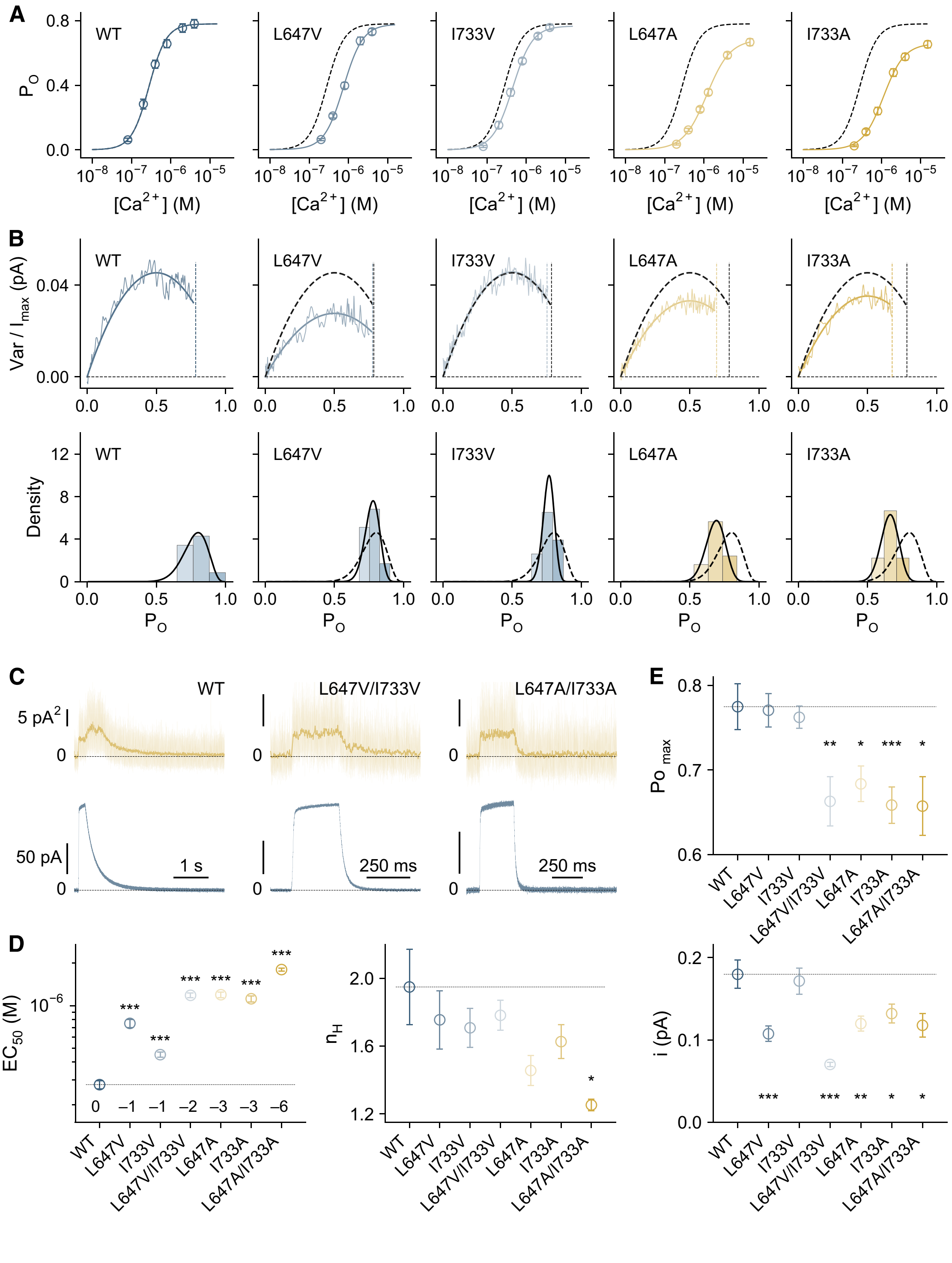

Figure EV1.

16 of 15 *The EMBO Journal* e115030 | 2023 © 2023 The Authors

Figure EV1. Activation properties of mutants.

A Concentration-Po relations for the indicated mutants at +80 mV. Data are averages of 8, 7, 8, 8, and 8 patches for WT, L647V, I733V, L647A, and I733A respectively, and errors are SEM. Solid line is a fit to the Hill equation. Dashed line is the relation of wild-type.

B Top, merged and averaged variance-current relations at a saturating Ca^{2+} concentration at +80 mV. Data are averages of 11, 8, 10, 12, and 10 patches for WT, L647V, I733V, L647A, and I733A respectively. Solid line is a fit to Equation 5 in Supplementary Methods. Dashed line is the relation of wild-type. Dotted lines indicate the maximum Po. Bottom, histograms of the maximum Po obtained from individual measurements. Solid line is a fit to the beta distribution. Dashed line is the distribution of wild-type.

C Representative mean current and variance upon a step-exchange from zero to saturating Ca^{2+} and back. The raw variance is overlaid with its Gaussian moving average. Dashed lines indicate the zero current/variance levels.

D EC_{50} and n_H of the indicated constructs. Data are averages of the indicated number of patches shown in Appendix Table S1, and errors are SEM. The number of methyl groups truncated relative to wild-type is indicated in the left panel. WT, $n = 8$; L647V, $n = 7$; I733V, $n = 8$; L647V/I733V, $n = 8$; L647A, $n = 8$; I733A, $n = 8$; L647A/I733A, $n = 5$. *t*-test: $*P < 0.05$; $***P < 0.005$.

E Po_{max} and i of the indicated constructs. Data are averages of the indicated number of patches shown in Appendix Table S2, and errors are SEM. WT, $n = 11$; L647V, $n = 8$; I733V, $n = 10$; L647V/I733V, $n = 20$; L647A, $n = 12$; I733A, $n = 10$; L647A/I733A, $n = 10$. *t*-test: $*P < 0.05$; $**P < 0.01$; $***P < 0.005$.

Figure EV2. Cryo-EM reconstruction of Ca^{2+}-bound L647V/I733V.

A Representative micrographs (scale bar: 50 nm) and 2D class averages of L647V/I733V in the presence of Ca^{2+} for the indicated samples.

B Data processing workflow.

C Local resolution of the final map estimated using RELION.

D Angular distribution of particle projections used in the final refinement. Scale bar indicates the number of particle images.

E Half-map FSCs.

F Model-map FSCs.

G, H Sections of cryo-EM densities of (G) selected transmembrane helices and (H) the principal Ca^{2+}-binding site superimposed on the refined model.

© 2023 The Authors

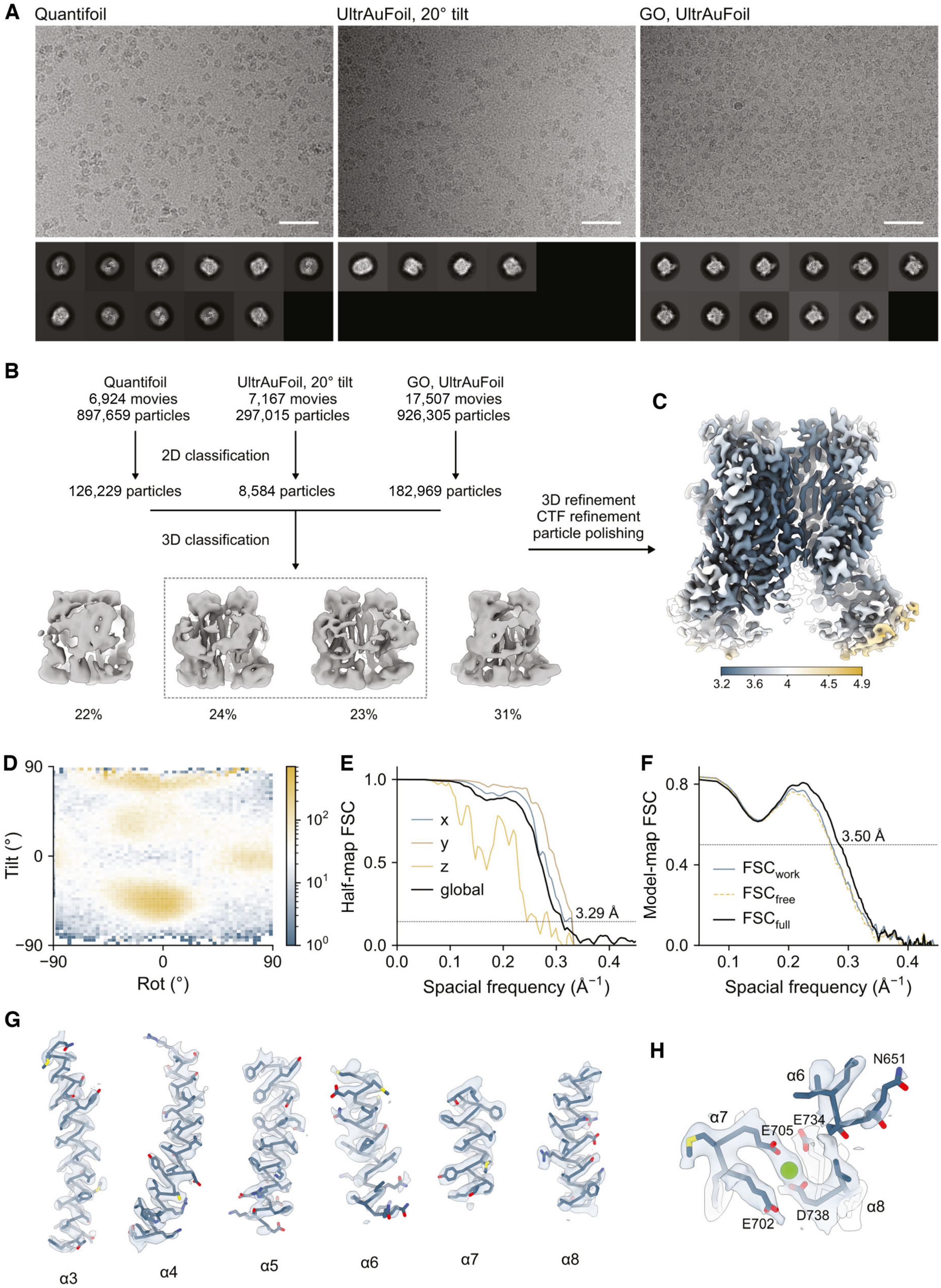

Figure EV2.

 © 2023 The Authors

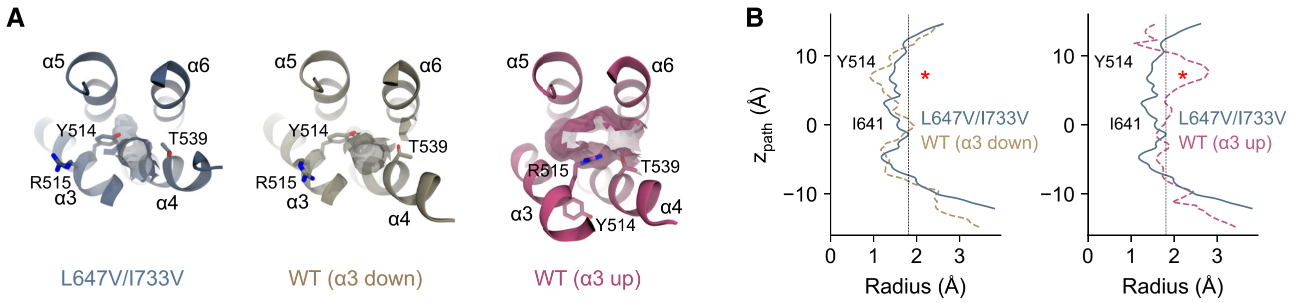

Figure EV3. Pore dimension.

A Molecular surface of the extracellular vestibule viewed from the top of the membrane. Selected residues lining the volume are shown. The bound 1PBC molecule in the WT (α3 up) model is omitted in the display for clarity. The models are as in Fig 4C.

B Pore radius along the z-axis relative to the position of Ile 641 (gate). The locations of constrictions are indicated. Asterisk indicates the location of the 1PBC-binding site. Dashed line denotes the ionic radius of a Cl^{-} ion.

© 2023 The Authors

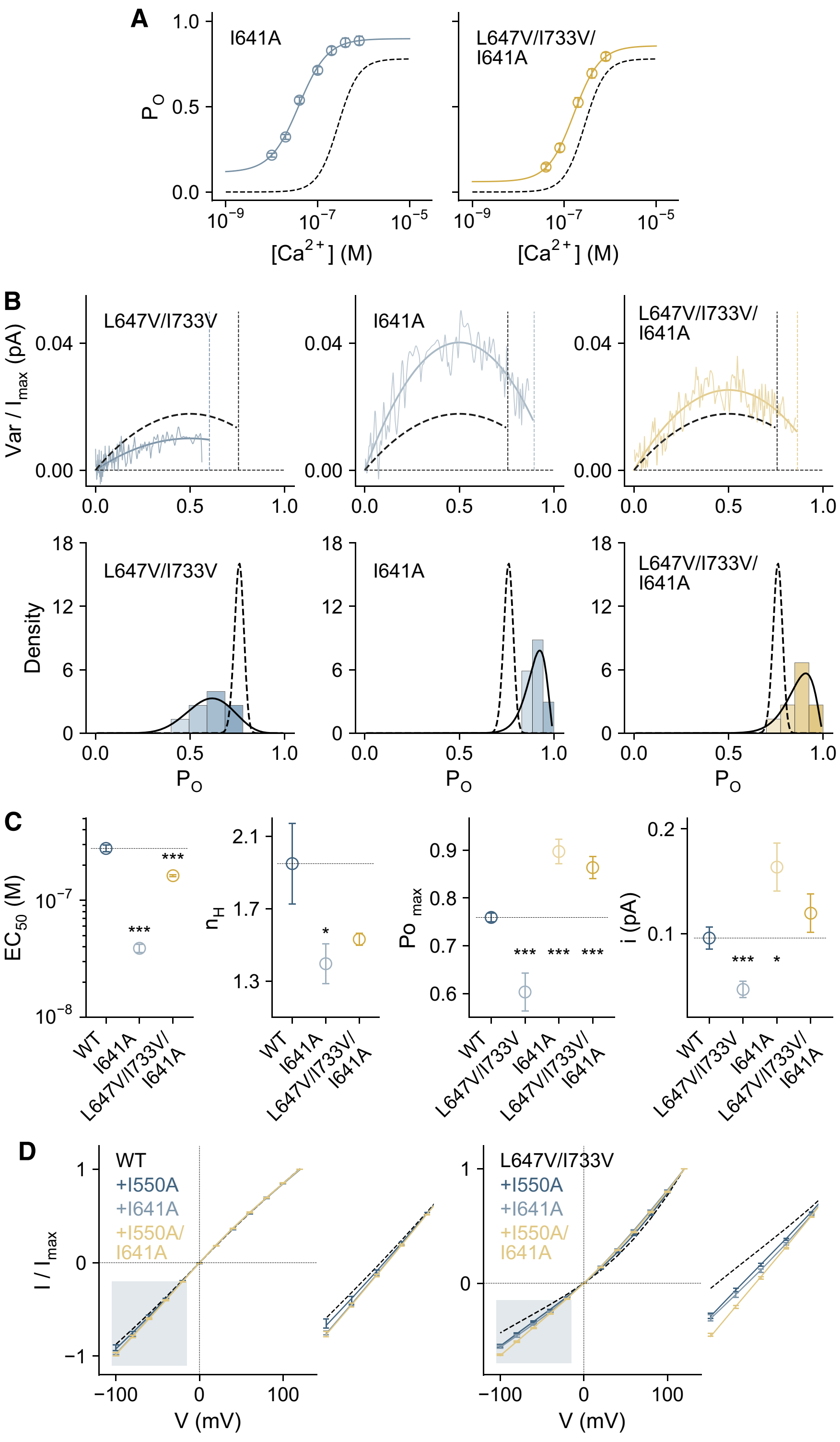

Figure EV4. Activation and conduction properties of mutants.

A Concentration-Po relations for the indicated mutants at +80 mV. Data are averages of 10 and 7 patches for I641A and L647V/I733V/I641A respectively, and errors are SEM. Solid line is a fit to the Hill equation. Dashed line is the relation of wild-type.

B Top, merged and averaged variance-current relations at a saturating Ca^{2+} concentration at −40 mV. Data are averages of 10, 6, and 10 patches for L647V/I733V, I641A, and L647V/I733V/I641A respectively. Solid line is a fit to Equation 5 in Supplementary Methods. Dashed line is the relation of wild-type. Dotted lines indicate the maximum Po. Bottom, histograms of the maximum Po obtained from individual measurements. Solid line is a fit to the beta distribution. Dashed line is the distribution of wild-type.

C EC_{50}, n_H, Po_{max}, and i of the indicated constructs. For EC_{50} and n_H, data are averages of 8, 10, and 7 patches for WT, I641A, and L647V/I733V/I641A respectively, and errors are SEM. For Po_{max} and i, data are averages of 8, 10, 6, and 10 patches for WT, L647V/I733V, I641A, and L647V/I733V/I641A respectively, and errors are SEM. *t*-test: $*P < 0.05$; $***P < 0.005$.

D Instantaneous I-V relations of the indicated mutants at a saturating Ca^{2+} concentration. Data are averages of 6, 6, 7, and 10 patches for WT, I550A, I641A, and I550A/I641A respectively (Left) and of 10, 8, 13, and 9 patches for L647V/I733V, L647V/I733V/I550A, L647V/I733V/I641A, and L647V/I733V/I550A/I641A respectively (Right), and errors are SEM. Solid lines are fits to a model of ion permeation (Equation 1 in Supplementary Methods) shown in Fig 3B. Dashed line is the relation of wild-type.

© 2023 The Authors

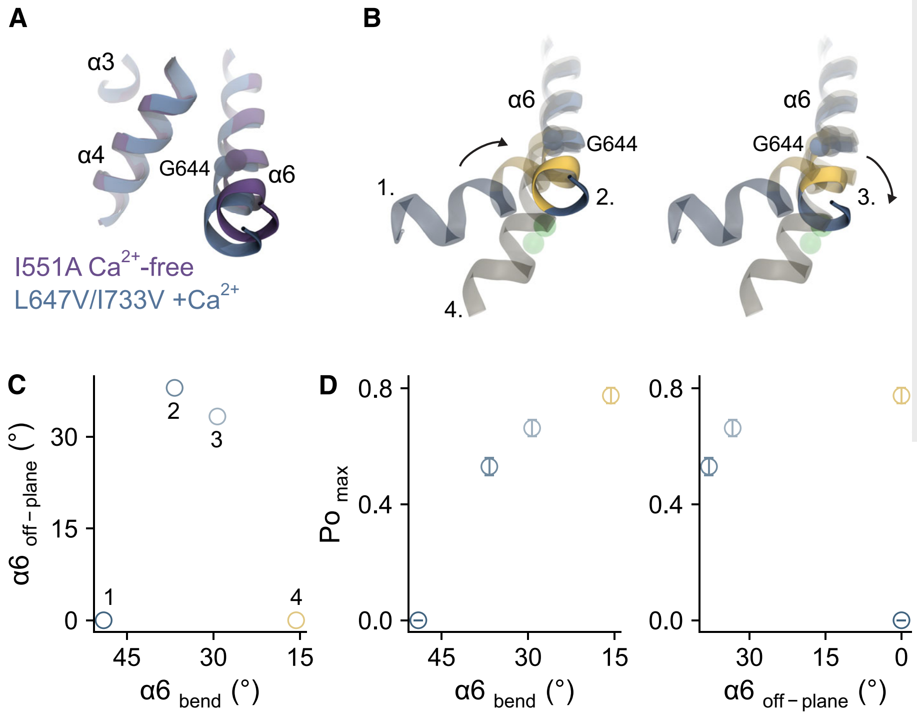

Figure EV5. α6 conformation and efficacy.

A Superposition of the I551A Ca^{2+}-free (PDBID 7B5D) and L647V/I733V + Ca^{2+} models. Selected helices are shown. The Cα of Gly 644 is shown as sphere.

B α6 conformations along its activation pathway. 1. WT Ca^{2+}-free (PDBID 5OYG), 2. I551A Ca^{2+}-free (PDBID 7B5D), 3. L647V/I733V + Ca^{2+}, and 4. WT + Ca^{2+} (PDBID 5OYB). The Cα of Gly 644 is shown as sphere. The bound Ca^{2+} in the wild-type model are shown as green spheres.

C Bending and off-plane movements below Gly 644 along the activation trajectory of α6. The plane defined by WT Ca^{2+}-free and WT + Ca^{2+} was used as a reference (0°).

D Relationship between efficacy and α6 conformations.

© 2023 The Authors
