# Supplementary material for: Mechanistic basis of ligand efficacy in the calcium‐activated chloride channel TMEM16A
Source: EMBO J. 2023 Nov 20;42(24):e115030. doi: 10.15252/embj.2023115030 (PMC10711664; doi:10.15252/embj.2023115030)
Supplement: Supplementary file 3 — PDF+ [file EMBJ-42-e115030-s001.pdf]

Article

SOURCE DATA TRANSPARENT PROCESS OPEN ACCESS

THE EMBO JOURNAL

Mechanistic basis of ligand efficacy in the calcium-activated chloride channel TMEM16A

Andy KM Lam* & Raimund Dutzler**

Abstract

Agonist binding in ligand-gated ion channels is coupled to structural rearrangements around the binding site, followed by the opening of the channel pore. In this process, agonist efficacy describes the equilibrium between open and closed conformations in a fully ligand-bound state. Calcium-activated chloride channels in the TMEM16 family are important sensors of intracellular calcium signals and are targets for pharmacological modulators, yet a mechanistic understanding of agonist efficacy has remained elusive. Using a combination of cryo-electron microscopy, electrophysiology, and autocorrelation analysis, we now show that agonist efficacy in the ligand-gated channel TMEM16A is dictated by the conformation of the pore-lining helix α6 around the Ca^{2+}-binding site. The closure of the binding site, which involves the formation of a π-helix below a hinge region in α6, appears to be coupled to the opening of the inner pore gate, thereby governing the channel's open probability and conductance. Our results provide a mechanism for agonist binding and efficacy and a structural basis for the design of potentiators and partial agonists in the TMEM16 family.

Keywords chloride channel; cryo-electron microscopy; efficacy; electrophysiology; ligand gating
Subject Category Structural Biology
DOI 10.15252/embj.2023115030 | Received 19 July 2023 | Revised 27 October 2023 | Accepted 2 November 2023 | Published online 20 November 2023
The EMBO Journal (2023) 42: e115030

Introduction

Agonist efficacy reflects the ability of the ligand to elicit a maximum response upon binding to its receptor. In ligand-gated ion channels (LGICs), this is manifested in the maximum open probability of the channel when the binding sites are saturated. Efficacy is governed by the equilibrium between open and closed states and their relative stability when the agonist is bound. The long-persisted view is that agonists work by shifting the equilibrium toward the open state for which they display a higher affinity, with more efficacious agonists necessarily being more selective for the open state (Colquhoun, 1998). Recent studies on nicotinic and glycine receptors have, however, shown that, instead of a concerted transition, channel activation involves an intermediate conformation that represents an activated, pre-open state where rearrangements at the ligand-binding site have likely occurred (Lape *et al*, 2008; Mukhtasimova *et al*, 2009; Jadey & Auerbach, 2012). Once this state is reached, the channel pore is able to open with similar efficiency even when bound to agonists with different efficacy that might elicit distinct conformations at the occupied binding site (Lape *et al*, 2008; Yu *et al*, 2021).

A similar mechanism underlying channel activation also applies to the ion channel TMEM16A, which opens in response to an increase in the intracellular Ca^{2+} concentration (Caputo *et al*, 2008; Schroeder *et al*, 2008; Yang *et al*, 2008). TMEM16A mediates important physiological processes such as epithelial chloride transport and smooth muscle contraction and has been proposed to be a therapeutic target for diseases including asthma, hypertension, stroke, and cystic fibrosis (Huang *et al*, 2012; Danahay *et al*, 2020; Al-Hosni *et al*, 2022; Galietta, 2022). The protein is a homodimer, with each subunit containing an ion conduction pore and a principal Ca^{2+}-binding site that are closely apposed (Dang *et al*, 2017; Paulino *et al*, 2017a, 2017b). Both pores act independently and are activated by the binding of two Ca^{2+} ions to the principal site (Jeng *et al*, 2016; Lim *et al*, 2016). Additional regulation is conferred by a proposed allosteric Ca^{2+}-binding site located remote from the ion conduction path and the lipid PIP_2 (Ta *et al*, 2017; Arreola & Hartzell, 2019; Le *et al*, 2019; Tembo *et al*, 2019; Yu *et al*, 2019; Le & Yang, 2020; Jia & Chen, 2021). In the absence of Ca^{2+}, the vacant principal binding site acts as an electrostatic gate to impede anion conduction, but is neutralized by Ca^{2+} binding to enable the channel to conduct with higher capacity (Lam & Dutzler, 2018). An equivalent process was proposed to account for ion selectivity changes in the scramblase TMEM16F, which also shows features of an ion channel (Ye *et al*, 2019). Besides the reversal of pore electrostatics, Ca^{2+} binding triggers a conformational change of the pore-lining helix α6, which rearranges to coordinate the bound Ca^{2+}, leading to the closure of the binding site (Paulino *et al*, 2017a). This movement is then propagated to the channel pore to release a hydrophobic gate at the inner pore entrance and to enable structural rearrangements in the outer vestibule and narrow neck region of the hourglass-shaped pore, which together open up a pathway to allow anion conduction (Lam *et al*, 2021, 2022; Lam &

Department of Biochemistry, University of Zurich, Zurich, Switzerland
*Corresponding author. Tel: +41 44 63 55546; E-mail: a.lam@bioc.uzh.ch
**Corresponding author. Tel: +41 44 63 56550; E-mail: dutzler@bioc.uzh.ch

© 2023 The Authors. Published under the terms of the CC BY 4.0 license.

Dutzler, 2021). The central role of α6 in channel activation has also been observed by others (Peters *et al*, 2018).

Channel gating in TMEM16A is a multi-state process that involves one or more intermediates prior to opening (Lam & Dutzler, 2021), akin to pentameric LGICs such as nicotinic and glycine receptors, where a pre-open intermediate, referred to as flipped or primed, has been observed (Lape *et al*, 2008; Mukhtasimova *et al*, 2009; Gupta *et al*, 2017). In TMEM16A, the initial transition leads to the first intermediate that displays a higher affinity for Ca^{2+} where the channel is in an activated yet still nonconducting state, as presumably represented by a Ca^{2+}-bound conformation (PDBID 5OYB) where α6 has rearranged and the binding site is closed while the pore has not yet opened. A second transition results in a pre-open state that is more accessible when two Ca^{2+} are bound and is plausibly a mechanistic counterpart of a conformation where the outer pore has expanded. This state is characterized by a movement of α3 and α4 where these pore-lining helices adopt an upward and outward conformation and can be stabilized by channel blockers that access from the extracellular side (PDBID 7ZK3) (Lam *et al*, 2022). These structural changes are reminiscent of an outer-pore gate that was proposed to open upon activation, which provides access to extracellular pore blockers (Dinsdale *et al*, 2021). It is notable that the "up" and "down" conformations of the α3/4 pair are both sampled in the Ca^{2+}-bound closed state (Paulino *et al*, 2017a; Lam *et al*, 2022), consistent with their relevance in channel gating. In this pre-open state, the channel is presumably primed to open, a motion that is not structurally well understood but corresponds to the release of a hydrophobic gate located at the inner entrance of the pore near the principal Ca^{2+}-binding site (Lam *et al*, 2021). Once fully bound by Ca^{2+}, channel opening is efficacious, indicating that the open state is energetically the most favorable amongst all accessible states (Lam & Dutzler, 2021).

Here, we studied the mechanisms underlying the efficacy of channel opening in TMEM16A by combining kinetic analysis and structural investigations. We show that ligand efficacy is dictated by the conformation of the pore-lining helix α6 and that the closure of the binding site, which involves the formation of a π-helix below a hinge region in α6, is coupled to the opening of the pore by weakening the interactions within the inner pore gate. Our results reveal the conformational coupling between different functional modules along the channel's activation pathway and identify α6 as a potential site for pharmacological intervention. Collectively, they provide a structural and mechanistic basis for the design of potentiators and partial agonists in the TMEM16 family.

Results

Agonist-coupled rearrangements at the binding site, efficacy, and cooperativity

Activation of LGICs is initiated by conformational changes around the ligand-binding site in response to agonist binding (Plested, 2016). In TMEM16A, this is manifested in a structural rearrangement of the pore-lining helix α6, which harbors residues that coordinate the bound agonist Ca^{2+} (Paulino *et al*, 2017a). Accompanying this hinge-like movement around a conserved glycine residue, this region transitions from an α-helical configuration to a π-helix, bringing an essential glutamate, Glu 654, into direct contact with the bound Ca^{2+} (Fig 1A). In addition to this ionic interaction, our previous study has identified several residues around the Ca^{2+}-binding site as important determinants of channel activation, which is manifested by a profound decrease in the potency of Ca^{2+} upon mutation to alanine (Lam *et al*, 2021). Here, we focus on Leu 647 on α6 and Ile 733 on α8, both within the π-helical region, which are brought into van der Waals contact in the Ca^{2+}-bound state upon α6 activation (Figs 1 and EV1). Decreasing the sidechain volume of these residues by mutation to valine or alanine severely impairs channel activation, as indicated by a pronounced right-shift in the potency of Ca^{2+} (Fig 1B and C, Appendix Table S1). The same mutations also reduce cooperativity as illustrated in the lowered Hill coefficient (n_H), which in the double mutant L647A/I733A approaches one (i.e., 1.3, Fig 1B and C, Appendix Table S1). While the potency is influenced by both Ca^{2+} binding and channel gating, nonstationary noise analysis indicates that this impairment in activation is partly due to a reduction in the channel's maximum open probability (Po_{max}) measured at a saturating ligand concentration, suggesting that Ca^{2+} has become less efficacious in these mutants (Figs 1D and E, and EV1C and E, Appendix Table S2). The reduction in Po_{max}, is accompanied by a decrease in the unitary current, as reflected in the initial slope of the noise parabola when Leu 647 and Ile 733 are mutated (Figs 1D and E, and EV1C and E, Appendix Table S2), which indicates a simultaneous perturbation of the anion conduction pathway. By analyzing different substituted aliphatic sidechains, we further observed that the severity of the gating defects correlates with the hydrophobic volume mediating this interaction, as exemplified in the trends of the potency (EC_{50}), cooperativity (n_H), and Po_{max} changes (Figs 1F and EV1D and E). Despite the general reduction in their cooperativity of activation, most of these mutants likely retain the ability to bind two Ca^{2+}, as reflected in their Hill coefficients, which remain consistently above one (Fig 1F, middle, and Fig EV1D, right). Together, these results suggest a hydrophobic interaction within the π-helical region close to the Ca^{2+}-binding site, which is mediated by Leu 647 on α6 and Ile 733 on α8, to be crucial in stabilizing the agonist-coupled rearrangement of α6. The impairment of this interaction has evidently structural consequences that impact on the efficacy of pore opening, the agonist binding process, and channel conductance in TMEM16A.

Agonist-coupled rearrangements stabilize the open state

As shown previously, channel gating in TMEM16A is a multi-state process that starts with a conformational change at the Ca^{2+}-binding site, followed by a transition into a pre-open state that is characterized by a widening of the outer pore, and finally the opening of the channel upon the release of a hydrophobic gate at the inner pore entrance (Lam & Dutzler, 2021) (Fig 2A). To understand how gating is affected when the agonist-coupled rearrangements are disrupted, we analyzed the transitions amongst these states in the L647/I733 series of mutants via autocorrelation analysis that we described previously (Lam & Dutzler, 2021) (Fig 2, Appendix Fig S1). In combination with nonstationary noise analysis, this approach allows the determination of the rate and equilibrium constants from steady-state macroscopic currents for a given mechanism, and thus the characterization of transition probabilities between states and their lifetimes (Lam & Dutzler, 2021). For the described mechanism, the estimated parameters likely

 © 2023 The Authors

represent a unique solution as a single, well-defined minimum of the sum-of-squares error, calculated using the experimental data, exists for each of the directly fitted rate constants within the observed time scales (Appendix Fig S2). Consistent with these properties, the confidence intervals of these parameters are typically within 10% of the fitted values (Appendix Table S3).

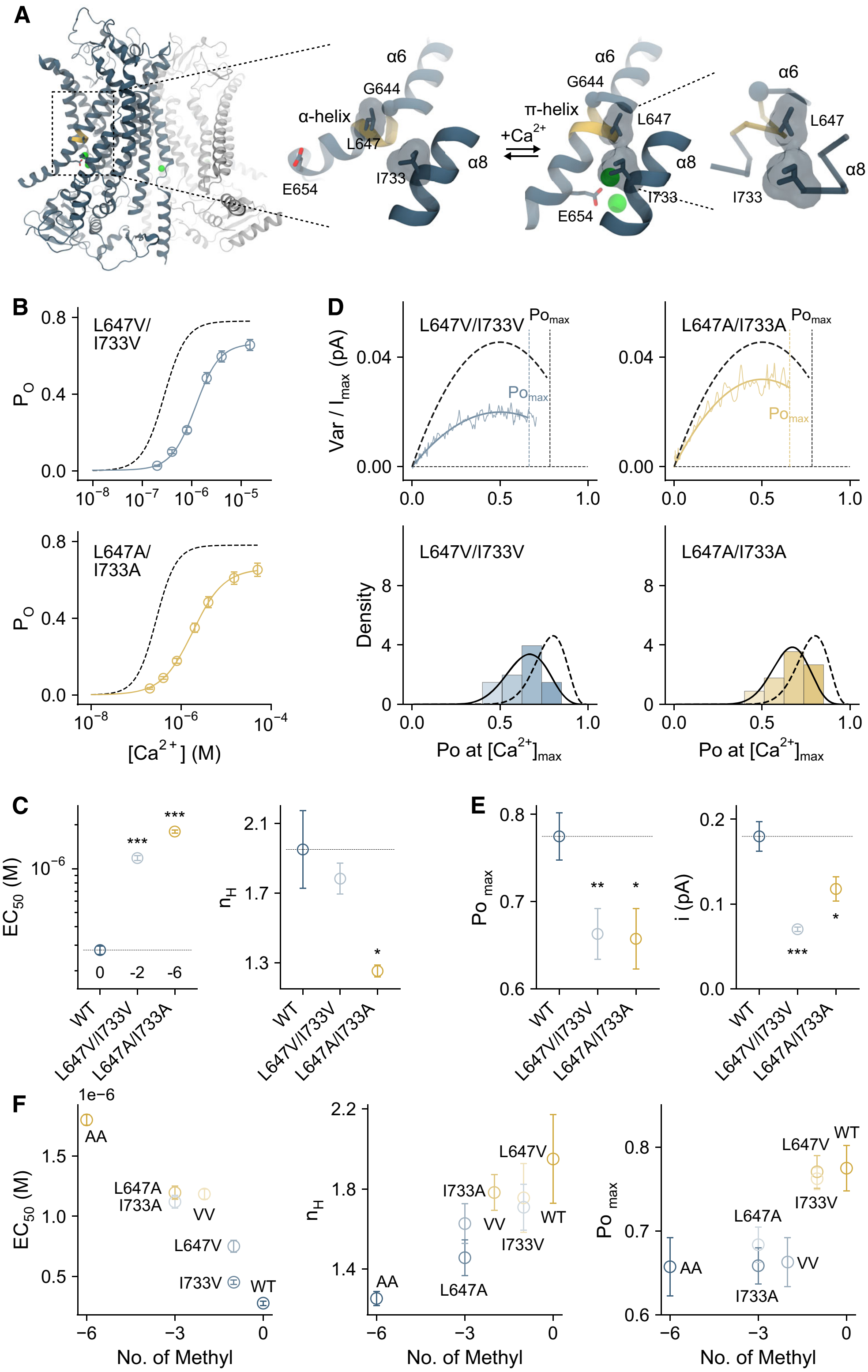

Figure 1.

Figure 1. Functional characterization of binding site rearrangement.

A Conformational rearrangement of α6 associated with Ca^{2+} binding. Left, structure of TMEM16A in a Ca^{2+}-bound state (Lam *et al*, 2022) viewed from within the membrane. The two subunits are displayed in unique colors. Middle, rearrangement of α6 upon Ca^{2+} binding. Selected helices are shown as ribbon, sidechains as sticks, the Cα of Gly 644 as sphere, and bound Ca^{2+} as green spheres. Right, close-up view of the π-helical region in Cα representation. The volume of the sidechains of Leu 647 and Ile 733 is shown in a surface representation. The region undergoing the α-to-π-helix transition is shown in yellow. PDBID 5OYG (Ca^{2+}-free) and 7ZK3 (Ca^{2+}- and 1PBC-bound) are displayed.

B Concentration-Po relations for the indicated mutants at +80 mV. Data are averages of 8 and 5 patches for L647V/I733V and L647A/I733A respectively, and errors are SEM. Solid line is a fit to the Hill equation. Dashed line is the relation of wild-type.

C EC_{50} and n_H of the indicated constructs. Shown are the averages of the data displayed in (B) and errors are SEM. The number of methyl groups truncated relative to wild-type is indicated in the left panel. $n = 8$, 8, and 5 for WT, L647V/I733V, and L647A/I733A respectively. *t*-test: $***P < 0.005$.

D Top, merged and averaged variance-current relations at a saturating Ca^{2+} concentration at +80 mV. Data are averages of 20 and 10 patches for L647V/I733V and L647A/I733A recorded at 15 μM and 50 μM Ca^{2+} respectively. Solid line is a fit to Equation 5 in Supplementary Methods. Dashed line is the relation of wild-type. Dotted lines indicate the respective maximum Po's. Bottom, histograms of the maximum Po obtained from individual measurements. Solid line is a fit to the beta distribution. Dashed line is the distribution of wild-type.

E Po_{max} and i of the indicated constructs. Shown are the averages of the data displayed in (D), and errors are SEM. $n = 11$, 20, and 10 for WT, L647V/I733V, and L647A/I733A respectively. *t*-test: $*P < 0.05$; $**P < 0.01$; $***P < 0.005$.

F EC_{50}, n_H, and maximum Po as a function of hydrophobic volume. For EC_{50} and n_H, data are best-fit values, and errors are standard errors. For maximum Po, data are averages, and errors are SEM. Each data point corresponds to the properties of a mutant in the L647/I733 series shown in this figure, Fig EV1, and Appendix Tables S1 and S2. WT, $n = 8$; L647V, $n = 7$; I733V, $n = 8$; L647V/I733V, $n = 8$; L647A, $n = 8$; I733A, $n = 8$; L647A/I733A, $n = 5$. VV, L647V/I733V; AA, L647A/I733A.

Source data are available online for this figure.

We found that disrupting the interaction between Leu 647 and Ile 733 energetically destabilizes the open state as the forward equilibrium constant (L_{open}) is considerably lowered in the L647/I733 series of mutants (Fig 2B, Appendix Table S3). An opposite effect was observed for the pre-opening step (described by $L_{pre\text{-}open}$), where perturbing the L647/I733 interaction promotes the transition into this intermediate state (Fig 2B, Appendix Table S3). Unexpectedly, the equilibrium of the initial agonist-coupled transition (described by $L_{\alpha6}$) is only minimally affected (Fig 2B, Appendix Table S3), which might point toward a negligible role of both residues in this step, or alternatively a structurally distinct but energetically similar rearrangement at the Ca^{2+}-binding site. The latter could be due to a compensatory effect where the absence of the energetically costly π-helix conformation (see later) might be offset by an inadequate coordination of the bound Ca^{2+} in the mutants. The decreased stability of channel opening is primarily attributable to the shortening of the open-state lifetime (τ_{open}) (Fig 2C), resulting in a higher tendency to transition into closed states. By analyzing single and double mutants in a double-mutant cycle, our data further confirm a functional interaction between Leu 647 and Ile 733 in stabilizing the agonist-coupled rearrangement of α6 as the individual mutations L647A and I733A perturb the opening and pre-opening transitions almost as much as their combination (Fig 2B). The polarity of the resulting coupling energies reflects the contribution of the L647/I733 interaction in stabilizing the open state while limiting the pre-opening transition (Fig 2D). The calculated equilibrium occupancies, using the estimated parameters, suggest that the mutant L647A/I733A is more likely to reside in the pre-open state in expense of the open state (Fig 2E).

To gain insights into the timing of the affected rearrangements during the opening step, we analyzed the relationship between the rate and equilibrium constants of this transition in the L647/I733 series of mutants (Fig 2F). The relative timing of the perturbed motion can be characterized by its phi value (ranging between 0 and 1), which is reflected in the slope of the rate-equilibrium relation (Leffler, 1953; Grosman *et al*, 2000; Sorum *et al*, 2015). A phi value of close to zero corresponds to a scenario where the mutations affect primarily the final state, indicative of a rearrangement that occurs relatively late on the reaction coordinate, whereas a value close to one suggests a rearrangement that occurs early on the reaction coordinate. In the L647/I733 series of mutants, the closing rate (the backward rate constant k_b) is affected almost as much as the forward equilibrium constant (Fig 2F), indicating a rather selective perturbation of the open state. The experimental phi value of 0.08 for L_{open} suggests that the L647/I733 interaction and α6 rearrangement facilitate a structural change that occurs relatively late in the opening transition (Fig 2F). Notably, a direct disruption of the gate results in a phi value of zero for the opening transition (L_{open}) as observed in our previous study (Lam & Dutzler, 2021), showing a close correspondence with the motion facilitated by the L647/I733 interaction during this same transition. This further emphasizes the role of these two residues in allosterically stabilizing the open state.

Agonist-coupled rearrangements stabilize an open-pore geometry

Nonstationary noise analysis of the investigated mutants indicates that, in addition to a decrease in the Po_{max}, the conductance of the channel is considerably lower when the agonist-coupled rearrangements are disrupted (Figs 1D and E, and EV1B and E, Appendix Table S2). To understand how the properties of the pore are affected, we analyzed the current–voltage (I-V) relations and extracted the energetic effects of the L647/I733 series of mutations on ion conduction (Fig 3A, Appendix Table S4). Changes in the energetics of anion diffusion and the nature of the resulting current rectification depend on the position of the perturbations with respect to the ion conduction path, allowing the localization of the underlying structural changes (Paulino *et al*, 2017b). Energies were obtained from a fit to a three-barrier model that was introduced previously to approximate the rate-limiting steps for the diffusion of an ion across the narrow region of the hourglass-shaped pore. This analysis yields kinetic parameters (σ_β and σ_h) that reflect the energy difference of the inner and central barrier, related to the entry of the anion from the inside and its passage through the neck region, relative to the outer barrier that describes the release of the anion to the extracellular part of the pore (Fig 3B and C). When the I-V plots are placed on a unitary scale,

© 2023 The Authors

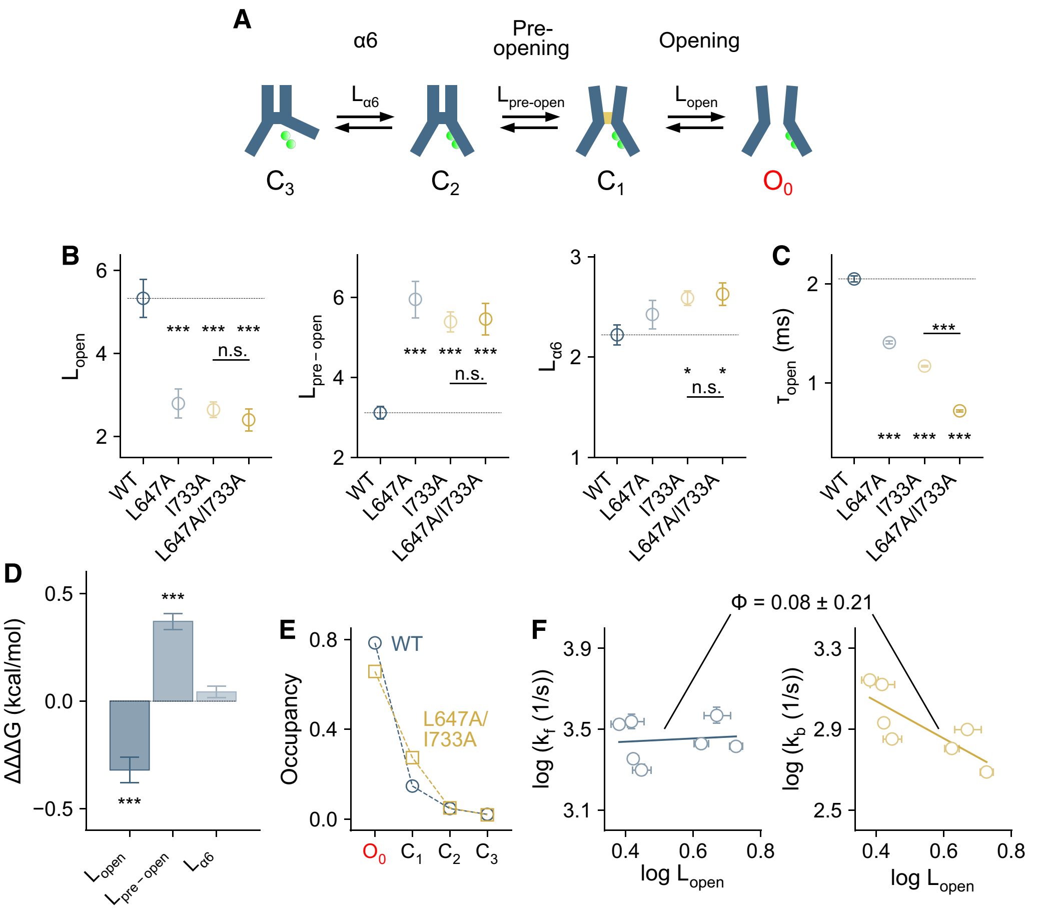

Figure 2. Binding site closure, efficacy, and gating mechanism.

A Mechanism depicting gating transitions at saturating Ca^{2+} concentrations. The minimum number of states that can account for the gating properties of the wild-type channel is shown (Lam & Dutzler, 2021). This scheme comprises three closed states, interpreted as α6-resting (C_3), α6-activated (C_2), and nonconductive outer-pore-open (C_1), and one open state in which the inner pore gate is opened (O_0). A sequential model is likely approximative, but is reasonable given that channel opening is minimal in the α6-resting (C_3) state. Additional states/transitions might exist, although they likely occur with a much lower probability and are therefore difficult to detect and characterize functionally.

B Forward equilibrium constants for the indicated transitions estimated from autocorrelation analysis (Equations 7–9 in Supplementary Methods, see Appendix Fig S1). Data are best-fit values of the averaged spectra from 7, 5, 6, and 7 patches for WT, L647A, I733A, and L647A/I733A respectively, and errors are 95% confidence intervals. Dashed line indicates the value of wild-type. *t*-test: n.s., nonsignificant; $*P < 0.05$; $***P < 0.005$.

C Mean open time calculated from the estimated rate constant k_{01}. Data were calculated from the best-fit values, and errors are 95% confidence intervals. The number of replicates is as in (B). *t*-test: $***P < 0.005$.

D Coupling energies (ΔΔΔG) for the indicated transitions. Bars indicate quantities calculated using Equation 10 in Supplementary Methods, and errors are standard errors. The number of replicates is as in (B). *t*-test: $***P < 0.005$.

E Equilibrium occupancy of states calculated, respectively, with the best-fit values of wild-type and L647A/I733A using Equation 7 in Supplementary Methods.

F Rate-equilibrium relations for the opening transition. Data are best-fit values, and errors are 95% confidence intervals. Error bars are not visible when they are smaller than the symbols. k_f and k_b are the forward and backward rate constants respectively. Solid lines are a simultaneous fit to a pair of rate-equilibrium relations (Equation 11 in Supplementary Methods), yielding a single phi value (best-fit ± standard error) for the forward and backward transitions. Each data point corresponds to the properties of a mutant in the L647/I733 series shown in Appendix Fig S1 and Appendix Table S3.

Source data are available online for this figure.

this analysis further allows the estimation of changes in the outer barrier (β/β_{WT}), and thus a more complete description of the ion conduction path (Fig 3C).

We found that disrupting the L647/I733 interaction lowers the overall conductance of the channel (Figs 3A and EV1E, Appendix Fig S3A, Appendix Table S2), owing to a considerable elevation of energy barriers at the intracellular entrance of the neck (σ_β) and inside the narrow pore (σ_h), whereas the outer barrier (β/β_{WT}) is largely unaffected for sidechain replacements with valine and is somewhat decreased in the case of alanine mutations (Fig 3C, Appendix Fig S3B). This is manifested in the pronounced outward rectification of the mutants L647V and L647V/I733V, indicating a more constricted geometry both at the inner pore and the narrow neck region (Fig 3A and C). We analyzed these energetic effects in a double-mutant cycle and observed a functional interaction between Leu 647 and Ile 733 in stabilizing a more dilated pore geometry, as

© 2023 The Authors

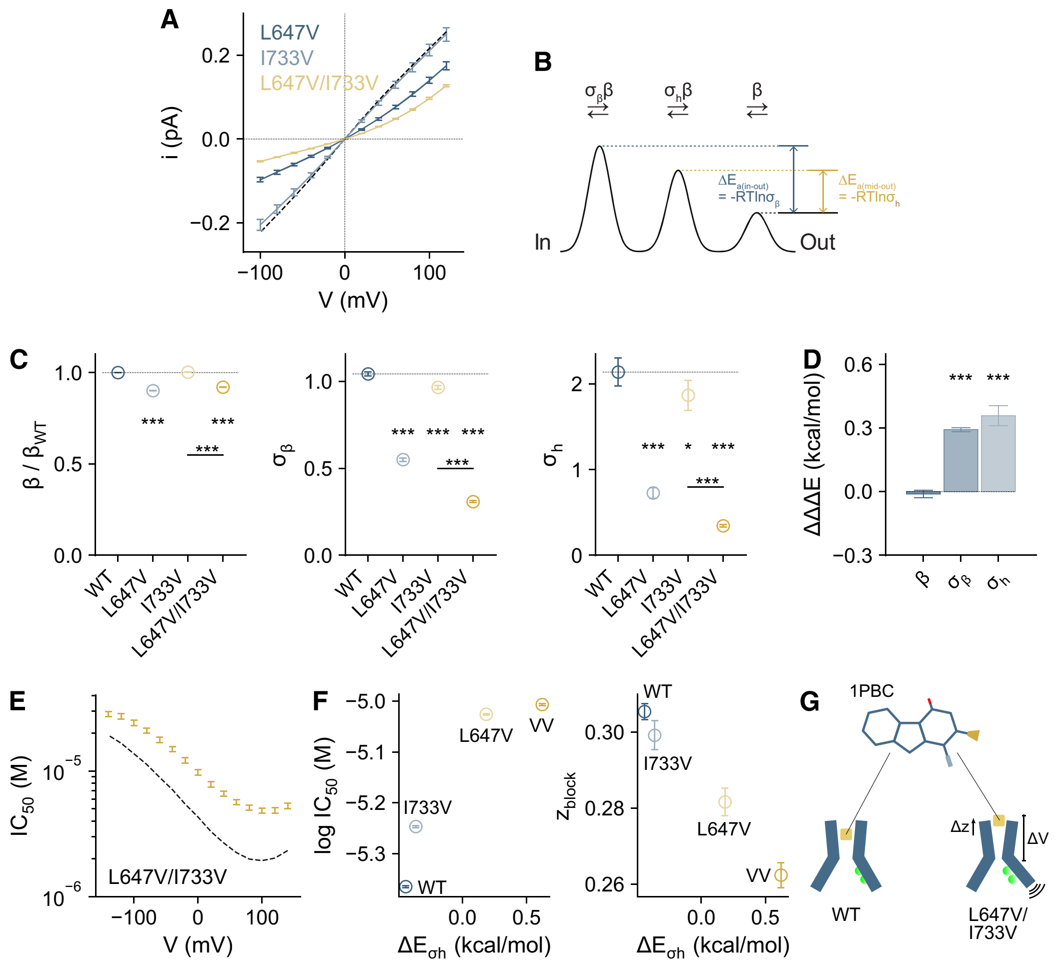

Figure 3. Binding site closure, ion conduction, and pore conformation.

A Instantaneous current–voltage (I-V) relations of the indicated mutants at a saturating Ca^{2+} concentration on a unitary scale. Data were scaled according to the estimated unitary current from nonstationary noise analysis at +80 mV (Figs 1 and EV1, Appendix Table S2). Data are averages of 6, 8, 8, and 6 patches for WT, L647V, I733V, and L647V/I733V respectively, and errors are SEM. Solid lines are fits to a model of ion permeation shown in (B) (Equation 1 in Supplementary Methods). Dashed line is the relation of wild-type.

B Energy profile of a minimal ion permeation model to account for the I-V relations (Paulino *et al*, 2017b).

C Conduction parameters for the indicated barriers. Data are best-fit values, and errors are 95% confidence intervals. Dashed line indicates the value of wild-type. The number of replicates is as in (A). *t*-test: $^{*}P < 0.05$; $^{***}P < 0.005$.

D Coupling energies (ΔΔΔE) for the indicated barriers. Bars indicate quantities calculated using Equations 2, 3, and 10 in Supplementary Methods, and errors are standard errors. The number of replicates is as in (A). *t*-test: $^{***}P < 0.005$.

E Inhibition by the pore blocker 1PBC as a function of voltage in L647V/I733V. Data are best-fit values from a fit to the Hill equation using averaged concentration-response curves from 7 patches at the indicated voltages, and errors are 95% confidence intervals. Dashed line is the relation of wild-type.

F Correlation between the blocking properties of 1PBC and the magnitude of the energy barrier in the narrow neck region of the pore ($\Delta E_{\sigma h}$, calculated from the data displayed in (C)). The IC_{50} values at 0 mV are plotted. z_{block} is equivalent to the fraction of the transmembrane electric field operating on the blocker at its binding site. Data are the best-fit values from a fit to Equation 4 in Supplementary Methods in the exponential region (±40 mV) of the relations shown in (E) and Appendix Fig S3E and errors are standard errors determined from 6, 6, 6, and 7 patches for WT, L647V, I733V, and L647V/I733V respectively. The number of replicates for $\Delta E_{\sigma h}$ is as in (A).

G Schematic summarizing the data. Disrupting binding site closure in the mutant L647V/I733V leads to a partial collapse of the pore and structural perturbations at the 1PBC-binding pocket. The narrowing of the pore is accompanied by a shift of the blocker binding site towards the extracellular side.

Source data are available online for this figure.

reflected in the nonadditive effects of these mutations (Fig 3D, Appendix Fig S3C). These results suggest an allosteric process that relays the conformation of the binding site to the widening of the ion conduction path in the open state, as both residues are located away from the pore.

A change in the pore geometry in the narrow neck region in this set of mutants is further supported by the binding properties of the blocker 1PBC (Peters *et al*, 2015) in the open pore. We previously showed that 1PBC binds at the extracellular opening of the narrow neck region, and that the binding of 1PBC is in part governed by

 © 2023 The Authors

steric complementarity (Lam *et al*, 2022). Consistent with a coordinated conformational change of the pore helices, impaired α6 rearrangement is associated with putative structural changes at the outer entrance of the narrow neck, as suggested by the lower affinity of 1PBC in L647V/I733V and related mutants, with varying degrees of severity (Fig 3E and F, Appendix Fig S3D–F). This is accompanied by a decrease in the voltage sensitivity and the valence of block, z_{block}, which reflects the location of the binding site perpendicular to the membrane plane, indicating that 1PBC penetrates less into the channel pore from the extracellular side when α6 rearrangement is disrupted (Fig 3E and F, Appendix Fig S3D–F). In this group of mutants, the lowering of z_{block} correlates strongly with the elevation of the energy barrier inside the narrow neck, confirming a partial collapse of the pore and a steric origin of the elevated barriers for ion conduction (Fig 3F). Together, these results show that the conformational state of α6 does not only dictate the channel's opening efficacy but also the degree of pore opening (Fig 3G).

α6 conformation dictates efficacy

To understand the rearrangements underlying the efficacy of pore opening, we determined a cryo-EM structure of L647V/I733V, whose gating properties are similarly affected compared to the less conservative mutant L647A/I733A but which is characterized by a more severely perturbed ion conduction pathway (Figs 1B–E and 3A, Appendix Fig S3A), in the presence of a saturating Ca^{2+} concentration (Figs 4A and B, and Fig EV2). The reconstruction was obtained by combining datasets collected from samples prepared on cryo-EM grids with distinct chemical properties, each showing a distinct preferred orientation of particles and at different tilts. Together, the pooled datasets yielded complementary particle views for the final map which, despite the remaining anisotropy, was much improved and provided a largely undistorted view of the protein (Table 1, Fig EV2).

Single-particle analysis of L647V/I733V reveals a major conformation that is distinct from the wild-type channel (Fig EV2). With an overall resolution of 3.29 Å, the final map shows well-defined density for the entire protein, including the bound agonist Ca^{2+} in its principal binding site (Fig EV2G and H). In this structure, the disruption of the L647/I733 interaction results in an incomplete rearrangement of α6, with the helix adopting an intermediate, partially activated conformation (Fig 4C and D). The described change in conformation is accompanied by a loss of the density corresponding to the Ca^{2+}-binding part of α6, indicating that the helix has become more mobile and that the interaction with bound Ca^{2+} is substantially weakened (Fig EV2G and H). The incomplete rearrangement of α6 is accompanied by a partial collapse of the extracellular part of the pore-lining helix α4 toward α6 (Fig 4C, left and Fig EV3), which is evident when compared with the 1PBC-bound, outer-pore-open conformation (Fig 4C, right and Fig EV3). This might in part explain the elevated steric barriers for ion conduction and iblocker access in this mutant as observed in electrophysiological experiments (Fig 3A, C and F).

As a consequence of the weakened interaction between α6 and α8, the protein is in a state containing a single well-resolved Ca^{2+} ion in the principal site, even at a saturating Ca^{2+}concentration, suggesting that in the mutant, this site might accommodate only one ligand (Figs 4E and EV2H). The concomitance of these structural changes has also been observed for the wild-type channel solubilized in LMNG, although its relevance to channel function has remained unclear (Dang *et al*, 2017). The single-bound Ca^{2+} is in a position similar to the lower ion in the doubly bound state and is coordinated by two acidic residues, Glu 705 on α7 and Asp 738 on α8, with no coordinating residues from α6 since its rearrangement is impaired (Fig 4D and E). Despite in a Ca^{2+}-bound state, the region below the conserved glycine hinge remains an α-helix (Fig 4F), suggesting that the L647/I733 interaction and the second bound Ca^{2+} in the upper position are needed to stabilize the strained π-helix conformation corresponding to α6 in a fully activated state. Reciprocally, the formation of a π-helix in this region would bring these residues into the correct register that maximizes the respective protein-ligand and α6-α8 interactions. Together, these structural data suggest that the L647/I733 interaction promotes pore opening and contributes to the positive cooperativity of ligand binding by priming and stabilizing the complete closure of the binding site.

Conformational coupling between the agonist-binding site and the gate underlies ligand efficacy

In TMEM16A, the channel gate is located at the intracellular pore entrance and is formed by three pore-lining hydrophobic residues (Ile 550, 551, and 641) residing on α-helices 4 and 6, with Ile 641 being the most prominent contributor (Lam *et al*, 2021; Lam & Dutzler, 2021). Since Ile 641 is one helix turn above the glycine hinge on α6 and the L647/I733 interaction takes place one helix turn below, we hypothesized that defective rearrangements below the hinge may exert a direct influence on the functioning of the gate (Fig 5A). We investigated this scenario by constructing a double-mutant cycle consisting of L647V/I733V and I641A and analyzed whether the effect of L647V/I733V on decreasing efficacy depends on the stability of the gate (Figs 5 and EV4). Consistent with this hypothesis, L647V/I733V is unable to reduce the efficacy of pore opening and shorten the open state lifetime in the mutant I641A, where the disruption of the gate has profoundly stabilized the open state. This is reflected in the similar values of the respective L_{open} and τ_{open}, revealing a functional coupling between the agonist-coupled movements and the opening of the gate (Fig 5B and C, Appendix Table S3). Reciprocally, I641A, which opens the gate, fails to promote agonist-coupled rearrangements when the L647/I733 interaction is impaired in the mutant L647V/I733V, yielding a similar value of $L_{\alpha 6}$ as WT (Fig 5B, Appendix Table S3). This is in sharp contrast to the strongly increased transition observed in the single alanine mutant of this residue.

The interdependence between the gate and α6 rearrangement extends to the extent of pore opening (i.e., the size of the open pore), in which I641A partially reversed the inner pore collapse caused by L647V/I733V (Fig 5D–F). This is manifested in the somewhat reduced current rectification of the triple mutant (Fig 5D) and the magnitudes of the inner and central barriers for ion conduction (Fig 5E), where the effect of I641A is more pronounced on the L647V/I733V background compared to WT. This indicates that the more constricted pore in L647V/I733V is due to a partial obstruction by the gate residue Ile 641, which in turn suggests an incomplete release of the gate in the open state of this mutant. The disparity in the effect of I641A on the L647V/I733V and WT backgrounds in both

© 2023 The Authors

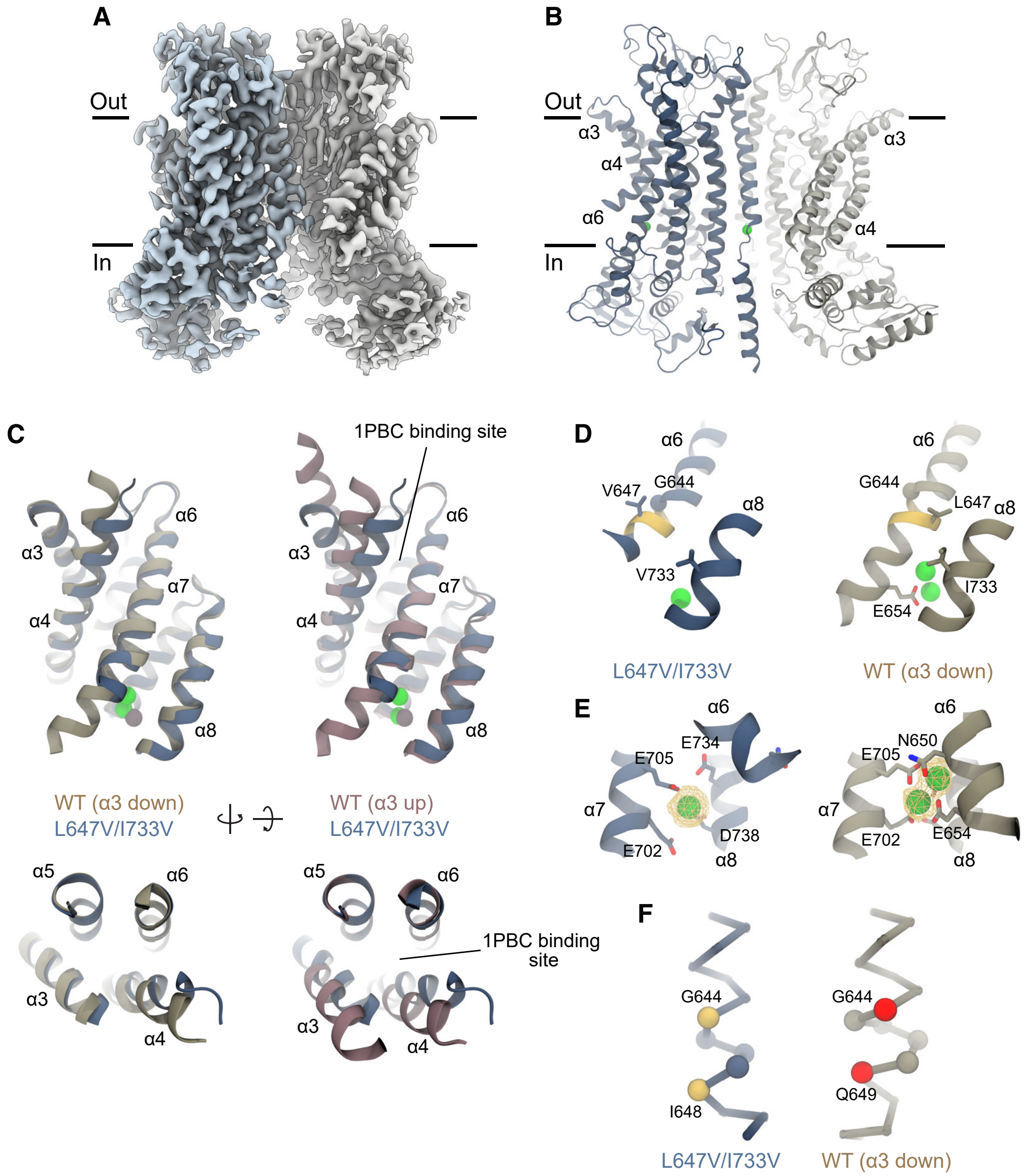

Figure 4. Structural basis for partial efficacy.

A, B Cryo-EM map (A) and ribbon representation (B) of L647V/I733V at a saturating Ca^{2+} concentration in the detergent GDN viewed from within the membrane. Black lines indicate membrane boundaries.

C Superposition of the pore region of the L647V/I733V with the wild-type Ca^{2+}-bound (PDBID 5OYB, "α3 down" conformation rebuilt in Lam *et al*, 2022) and the wild-type Ca^{2+}/1PBC-bound (PDBID 7ZK3, "α3 up" conformation where the outer pore is open; Lam *et al*, 2022) structures viewed from within the membrane (Top) and from the extracellular side (Bottom). Bound Ca^{2+} are shown as purple and green spheres in the L647V/I733V and wild-type models respectively.

D, E α6 conformations in the Ca^{2+}-bound state (D) and Ca^{2+}-binding site (E). Selected helices are shown as ribbon, sidechains as sticks, the Cα of Gly 644 as sphere, and bound Ca^{2+} as green spheres. The region undergoing an α-to-π-helix transition in wild-type is highlighted in yellow. The densities for the bound Ca^{2+} ions are shown in (E).

F Section of α6 around Gly 644 in Cα representation. Yellow and red spheres depict pairs of hydrogen-bonded positions in α-helix and π-helix conformations respectively. The Cα in between are shown as spheres.

gating (Fig 5B and C) and ion conduction (Fig 5D and E) results in nonzero coupling energies (Fig 5F), indicating that the opening of the gate and α6 rearrangement do not operate independently but that their conformational transitions are coupled.

The decrease in the opening efficacy and the incomplete release of the gate prompted us to investigate whether these are associated with a tighter interaction within the gate when α6 rearrangement is impaired. To test this, we constructed a

 © 2023 The Authors

Table 1. Cryo-EM data collection, processing, refinement, and validation statistics.

	Quantifoil	UltrAuFoil, 20°	GO, UltrAuFoil
Data collection and processing			
Microscope	FEI Titan Krios G3i	FEI Titan Krios G3i	FEI Titan Krios G3i
Camera	Gatan K3 GIF	Gatan K3 GIF	Gatan K3 GIF
Imaging mode	Super-resolution counted	Super-resolution counted	Super-resolution counted
Magnification	130,000	130,000	130,000
Voltage (kV)	300	300	300
Energy filter slit width (eV)	20	20	20
Electron dose (e^-/Å^2)	66.1	66.1	62.5
Defocus range (μm)	−2.4 to −1.0	−2.4 to −1.0	−2.4 to −1.0
Pixel size (Å)[a]	0.659 (0.3295)	0.659 (0.3295)	0.659 (0.3295)
Initial particle images (no.)	897,659	297,015	926,305
Final particle images (no.)	103,964	6,242	34,728
Symmetry imposed	C2		
Map resolution (Å)<br>FSC threshold 0.143	3.29		
Map resolution range (Å)	3.2–4.9		
Refinement			
Initial model	PDBID 7B5D		
Model resolution (Å)<br>FSC threshold 0.5	3.50		
Map sharpening B factor (Å^2)	−91.3		
Model composition			
Non-hydrogen atoms	11,468		
Protein residues	1,402		
Ligands	Ca^{2+}: 4		
B factors (Å^2)			
Protein	54.4		
Ligand	33.7		
r.m.s. deviations			
Bond lengths (Å)	0.004		
Bond angles (°)	0.813		
Validation			
MolProbity score	1.46		
Clash score	3.67		
Poor rotamers (%)	0.00		
Ramachandran plot			
Favored (%)	95.6		
Allowed (%)	4.40		
Disallowed (%)	0.00		

[a]Values in parentheses indicate the pixel size in super-resolution.

double-mutant cycle consisting of the two gate residues I550A on α4 and I641A on α6 on the L647V/I733V and WT backgrounds and compared the respective coupling energies between Ile 550 and Ile 641 derived from the magnitudes of the inner barrier in the open pore (Figs 5G and EV4D). Compared to the WT background, the coupling energy between the gate residues Ile 550 and Ile 641 is increased by about 4-fold in the L647V/I733V background (Fig 5H), indicating that these gate residues indeed interact more tightly when α6 rearrangement is disrupted. Together, these results suggest a mechanism in which the

© 2023 The Authors

agonist-coupled rearrangements weaken interactions within the inner pore gate and vice versa.

Discussion

In this study, we combined kinetic analysis and structural investigations to address the molecular basis governing agonist efficacy in the ligand-gated channel TMEM16A. Using mutagenesis and autocorrelation analysis, we have shown that disrupting agonist-coupled rearrangements at the binding site allosterically impairs channel opening by decreasing the stability of the open state (Figs 1 and 2). The structure of a mutant with defective binding site rearrangement reveals a distinct conformational change that underlies the decrease in the channel's opening efficacy and suggests a role for the α-to-π-helix transition of α6 in structurally coupling binding site closure and the release of the inner pore gate (Fig 4). This mechanism is supported by the analysis of several double-mutant cycles, showing that agonist-dependent α6 closure destabilizes the hydrophobic gate at the inner pore, thereby enhancing the efficacy and the degree of

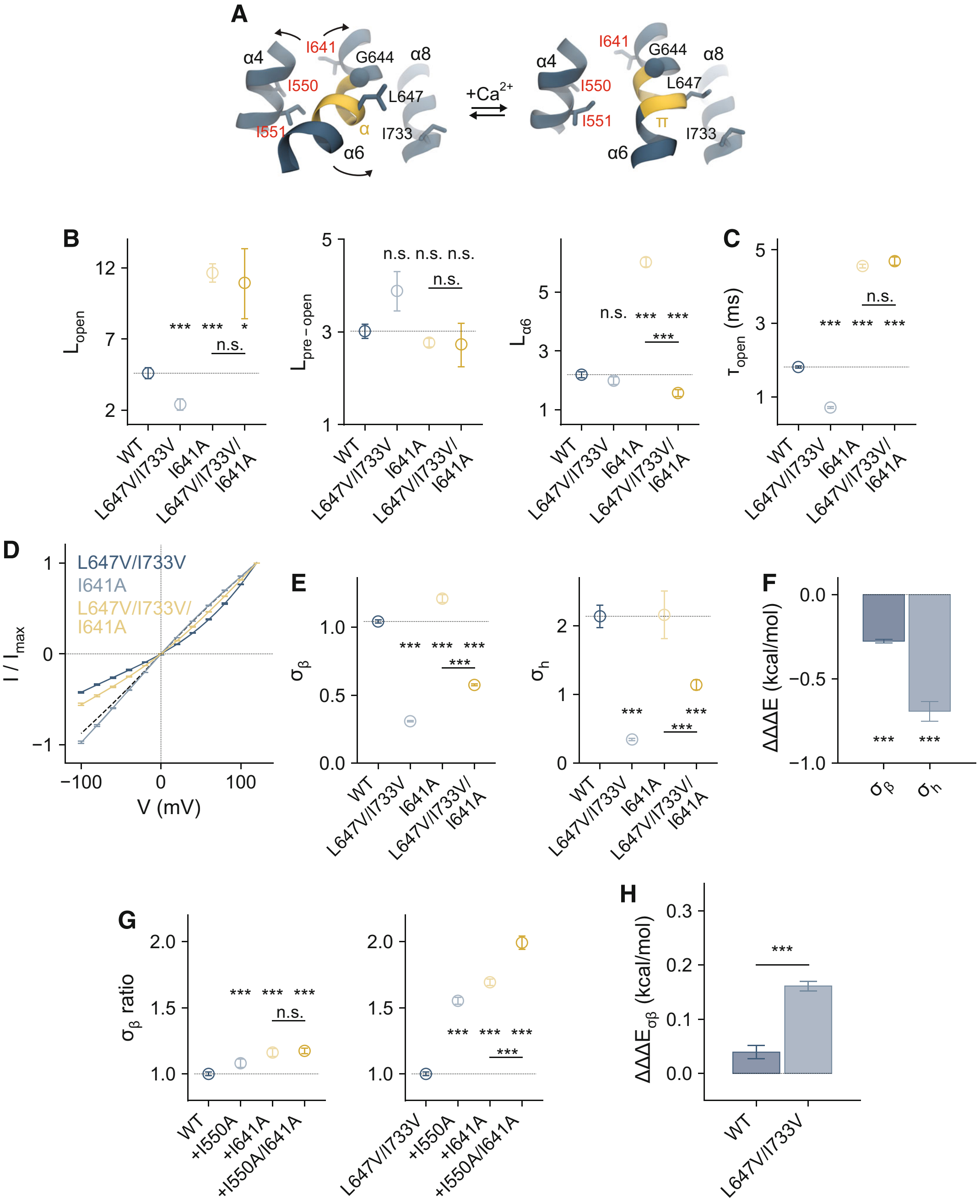

Figure 5.

 © 2023 The Authors

Figure 5. Coupling binding site closure and α6 rearrangement to channel opening.

A Rearrangements of the gate region and around the binding site upon Ca^{2+} binding. Selected helices are shown as ribbon, sidechains as sticks, and the Cα of Gly 644 as sphere. The region undergoing an α-to-π-helix transition is highlighted in yellow. Residues forming the gate are labeled in red. PDBID 5OYG (Ca^{2+}-free) and 5OYB (Ca^{2+}-bound) (Paulino *et al*, 2017a) are displayed.

B Forward equilibrium constants for the indicated transitions obtained from autocorrelation analysis (Equations 7–9 in Supplementary Methods, see Appendix Fig S1). Data are best-fit values of the averaged spectra from 7, 5, 7, and 6 patches for WT, L647V/I733V, I641A, and L647V/I733V/I641A respectively, and errors are 95% confidence intervals. Dashed line indicates the value of wild-type. *t*-test: n.s., nonsignificant; $^*P < 0.05$; $^{***}P < 0.005$.

C Mean open time calculated from the estimated rate constant k_{01}. Data were calculated from the best-fit values, and errors are 95% confidence intervals. The number of replicates is as in (B). *t*-test: n.s., nonsignificant; $^{***}P < 0.005$.

D Instantaneous I-V relations of the indicated mutants at a saturating Ca^{2+} concentration. Data are averages of 6, 6, 7, and 13 patches for WT, L647V/I733V, I641A, and L647V/I733V/I641A respectively, and errors are SEM. Solid lines are fits to a model of ion permeation (Equation 1 in Supplementary Methods) shown in Fig 3B. Dashed line is the relation of wild-type.

E Conduction parameters for the indicated barriers. Data are best-fit values, and errors are 95% confidence intervals. Dashed line indicates the value of wild-type. The number of replicates is as in (D). *t*-test: $^{***}P < 0.005$.

F Coupling energies (ΔΔΔE) for the indicated barriers. Bars indicate quantities calculated using Equations 2 and 10 in Supplementary Methods, and errors are standard errors. The number of replicates is as in (D). *t*-test: $^{***}P < 0.005$.

G σ_β ratios of the indicated mutants on the wild-type or L647V/I733V background. Data are calculated from the best-fit values, and errors are 95% confidence intervals. Data were obtained from 6, 6, 7, and 10 patches for WT, I550A, I641A, and I550A/I641A respectively (Left) and from 10, 8, 13, and 9 patches for L647V/I733V, L647V/I733V/I550A, L647V/I733V/I641A, and L647V/I733V/I550A/I641A respectively (Right). *t*-test: n.s., nonsignificant; $^{***}P < 0.005$.

H Coupling energies (ΔΔΔE) for the inner barrier. Bars indicate quantities calculated using Equations 2 and 10 in Supplementary Methods, and errors are standard errors. The number of replicates is as in (G). *t*-test: $^{***}P < 0.005$.

Source data are available online for this figure.

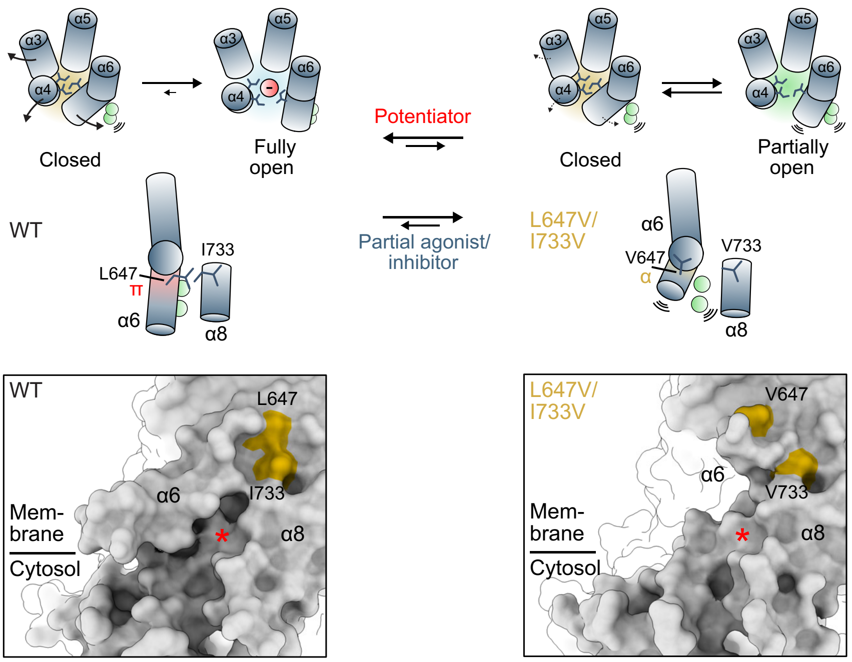

Figure 6. Mechanism and pharmacological implications.

Achieving maximum Po in the fully liganded state (i.e. at saturating Ca^{2+} concentrations) requires α6 in its fully activated conformation, which involves a transition into a π-helix that is stabilized by Ca^{2+} binding and the interaction between Leu 647 and Ile 733. This creates a local structural environment that favors a complete release of the inner pore gate, ensuring a full open probability and channel conductance. Stabilization of the activated conformation of α6 is expected to potentiate TMEM16A activity, while destabilizing α6 would result in partial agonism or inhibition, through modulation of both the gating equilibrium and channel conductance. Potential druggable region for rationally designed TMEM16A modulators is shown in the inset (red asterisk). Left, PDBID 7ZK3 (Lam *et al*, 2022).

channel opening (Figs 3 and 5). Our data, therefore, provide direct experimental evidence that binding site closure is linked to agonist efficacy via the conformation of α6 (Figs 6 and EV5).

Binding site closure upon ligand binding is a general phenomenon in LGICs, notably in pentameric neurotransmitter-gated channels (pLGICs) and ionotropic glutamate receptors (Du *et al*, 2015; Plested, 2016; Twomey & Sobolevsky, 2018; Yu *et al*, 2021). In TMEM16A, this process involves an agonist-coupled rearrangement of α6 that brings an essential glutamate (Glu 654) into direct contact with the bound Ca^{2+} (Paulino *et al*, 2017a). This movement is enabled by an α-to-π-helix transition below the conserved glycine hinge and is additionally stabilized by a hydrophobic

interaction between α6 and α8 in the π-helical region, mediated by Leu 647 and Ile 733 (Fig 1). Van der Waals attraction between these two residues, which are closely apposed, likely stabilizes the fully activated conformation of α6 as its rearrangement cannot be completed when their sidechains are truncated, with the ensuing portion of this helix becoming highly flexible (Fig 4). This primes and couples the α-to-π-helix transition and the binding of the second Ca^{2+}, both of which are subsequently stabilized by the Ca^{2+}-binding residues on α6, Asn 650 and Glu 654 (Fig 4). The straightened π-helical conformation of α6 likely relieves structural constraints in the gate region, thereby allowing a more extended opening and a prolongation of the open lifetime (Figs 2, 3 and 5).

The coupling between α6 rearrangement and the gate is likely bidirectional, as loosening the gate reciprocally enhances this agonist-promoted transition (Fig 5) and a direct disruption of the gate region results in an almost superimposable, partially activated conformation of α6 even in the absence of Ca^{2+} (Lam *et al*, 2021). A comparison of the available structures of TMEM16A with different open probabilities reveals a correlation between the degree of binding site closure and opening efficacy, with the π-helical conformation of α6 appearing to be central to this process (Fig EV5). The correspondence between incomplete binding site closure and impeded channel opening in L647V/I733V, where α6 rearrangement is impaired, and the previously described apo I551A (Lam *et al*, 2021), where the disruption of the gate results in a partial activation of α6 even in the absence of Ca^{2+} (Fig EV5A), suggests a general mechanism that governs agonist efficacy in TMEM16A. The immediate relevance of the tight interactions between α6 and α8 to channel opening highlights the combined molecular surface of both helices intracellular to the Leu 647/Ile 733 pair as a promising druggable region for the design of channel modulators (Fig 6), where molecules stabilizing this interaction would presumably potentiate channel activity at physiological Ca^{2+} concentrations. Conversely, destabilization of this conformation is likely to result in partial agonism/antagonism through a combined effect on both the gating equilibrium and channel conductance.

Agonist-bound intermediate closed states have been observed in several pLGICs, notably in nicotinic and glycine receptors (Lape *et al*, 2008; Mukhtasimova *et al*, 2009). These flipped or primed states display a higher affinity for the ligand, likely reflecting a rearrangement of the binding site. The ability of these channels to open in response to different ligands appears to be governed by the stability of these activated, pre-open states relative to that of the agonist-bound resting state, while the opening step remains equally efficacious irrespective of the efficacy of the ligands (Lape *et al*, 2008). Despite its distinct structural organization, channel opening in TMEM16A is also preceded by pre-open or flipped states that reflect the activation of α6 and the closure of the binding site; a conformational change that likely underlies the higher Ca^{2+} affinity in these states (Lam & Dutzler, 2021). By contrast, the relative stability of the open state in TMEM16A is dictated by the degree of binding site closure, with incomplete closure resulting in channels that open with lower probability (Figs 1, 2, 4 and EV5). It seems, therefore, that unlike the robustness of the ECD-TMD interface in pLGICs when bound to ligands with different efficacy (Yu *et al*, 2021; Ivica *et al*, 2022), the profound local structural changes between the closely apposed ligand binding and pore modules in TMEM16A may naturally give rise to a more obligatory coupling between binding site closure and channel opening.

In summary, our kinetic and structural analyses have allowed us to establish a relationship between agonist efficacy and binding site rearrangements in TMEM16A (Fig 6), a ligand-gated channel built on a distinct architecture that is conserved throughout the TMEM16 family. The coupling between channel opening and the closure of the binding site originates from the ability of Ca^{2+} to stabilize an otherwise energetically costly π-helical conformation to create a structural environment that favors channel opening. A related mechanism might underlie agonist binding and gating in TMEM16 scramblases, where more global conformational changes are propagated from the Ca^{2+}-binding site (Bushell *et al*, 2019; Falzone *et al*, 2019; Kalienkova *et al*, 2019; Arndt *et al*, 2022), and channels sharing a similar molecular scaffold such as the OSCA and TMC families of ion channels (Jojoa-Cruz *et al*, 2018; Zhang *et al*, 2018; Maity *et al*, 2019; Jeong *et al*, 2022). The structures of TMEM16A and its mutants with different efficacy, described here and previously, thus provide an important basis for the development of novel potentiators and partial agonists in the TMEM16 family.

Materials and Methods

Molecular biology and cell culture

HEK293T cells (ATCC CRL-1573) were maintained in Dulbecco's modified Eagle's medium (DMEM; Sigma-Aldrich) supplemented with 100 U/ml penicillin, 0.1 mg/ml streptomycin (Sigma-Aldrich), 2 mM L-glutamine (Sigma-Aldrich), and 10% FBS (Sigma-Aldrich) in a humidified atmosphere containing 5% CO_2 at 37°C. HEK293S GnTI$^-$ cells (ATCC CRL-3022) were maintained in HyClone HyCell TransFx-H medium (Cytiva) supplemented with 100 U/ml penicillin, 0.1 mg/ml streptomycin, 4 mM L-glutamine, 0.15% poloxamer 188 (Sigma-Aldrich), and 1% FBS in an atmosphere containing 5% CO_2 at 185 rpm at 37°C. The cell lines were obtained from commercial sources and further authentication was not performed. The cell lines were tested and are free from mycoplasma contamination. The *ac* splice variant of mouse TMEM16A (UniProt ID: Q8BHY3) bearing a 3C cleavage site, a Venus YFP, a Myc tag, and a Streptavidin-binding peptide (SBP) downstream of the open reading frame in a modified pcDNA3.1 vector (Invitrogen) was used as described previously (Lim *et al*, 2016). Mutations were introduced using a modified QuikChange method (Zheng *et al*, 2004) with primers described previously (Lam *et al*, 2021) and were verified by sequencing.

Protein expression and purification

HEK293S GnTI$^-$ cells were transiently transfected with mouse TMEM16A-L647V/I733V complexed with polyethylenimine MAX 40 K (formed in nonsupplemented DMEM medium at a w/w ratio of 1:2.5 for 30 min). Immediately after transfection, the culture was supplemented with 3.5 mM valproic acid. Cells were collected 48 h post-transfection, washed with PBS, and stored at −80°C until further use. Protein purification was carried out at 4°C and was completed within 12 h. The protein was purified in Ca^{2+}-free buffers and was supplemented with 1 mM free Ca^{2+} when indicated during cryo-EM sample preparation. Cells were resuspended and

 © 2023 The Authors

solubilized in 150 mM NaCl, 5 mM EGTA, 20 mM HEPES, 1x cOmplete protease inhibitors (Roche), 40 μg/ml DNase (AppliChem), and 2% GDN (Anatrace) at pH 7.4 by gentle mixing for 2 h. The solubilized fraction was obtained by centrifugation at 16,000 *g* for 30 min. After filtration with 0.5 μm filters (Sartorius), the supernatant was incubated with streptavidin UltraLink resin (Pierce, Thermo Fisher Scientific) for 2 h under gentle agitation. The beads were loaded onto a gravity column and were washed with 60 column volumes of SEC buffer containing 150 mM NaCl, 2 mM EGTA, 20 mM HEPES, 0.01% GDN at pH 7.4. The bound protein was eluted by incubating the beads with 3 column volumes of SEC buffer supplemented with 0.25 mg/ml 3C protease for 30 min. The eluate was concentrated using a 100 kDa cutoff filter, filtered through a 0.22 μm filter, and loaded onto a Superose 6 10/300 GL column (Cytiva) pre-equilibrated with SEC buffer. Peak fractions containing the protein were pooled, concentrated, and used immediately for cryo-EM sample preparation.

Cryo-EM sample preparation

2.5 μl of purified protein, concentrated to ~1.7 mg/ml and supplemented with 1 mM free Ca^{2+} immediately before plunge-freezing, was applied onto holey carbon grids (Quantifoil Au R1.2/1.3, 300 mesh) or holey gold grids (UltrAuFoil R1.2/1.3, 300 mesh) for the noncoated datasets collected at 0° and 20° tilt, respectively. Immediately prior to sample application, the grids were glow discharged at 15 mA for 30 s. After sample application, the grids were blotted for 2–4 s with a blot force setting of 0 at 4°C at 100% humidity, plunge-frozen in a liquid propane/ethane mixture using Vitrobot Mark IV (Thermo Fisher Scientific), and stored in liquid nitrogen until further use. For the dataset collected on graphene oxide (GO) support, samples were applied to the back side of UltrAuFoil grids deposited with GO (Sigma-Aldrich) on the front side at the Vitrobot according to (Cheung *et al*, 2018). In this case, the samples and grids were prepared as above except that the purified protein was used at ~0.5 mg/ml and that the grids were blotted for 2 s with a blot force setting of 7.

Cryo-EM data acquisition

Data collection was performed on a 300 kV Titan Krios G3i (Thermo Fisher Scientific) equipped with a post-column quantum energy filter (20 eV slit width) and a K3 summit direct electron detector (Gatan) in super-resolution mode. Dose-fractionated micrographs were collected at a nominal magnification of 130,000× corresponding to a pixel size of 0.659 Å/pixel (0.3295 Å/pixel in super-resolution) and a nominal defocus range of −1 to −2.4 μm using EPU 2.9 (Thermo Fisher Scientific). For datasets collected on noncoated grids at 0° and 20° tilt, each movie contained 36 frames with a total exposure time of 1 s and a total dose of approximately 66.1 e^{-} $Å^{-2}$ (1.85 e^{-} $Å^{-2}$ $frame^{-1}$). For datasets collected on GO support, each movie contained 47 frames and were binned two times on the fly, with a total exposure time of 1.26 s and a total dose of approximately 62.5 e^{-} $Å^{-2}$ (1.33 e^{-} $Å^{-2}$ $frame^{-1}$).

Cryo-EM data processing

The datasets were processed in RELION 3.1 (Zivanov *et al*, 2018). Micrographs were preprocessed using RELION's own implementation of MotionCor2 (Zheng *et al*, 2017), and Gctf (Zhang, 2016). crYOLO (Wagner *et al*, 2019) was used for automated particle picking, resulting in 896,659, 297,015, and 926,305 particle images, respectively, for the 0°, 20°, and GO datasets (6,924 (binned 2×), 7,167 (binned 2×), and 17,507 movies respectively). Particles were extracted with a box size of 480 pixels with 3× binning (160-pixel box, 1.977 Å/pixel) and were subjected to two to three rounds of 2D classification, separately for each dataset. Selected classes were pooled, resulting in 317,782 particles (126,229, 8,584, and 182,969 from the 0°, 20°, and GO datasets respectively) and were 3D-classified without symmetry applied using a previous Ca^{2+}-bound TMEM16A map low-pass filtered to 20 Å as a reference. Particles from the best classes (103,964, 6,242, and 34,728 from the 0°, 20°, and GO datasets respectively) were re-extracted with a box size of 400 pixels unbinned (0.659 Å/pixel) and were refined with C2 symmetry applied. A final map resolved to 3.29 Å was obtained after several rounds of CTF refinement and Bayesian polishing (separately for datasets with different electron dose), and a masked refinement excluding the detergent micelle upon convergence in the final refinement.

Model building, refinement, and validation

The initial model was obtained by fitting the previously determined Ca^{2+}-free TMEM16A-I551A structure (Lam *et al*, 2021) (PDBID 7B5D) into the density of the Ca^{2+}-bound TMEM16A-L647V/I733V using Chimera (Pettersen *et al*, 2004), which was then iteratively rebuilt in Coot (Emsley & Cowtan, 2004) and refined in Phenix (Afonine *et al*, 2018). The geometry of the final models was evaluated using MolProbity (Williams *et al*, 2018). Global and directional Fourier shell correlations (FSCs) between the half-maps were estimated using the 3DFSC server (https://3dfsc.salk.edu/) (Tan *et al*, 2017). Cross-validation was performed by evaluating FSC_{work}, the FSC between the final model with random shifts of up to 3 Å applied refined against one of the half-maps and this half-map, and FSC_{free}, the FSC between the resulting model and the other half-map. Figures containing molecular structures and maps were prepared using VMD (Humphrey *et al*, 1996) and ChimeraX (Pettersen *et al*, 2021). Angular difference of the principal axes of selected helices in superposed structures was calculated in VMD.

Electrophysiology

HEK293T cells were transfected with 3–4 μg DNA per 6 cm Petri dish using the calcium phosphate co-precipitation method and were used within 24–96 h after transfection. Recordings were performed on inside-out patches excised from HEK293T cells expressing the construct of interest. Patch pipettes were pulled from borosilicate glass capillaries (O.D. 1.5 mm, I.D. 0.86 mm, Sutter Instrument) and were fire-polished with a microforge (Narishige) before use. Pipette resistance was typically 3–8 MΩ when filled with the recording solutions detailed below. Seal resistance was typically 4 GΩ or higher. Voltage-clamp recordings were made using Axopatch 200B, Digidata 1550, and Clampex 10.7 (Molecular Devices). Analog signals were filtered with the in-built 4-pole Bessel filter at 10 kHz and were digitized at 20 kHz. Solution exchange was achieved using a gravity-fed system through a theta glass pipette mounted on an ultra-fast piezo-driven stepper (Siskiyou). Liquid junction potential was found to be consistently

© 2023 The Authors

negligible given the ionic composition of the solutions and was, therefore, not corrected. All recordings were performed at 20°C.

A symmetrical ionic condition was used throughout. Stock solution with Ca^{2+}-EGTA contained 150 mM NaCl, 5.99 mM $Ca(OH)_2$, 5 mM EGTA, and 10 mM HEPES at pH 7.40. Stock solution with EGTA contained 150 mM NaCl, 5 mM EGTA, and 10 mM HEPES at pH 7.40. Free Ca^{2+} concentrations were adjusted by mixing the stock solutions at the required ratios calculated using the WEBMAXC program (http://web.stanford.edu/~cpatton/webmaxcS.htm). Patch pipettes were filled with the stock solution with Ca^{2+}-EGTA, which has a free Ca^{2+} concentration of 1 mM. Unless otherwise stated, experiments were performed at a saturating Ca^{2+} concentration, and the primary data were corrected for current rundown as described previously (Lim *et al*, 2016; Lam & Dutzler, 2018). Analysis of the electrophysiology data is described in detail in the Supplementary Methods.

Data availability

Data supporting the findings of this study are available from the corresponding authors upon reasonable request. The cryo-EM map, half-maps, and mask have been deposited in the Electron Microscopy Data Bank under accession number EMD-18774 (http://www.ebi.ac.uk/pdbe/entry/EMD-18774). Coordinates for the model are available in the Protein Data Bank under PDB ID 8QZC (http://www.rcsb.org/pdb/explore/explore.do?structureId=8QZC). Source data are provided with this paper.

Expanded View for this article is available online.

Acknowledgements
This work was supported by a grant of the European Research Council (ERC no 339116, AnoBest) to RD and a Forschungskredit of the University of Zurich (grant no FK-18-048) and a Young Investigator Grant from the Novartis Foundation for Medical-Biological Research to AKML. The cryo-electron microscope and K3 camera were acquired with support of the Baugarten and Schwyzer-Winiker foundations and a Requip grant of the Swiss National Science Foundation. The Center for Microscopy and Image Analysis (ZMB) of the University of Zurich is acknowledged for their support and access to the electron microscope. We thank Marta Sawicka for help during cryo-EM data collection. All members of the Dutzler laboratory are acknowledged for their help at various stages of the project.

Author contributions
Andy KM Lam: Conceptualization; data curation; formal analysis; funding acquisition; validation; investigation; visualization; methodology; writing – original draft; writing – review and editing. **Raimund Dutzler:** Supervision; funding acquisition; project administration; writing – review and editing.

Disclosure and competing interests statement
The authors declare that they have no conflict of interest.

References

Afonine PV, Poon BK, Read RJ, Sobolev OV, Terwilliger TC, Urzhumtsev A, Adams PD (2018) Real-space refinement in PHENIX for cryo-EM and crystallography. *Acta Crystallogr D Struct Biol* 74: 531–544

Al-Hosni R, Ilkan Z, Agostinelli E, Tammaro P (2022) The pharmacology of the TMEM16A channel: therapeutic opportunities. *Trends Pharmacol Sci* 43: 712–725

Arndt M, Alvadia C, Straub MS, Clerico Mosina V, Paulino C, Dutzler R (2022) Structural basis for the activation of the lipid scramblase TMEM16F. *Nat Commun* 13: 6692

Arreola J, Hartzell HC (2019) Wasted TMEM16A channels are rescued by phosphatidylinositol 4,5-bisphosphate. *Cell Calcium* 84: 102103

Bushell SR, Pike ACW, Falzone ME, Rorsman NJG, Ta CM, Corey RA, Newport TD, Christianson JC, Scofano LF, Shintre CA *et al* (2019) The structural basis of lipid scrambling and inactivation in the endoplasmic reticulum scramblase TMEM16K. *Nat Commun* 10: 3956

Caputo A, Caci E, Ferrera L, Pedemonte N, Barsanti C, Sondo E, Pfeffer U, Ravazzolo R, Zegarra-Moran O, Galietta LJ (2008) TMEM16A, a membrane protein associated with calcium-dependent chloride channel activity. *Science* 322: 590–594

Cheung M, Adaniya H, Cassidy C, Yamashita M, Li KL, Taba S, Shintake T (2018) Improved sample dispersion in cryo-EM using "perpetually-hydrated" graphene oxide flakes. *J Struct Biol* 204: 75–79

Colquhoun D (1998) Binding, gating, affinity and efficacy: the interpretation of structure-activity relationships for agonists and of the effects of mutating receptors. *Br J Pharmacol* 125: 924–947

Danahay HL, Lilley S, Fox R, Charlton H, Sabater J, Button B, McCarthy C, Collingwood SP, Gosling M (2020) TMEM16A potentiation: a novel therapeutic approach for the treatment of cystic fibrosis. *Am J Respir Crit Care Med* 201: 946–954

Dang S, Feng S, Tien J, Peters CJ, Bulkley D, Lolicato M, Zhao J, Zuberbuhler K, Ye W, Qi L *et al* (2017) Cryo-EM structures of the TMEM16A calcium-activated chloride channel. *Nature* 552: 426–429

Dinsdale RL, Pipatpolkai T, Agostinelli E, Russell AJ, Stansfeld PJ, Tammaro P (2021) An outer-pore gate modulates the pharmacology of the TMEM16A channel. *Proc Natl Acad Sci USA* 118: e2023572118

Du J, Lu W, Wu S, Cheng Y, Gouaux E (2015) Glycine receptor mechanism elucidated by electron cryo-microscopy. *Nature* 526: 224–229

Emsley P, Cowtan K (2004) Coot: model-building tools for molecular graphics. *Acta Crystallogr D Biol Crystallogr* 60: 2126–2132

Falzone ME, Rheinberger J, Lee BC, Peyear T, Sasset L, Raczkowski AM, Eng ET, Di Lorenzo A, Andersen OS, Nimigean CM *et al* (2019) Structural basis of Ca^{2+}-dependent activation and lipid transport by a TMEM16 scramblase. *Elife* 8: e43229

Galietta LJV (2022) TMEM16A (ANO1) as a therapeutic target in cystic fibrosis. *Curr Opin Pharmacol* 64: 102206

Grosman C, Zhou M, Auerbach A (2000) Mapping the conformational wave of acetylcholine receptor channel gating. *Nature* 403: 773–776

Gupta S, Chakraborty S, Vij R, Auerbach A (2017) A mechanism for acetylcholine receptor gating based on structure, coupling, phi, and flip. *J Gen Physiol* 149: 85–103

Huang F, Zhang H, Wu M, Yang H, Kudo M, Peters CJ, Woodruff PG, Solberg OD, Donne ML, Huang X *et al* (2012) Calcium-activated chloride channel TMEM16A modulates mucin secretion and airway smooth muscle contraction. *Proc Natl Acad Sci USA* 109: 16354–16359

Humphrey W, Dalke A, Schulten K (1996) VMD: visual molecular dynamics. *J Mol Graph* 14: 27–38

Ivica J, Zhu H, Lape R, Gouaux E, Sivilotti LG (2022) Aminomethanesulfonic acid illuminates the boundary between full and partial agonists of the pentameric glycine receptor. *Elife* 11: e79148

Jadey S, Auerbach A (2012) An integrated catch-and-hold mechanism activates nicotinic acetylcholine receptors. *J Gen Physiol* 140: 17–28

 © 2023 The Authors

Jeng G, Aggarwal M, Yu WP, Chen TY (2016) Independent activation of distinct pores in dimeric TMEM16A channels. *J Gen Physiol* 148: 393–404

Jeong H, Clark S, Goehring A, Dehghani-Ghahnaviyeh S, Rasouli A, Tajkhorshid E, Gouaux E (2022) Structures of the TMC-1 complex illuminate mechanosensory transduction. *Nature* 610: 796–803

Jia Z, Chen J (2021) Specific PIP2 binding promotes calcium activation of TMEM16A chloride channels. *Commun Biol* 4: 259

Jojoa-Cruz S, Saotome K, Murthy SE, Tsui CCA, Sansom MS, Patapoutian A, Ward AB (2018) Cryo-EM structure of the mechanically activated ion channel OSCA1.2. *Elife* 7: e41845

Kalienkova V, Clerico Mosina V, Bryner L, Oostergetel GT, Dutzler R, Paulino C (2019) Stepwise activation mechanism of the scramblase nhTMEM16 revealed by cryo-EM. *Elife* 8: e44364

Lam AK, Dutzler R (2018) Calcium-dependent electrostatic control of anion access to the pore of the calcium-activated chloride channel TMEM16A. *Elife* 7: e39122

Lam AKM, Dutzler R (2021) Mechanism of pore opening in the calcium-activated chloride channel TMEM16A. *Nat Commun* 12: 786

Lam AKM, Rheinberger J, Paulino C, Dutzler R (2021) Gating the pore of the calcium-activated chloride channel TMEM16A. *Nat Commun* 12: 785

Lam AKM, Rutz S, Dutzler R (2022) Inhibition mechanism of the chloride channel TMEM16A by the pore blocker 1PBC. *Nat Commun* 13: 2798

Lape R, Colquhoun D, Sivilotti LG (2008) On the nature of partial agonism in the nicotinic receptor superfamily. *Nature* 454: 722–727

Le SC, Yang H (2020) An additional Ca^{2+} binding site allosterically controls TMEM16A activation. *Cell Rep* 33: 108570

Le SC, Jia Z, Chen J, Yang H (2019) Molecular basis of PIP2-dependent regulation of the Ca^{2+}-activated chloride channel TMEM16A. *Nat Commun* 10: 3769

Leffler JE (1953) Parameters for the description of transition states. *Science* 117: 340–341

Lim NK, Lam AK, Dutzler R (2016) Independent activation of ion conduction pores in the double-barreled calcium-activated chloride channel TMEM16A. *J Gen Physiol* 148: 375–392

Maity K, Heumann JM, McGrath AP, Kopcho NJ, Hsu PK, Lee CW, Mapes JH, Garza D, Krishnan S, Morgan GP *et al* (2019) Cryo-EM structure of OSCA1.2 from *Oryza sativa* elucidates the mechanical basis of potential membrane hyperosmolality gating. *Proc Natl Acad Sci USA* 116: 14309–14318

Mukhtasimova N, Lee WY, Wang HL, Sine SM (2009) Detection and trapping of intermediate states priming nicotinic receptor channel opening. *Nature* 459: 451–454

Paulino C, Kalienkova V, Lam AKM, Neldner Y, Dutzler R (2017a) Activation mechanism of the calcium-activated chloride channel TMEM16A revealed by cryo-EM. *Nature* 552: 421–425

Paulino C, Neldner Y, Lam AK, Kalienkova V, Brunner JD, Schenck S, Dutzler R (2017b) Structural basis for anion conduction in the calcium-activated chloride channel TMEM16A. *Elife* 6: e26232

Peters CJ, Yu H, Tien J, Jan YN, Li M, Jan LY (2015) Four basic residues critical for the ion selectivity and pore blocker sensitivity of TMEM16A calcium-activated chloride channels. *Proc Natl Acad Sci USA* 112: 3547–3552

Peters CJ, Gilchrist JM, Tien J, Bethel NP, Qi LJ, Chen TX, Wang L, Jan YN, Grabe M, Jan LY (2018) The sixth transmembrane segment is a major gating component of the TMEM16A calcium-activated chloride channel. *Neuron* 97: 1063–1077

Pettersen EF, Goddard TD, Huang CC, Couch GS, Greenblatt DM, Meng EC, Ferrin TE (2004) UCSF Chimera—a visualization system for exploratory research and analysis. *J Comput Chem* 25: 1605–1612

Pettersen EF, Goddard TD, Huang CC, Meng EC, Couch GS, Croll TI, Morris JH, Ferrin TE (2021) UCSF ChimeraX: structure visualization for researchers, educators, and developers. *Protein Sci* 30: 70–82

Plested AJ (2016) Structural mechanisms of activation and desensitization in neurotransmitter-gated ion channels. *Nat Struct Mol Biol* 23: 494–502

Schroeder BC, Cheng T, Jan YN, Jan LY (2008) Expression cloning of TMEM16A as a calcium-activated chloride channel subunit. *Cell* 134: 1019–1029

Sorum B, Czege D, Csanady L (2015) Timing of CFTR pore opening and structure of its transition state. *Cell* 163: 724–733

Ta CM, Acheson KE, Rorsman NJG, Jongkind RC, Tammaro P (2017) Contrasting effects of phosphatidylinositol 4,5-bisphosphate on cloned TMEM16A and TMEM16B channels. *Br J Pharmacol* 174: 2984–2999

Tan YZ, Baldwin PR, Davis JH, Williamson JR, Potter CS, Carragher B, Lyumkis D (2017) Addressing preferred specimen orientation in single-particle cryo-EM through tilting. *Nat Methods* 14: 793–796

Tembo M, Wozniak KL, Bainbridge RE, Carlson AE (2019) Phosphatidylinositol 4,5-bisphosphate (PIP2) and Ca^{2+} are both required to open the Cl^{-} channel TMEM16A. *J Biol Chem* 294: 12556–12564

Twomey EC, Sobolevsky AI (2018) Structural mechanisms of gating in ionotropic glutamate receptors. *Biochemistry* 57: 267–276

Wagner T, Merino F, Stabrin M, Moriya T, Antoni C, Apelbaum A, Hagel P, Sitsel O, Raisch T, Prumbaum D *et al* (2019) SPHIRE-crYOLO is a fast and accurate fully automated particle picker for cryo-EM. *Commun Biol* 2: 218

Williams CJ, Headd JJ, Moriarty NW, Prisant MG, Videau LL, Deis LN, Verma V, Keedy DA, Hintze BJ, Chen VB *et al* (2018) MolProbity: more and better reference data for improved all-atom structure validation. *Protein Sci* 27: 293–315

Yang YD, Cho H, Koo JY, Tak MH, Cho Y, Shim WS, Park SP, Lee J, Lee B, Kim BM *et al* (2008) TMEM16A confers receptor-activated calcium-dependent chloride conductance. *Nature* 455: 1210–1215

Ye W, Han TW, He M, Jan YN, Jan LY (2019) Dynamic change of electrostatic field in TMEM16F permeation pathway shifts its ion selectivity. *Elife* 8: e45187

Yu K, Jiang T, Cui Y, Tajkhorshid E, Hartzell HC (2019) A network of phosphatidylinositol 4,5-bisphosphate binding sites regulates gating of the Ca^{2+}-activated Cl^{-} channel ANO1 (TMEM16A). *Proc Natl Acad Sci USA* 116: 19952–19962

Yu J, Zhu H, Lape R, Greiner T, Du J, Lu W, Sivilotti L, Gouaux E (2021) Mechanism of gating and partial agonist action in the glycine receptor. *Cell* 184: 957–968

Zhang K (2016) Gctf: real-time CTF determination and correction. *J Struct Biol* 193: 1–12

Zhang M, Wang D, Kang Y, Wu JX, Yao F, Pan C, Yan Z, Song C, Chen L (2018) Structure of the mechanosensitive OSCA channels. *Nat Struct Mol Biol* 25: 850–858

Zheng L, Baumann U, Reymond JL (2004) An efficient one-step site-directed and site-saturation mutagenesis protocol. *Nucleic Acids Res* 32: e115

Zheng SQ, Palovcak E, Armache JP, Verba KA, Cheng Y, Agard DA (2017) MotionCor2: anisotropic correction of beam-induced motion for improved cryo-electron microscopy. *Nat Methods* 14: 331–332

Zivanov J, Nakane T, Forsberg BO, Kimanius D, Hagen WJ, Lindahl E, Scheres SH (2018) New tools for automated high-resolution cryo-EM structure determination in RELION-3. *Elife* 7: e42166

License: This is an open access article under the terms of the Creative Commons Attribution License, which permits use, distribution and reproduction in any medium, provided the original work is properly cited.

Expanded View Figures

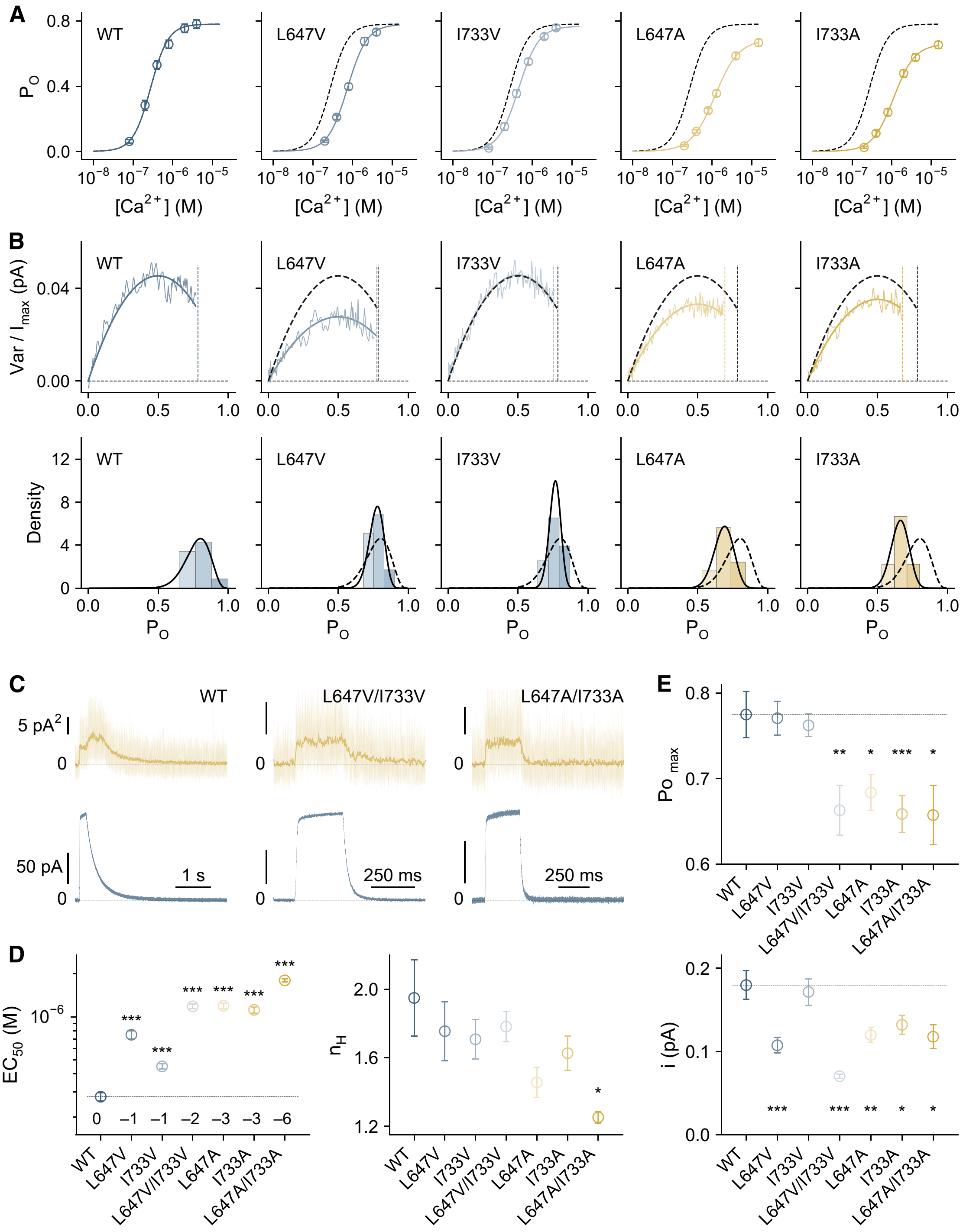

Figure EV1.

 © 2023 The Authors

 © 2023 The Authors

Figure EV1. Activation properties of mutants.

A Concentration-Po relations for the indicated mutants at +80 mV. Data are averages of 8, 7, 8, 8, and 8 patches for WT, L647V, I733V, L647A, and I733A respectively, and errors are SEM. Solid line is a fit to the Hill equation. Dashed line is the relation of wild-type.

B Top, merged and averaged variance-current relations at a saturating Ca^{2+} concentration at +80 mV. Data are averages of 11, 8, 10, 12, and 10 patches for WT, L647V, I733V, L647A, and I733A respectively. Solid line is a fit to Equation 5 in Supplementary Methods. Dashed line is the relation of wild-type. Dotted lines indicate the maximum Po. Bottom, histograms of the maximum Po obtained from individual measurements. Solid line is a fit to the beta distribution. Dashed line is the distribution of wild-type.

C Representative mean current and variance upon a step-exchange from zero to saturating Ca^{2+} and back. The raw variance is overlaid with its Gaussian moving average. Dashed lines indicate the zero current/variance levels.

D EC_{50} and n_H of the indicated constructs. Data are averages of the indicated number of patches shown in Appendix Table S1, and errors are SEM. The number of methyl groups truncated relative to wild-type is indicated in the left panel. WT, $n = 8$; L647V, $n = 7$; I733V, $n = 8$; L647V/I733V, $n = 8$; L647A, $n = 8$; I733A, $n = 8$; L647A/I733A, $n = 5$. t-test: *$P < 0.05$; ***$P < 0.005$.

E Po_{max} and i of the indicated constructs. Data are averages of the indicated number of patches shown in Appendix Table S2, and errors are SEM. WT, $n = 11$; L647V, $n = 8$; I733V, $n = 10$; L647V/I733V, $n = 20$; L647A, $n = 12$; I733A, $n = 10$; L647A/I733A, $n = 10$. t-test: *$P < 0.05$; **$P < 0.01$; ***$P < 0.005$.

Figure EV2. Cryo-EM reconstruction of Ca^{2+}-bound L647V/I733V.

A Representative micrographs (scale bar: 50 nm) and 2D class averages of L647V/I733V in the presence of Ca^{2+} for the indicated samples.

B Data processing workflow.

C Local resolution of the final map estimated using RELION.

D Angular distribution of particle projections used in the final refinement. Scale bar indicates the number of particle images.

E Half-map FSCs.

F Model-map FSCs.

G, H Sections of cryo-EM densities of (G) selected transmembrane helices and (H) the principal Ca^{2+}-binding site superimposed on the refined model.

© 2023 The Authors

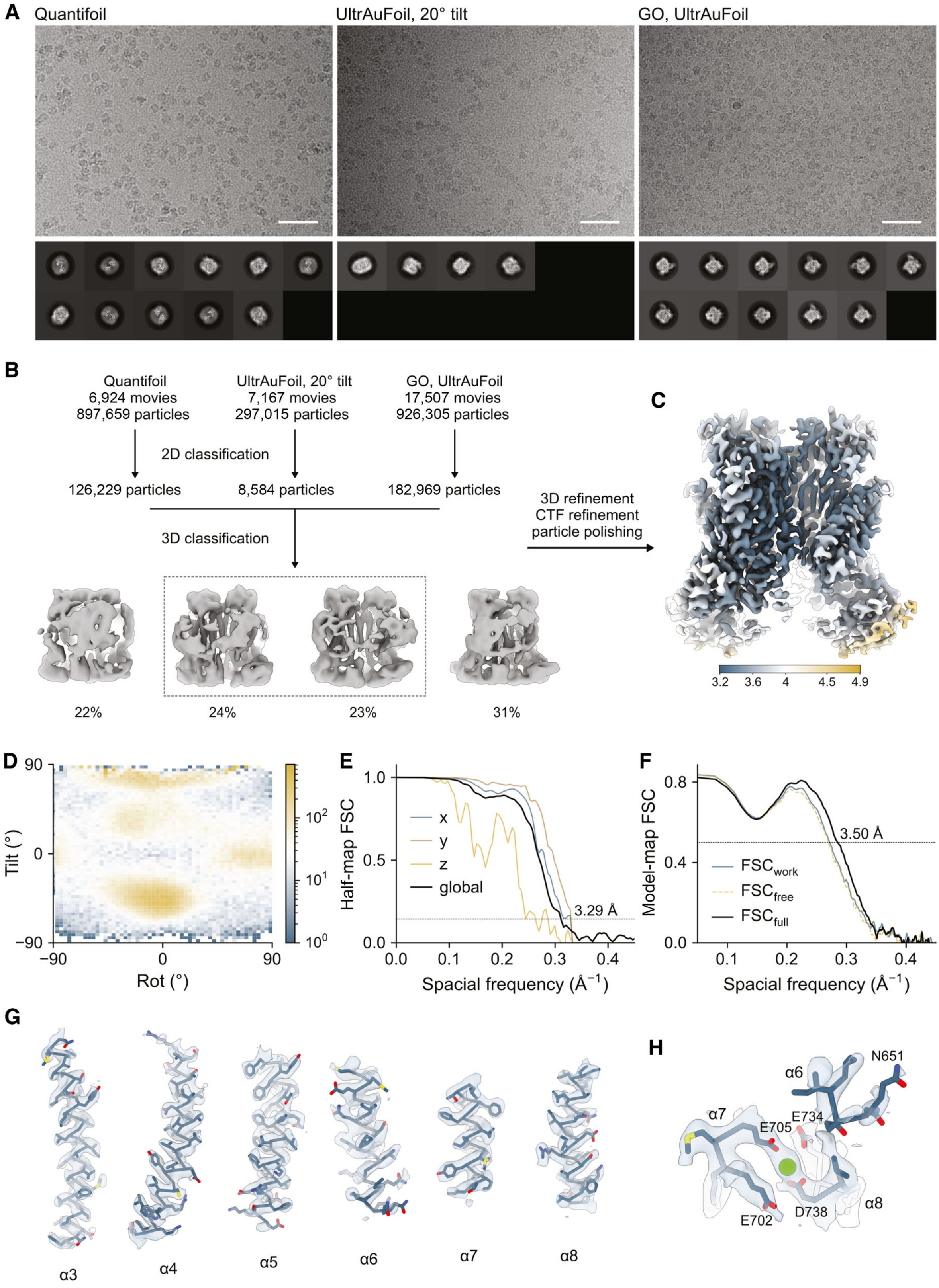

Figure EV2.

 © 2023 The Authors

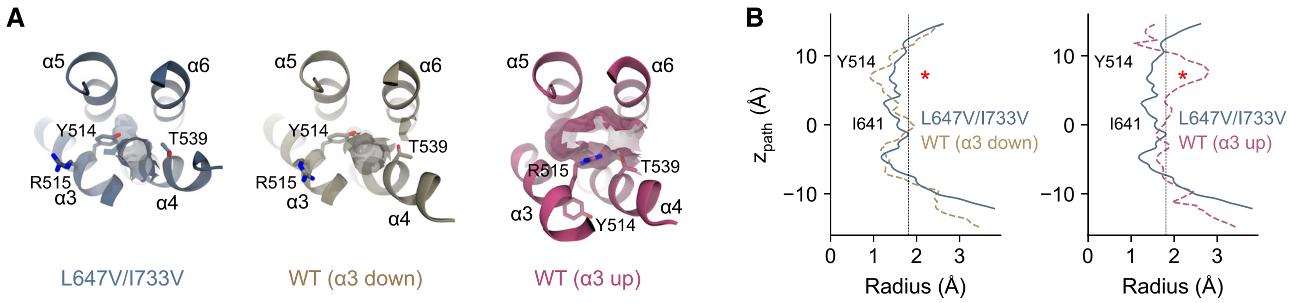

Figure EV3. Pore dimension.

A Molecular surface of the extracellular vestibule viewed from the top of the membrane. Selected residues lining the volume are shown. The bound 1PBC molecule in the WT (α3 up) model is omitted in the display for clarity. The models are as in Fig 4C.

B Pore radius along the z-axis relative to the position of Ile 641 (gate). The locations of constrictions are indicated. Asterisk indicates the location of the 1PBC-binding site. Dashed line denotes the ionic radius of a Cl^- ion.

© 2023 The Authors

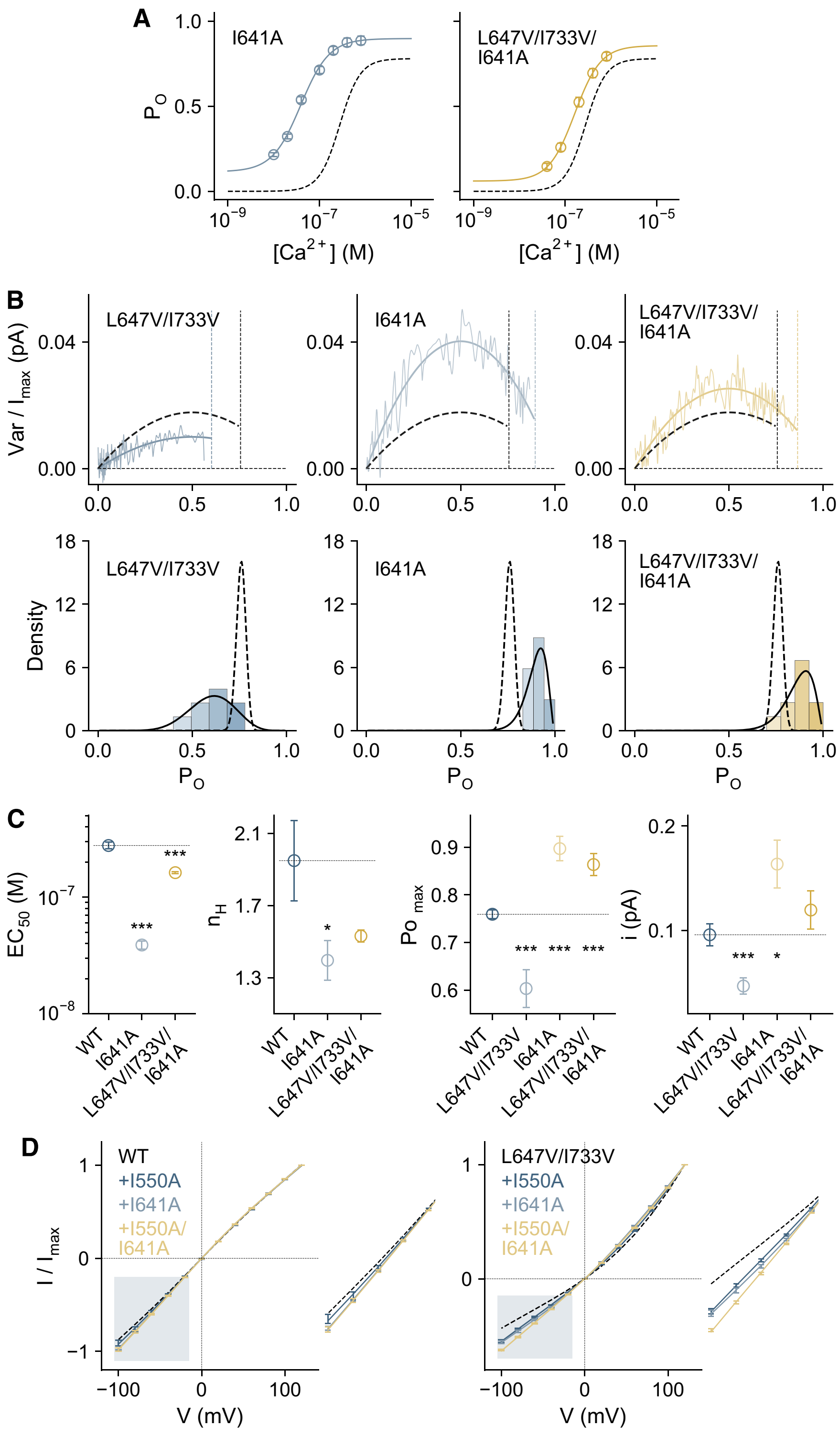

Figure EV4. Activation and conduction properties of mutants.

A Concentration-Po relations for the indicated mutants at +80 mV. Data are averages of 10 and 7 patches for I641A and L647V/I733V/I641A respectively, and errors are SEM. Solid line is a fit to the Hill equation. Dashed line is the relation of wild-type.

B Top, merged and averaged variance-current relations at a saturating Ca^{2+} concentration at −40 mV. Data are averages of 10, 6, and 10 patches for L647V/I733V, I641A, and L647V/I733V/I641A respectively. Solid line is a fit to Equation 5 in Supplementary Methods. Dashed line is the relation of wild-type. Dotted lines indicate the maximum Po. Bottom, histograms of the maximum Po obtained from individual measurements. Solid line is a fit to the beta distribution. Dashed line is the distribution of wild-type.

C EC_{50}, n_H, Po_{max}, and i of the indicated constructs. For EC_{50} and n_H, data are averages of 8, 10, and 7 patches for WT, I641A, and L647V/I733V/I641A respectively, and errors are SEM. For Po_{max} and i, data are averages of 8, 10, 6, and 10 patches for WT, L647V/I733V, I641A, and L647V/I733V/I641A respectively, and errors are SEM. *t*-test: $*P < 0.05$; $***P < 0.005$.

D Instantaneous I-V relations of the indicated mutants at a saturating Ca^{2+} concentration. Data are averages of 6, 6, 7, and 10 patches for WT, I550A, I641A, and I550A/I641A respectively (Left) and of 10, 8, 13, and 9 patches for L647V/I733V, L647V/I733V/I550A, L647V/I733V/I641A, and L647V/I733V/I550A/I641A respectively (Right), and errors are SEM. Solid lines are fits to a model of ion permeation (Equation 1 in Supplementary Methods) shown in Fig 3B. Dashed line is the relation of wild-type.

© 2023 The Authors

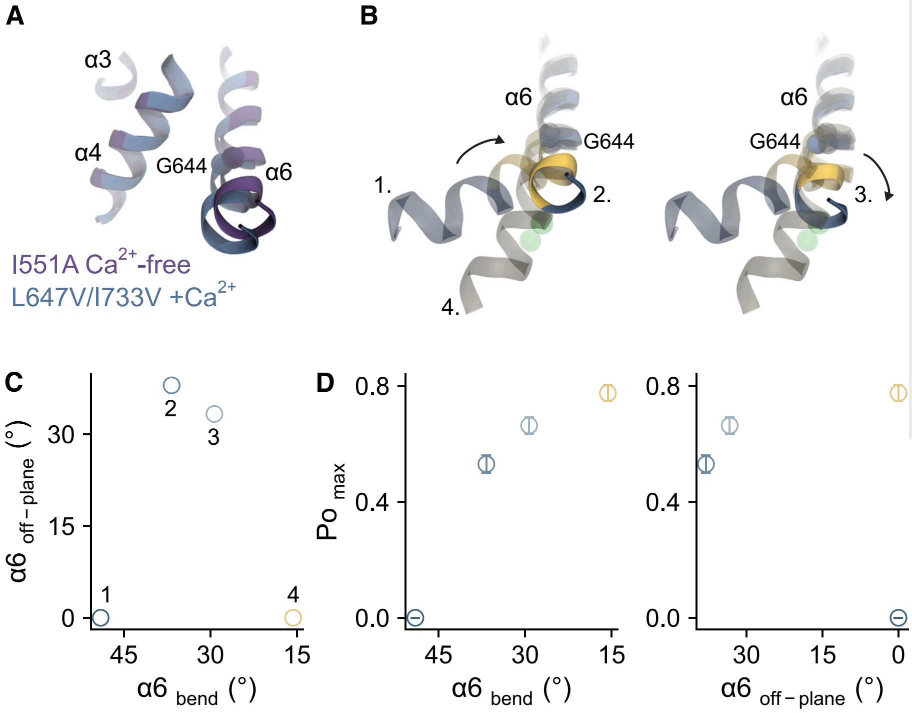

Figure EV5. α6 conformation and efficacy.

A Superposition of the I551A Ca^{2+}-free (PDBID 7B5D) and L647V/I733V + Ca^{2+} models. Selected helices are shown. The Cα of Gly 644 is shown as sphere.

B α6 conformations along its activation pathway. 1. WT Ca^{2+}-free (PDBID 5OYG), 2. I551A Ca^{2+}-free (PDBID 7B5D), 3. L647V/I733V + Ca^{2+}, and 4. WT + Ca^{2+} (PDBID 5OYB). The Cα of Gly 644 is shown as sphere. The bound Ca^{2+} in the wild-type model are shown as green spheres.

C Bending and off-plane movements below Gly 644 along the activation trajectory of α6. The plane defined by WT Ca^{2+}-free and WT + Ca^{2+} was used as a reference (0°).

D Relationship between efficacy and α6 conformations.

Appendix

Mechanistic basis of ligand efficacy in the calcium-activated chloride channel TMEM16A

Andy K. M. Lam and Raimund Dutzler

Includes:

Appendix Supplementary Methods

Appendix Figure S1-3

Appendix Table S1-4

Table of contents

	Page
Appendix Supplementary Methods	
Analysis of current-voltage (I-V) relations	3
Non-stationary noise analysis	4
Autocorrelation analysis	5
Double-mutant cycle analysis	7
Rate-equilibrium free-energy relation analysis	8
Statistics	8
Appendix Supplementary Figures	
Appendix Figure S1	10
Appendix Figure S2	12
Appendix Figure S3	13
Appendix Supplementary Tables	
Appendix Table S1	15
Appendix Table S2	15
Appendix Table S3	15
Appendix Table S4	16

Appendix Supplementary Methods

Analysis of current-voltage (I-V) relations

I-V data were fitted to a minimal permeation model that accounts for the fundamental biophysical behavior of mouse TMEM16A as described previously (Paulino *et al.*, 2017b),

$$I = zFAe^{\frac{zFV}{2nRT}} \frac{c_i - c_o e^{-\frac{zFV}{RT}}}{e^{-zFV\frac{n-1}{nRT}} + \left(\frac{1}{\sigma_h}\right)\frac{1 - e^{-zFV\frac{n-2}{nRT}}}{e^{\frac{zFV}{nRT}} - 1} + \frac{1}{\sigma_\beta}} \quad [1]$$

where I is the current, n is the number of barriers, c_i and c_o are the intracellular and extracellular concentrations of the charge carrier, z is the valence of Cl^-, V is the membrane voltage, and R, T, and F have their usual thermodynamic meanings. $A = \beta_0 v$ is a proportionality factor where β_0 is the value of β when $V = 0$ and v is a proportionality coefficient that has a dimension of volume. σ_h and σ_β are respectively the rate of barrier crossing at the middle and the innermost barriers relative to that at the outermost barrier (β). The best-fit values of σ_β and σ_h at a saturating Ca^{2+} concentration were used to calculate $\Delta E_{a\,(\sigma_\beta)}$ and $\Delta E_{a\,(\sigma_h)}$, the difference between the activation energy at the innermost barrier and the middle barrier relative to that of the outermost respectively, using

$$\Delta E_{a\,(\sigma_\beta)} = -RT\ln\sigma_\beta$$

$$\Delta E_{a\,(\sigma_h)} = -RT\ln\sigma_h \quad [2]$$

When analyzed on a unitary scale,

$$\beta_0/\beta_{0\mathrm{WT}} = \mathrm{A}/\mathrm{A}_{\mathrm{WT}}$$

$$\Delta E_{a\,(\beta)} = -RT\ln\frac{\beta_0}{\beta_{0\mathrm{WT}}} \quad [3]$$

were used to estimate the elevation of the outer barrier.

The half-maximum inhibition concentrations (IC_{50}) of 1PBC at the indicated voltages were obtained from steady-state I-V relations at a saturating Ca^{2+} concentration by fitting the concentration-response

relations to the Hill equation. The apparent valence of 1PBC (z_{block}) was estimated by fitting the voltage dependence of the IC50 in the exponential range (±40 mV) using

$$\mathrm{IC}_{50} = \mathrm{IC}_{50(0)} e^{\delta_b z_b VF/RT}$$

$$z_{\mathrm{block}} = \delta_b z_b \qquad [4]$$

where $\mathrm{IC}_{50(0)}$ is the IC50 in the absence of voltage, δ_b is the fraction of the transmembrane electric field operating on the blocker at its binding site, and z_b is the valence of the blocker.

Non-stationary noise analysis

Variance-current parabolas were calculated and analyzed as described previously (Lam & Dutzler, 2021). The current and variance were sampled by repeatedly activating and deactivating the channel using regularly spaced concentration jumps. The variance of such 50–100 aligned successive and kinetically identical currents at each time point was calculated by computing the mean of the squared successive difference (Heinemann & Conti, 1992), which mitigates the effect of non-stationarity at each isochrone and therefore allows the estimation of the variance in the presence of current rundown. The data were fitted to

$$\sigma_{\mathrm{total}}^2 = \sigma_N^2 + \sigma_{\mathrm{bg}}^2$$

$$\sigma_N^2 = i(\bar{I} - \bar{I}_{\mathrm{bg}}) - \frac{(\bar{I} - \bar{I}_{\mathrm{bg}})^2}{N} \qquad [5]$$

where σ_N^2 is the variance for N channels, i is the unitary current, $\bar{I}$ is the mean current, and the subscript bg denotes background. Data from different patches were merged by normalizing both x and y data according to the patch-specific parameter iN, the maximum achievable $\bar{I}$ for each patch if the Po was 1. Each data pair ($\bar{I}_j, \sigma_{N\,j}^2$) was sorted according to the $\bar{I}$ values and were averaged using a Gaussian moving average filter. The averaged data were re-fitted to Eq. 5 without the σ_{bg}^2 and $\bar{I}_{bg}$ terms. This procedure allows the estimated Po to be directly read from the merged σ_N^2-$\bar{I}$ plots.

Autocorrelation analysis

Power spectra were calculated and analyzed as described previously (Lam & Dutzler, 2021). Time series of steady-state currents of 50 or 100 s, recorded at a saturating Ca^{2+} concentration at +80 mV, were processed via Fast Fourier Transform (FFT) with a Hamming window applied to mitigate edge discontinuities. The data were digitized at a 20 kHz after passing through a 4-pole Bessel filter at 10 kHz. The background spectrum, recorded at 0 mV where the current reverses, was subtracted from the raw spectrum. The resulting spectrum was fitted to an empirical function consisting of a 1/f-like component, Lorentzian components, and a constant term,

$$P = \frac{a_0}{f^n} + \sum_i a_i \frac{1}{1 + \left(f/f_{c_i}\right)^2} + c \qquad [6]$$

where f is frequency, n is an exponent describing the decay, a_0 and a_i are respectively the amplitude of the 1/f-like and the Lorentzian components, f_{c_i} is the corner frequency, and c is a constant. A linear combination of three Lorentzian components was used, and the 1/f-like and the constant terms were subtracted from the background-subtracted spectrum, yielding a spectrum corresponding to fluctuations that reflect gating transitions.

The final spectrum was fitted to the following mechanism,

$$C_3 \leftrightarrow C_2 \leftrightarrow C_1 \leftrightarrow O_0$$

where C and O correspond to closed and open states respectively, and the subscripts denote the number assigned to the states. The matrix notation of this mechanism (Colquohoun & Hawkes, 1995) is

$$\boldsymbol{Q} = \begin{bmatrix} -k_{01} & k_{01} & 0 & 0 \\ k_{10} & -k_{10} - k_{12} & k_{12} & 0 \\ 0 & k_{21} & -k_{21} - k_{23} & k_{23} \\ 0 & 0 & k_{32} & -k_{32} \end{bmatrix}$$

where the subscripts indicate the transition described by the rate constant k in s^{-1}, for example k_{01} corresponds to the rate constant of the transition from state 0 to 1. k_{10} was calculated using

$$k_{10} = k_{01}(\frac{P_O}{1 - P_O})(\frac{1 + L_{32} + L_{32}L_{21}}{L_{32}L_{21}})$$

where L is the forward equilibrium constant with the subscript indicating the transition, and P_O was obtained from non-stationary noise analysis performed at a saturating Ca^{2+} concentration ($Po_{\max}$).

The equilibrium occupancy of states was calculated from (Colquohoun & Hawkes, 1995)

$$\boldsymbol{P}(\infty) = \boldsymbol{Y_0}(\boldsymbol{V}_{\lambda=0}\boldsymbol{V}^{-1}{}_{\lambda=0}) \qquad [7]$$

where $\boldsymbol{Y_0}$ is the initial occupancy and $\boldsymbol{V}$ can be obtained from the Eigen decomposition of $\boldsymbol{Q}$

$$\boldsymbol{Q} = \boldsymbol{V}\boldsymbol{\Lambda}\boldsymbol{V}^{-1}$$

and

$$\boldsymbol{\Lambda} = \begin{bmatrix} \lambda_1 & & \\ & \ddots & \\ & & \lambda_n \end{bmatrix}$$

$$\boldsymbol{V} = \begin{bmatrix} v_{11} & \cdots & v_{n1} \\ \vdots & \ddots & \vdots \\ v_{1n} & \cdots & v_{nn} \end{bmatrix}$$

are the Eigenvalue and Eigenvector matrices respectively. The corresponding spectral matrices are given by

$$\boldsymbol{A_i} = \boldsymbol{V}_{i^{th}col}\boldsymbol{V}^{-1}{}_{i^{th}row}$$

The data were fitted to the single-sided power spectrum due to Markovian fluctuations (Colquhoun & Hawkes, 1977)

$$G(f) = 4NV^2\boldsymbol{P_o}(\infty)\boldsymbol{\Gamma_o}\left[\sum_{i=2}^{n}\boldsymbol{A_{i_{oo}}}\frac{-\lambda_i^{-1}}{1+\left(\frac{2\pi f}{\lambda_i}\right)^2}\right]\boldsymbol{\Gamma_o}\boldsymbol{u_o} \qquad [8]$$

where N is the number of conducting units, V is the membrane potential,

$$\boldsymbol{P_o}(\infty) = \boldsymbol{P}(\infty)_{(o_1 \ldots o_k)}$$

is the steady-state occupancy of open states 1 to k,

$$\boldsymbol{A_{i_{oo}}} = \boldsymbol{A}_{\boldsymbol{i}(o_1 \ldots o_k,\, o_1 \ldots o_k)}$$

is a submatrix of the spectral matrix and $o_1 \ldots o_k,\ o_1 \ldots o_k$ denote the upper left elements,

$$\boldsymbol{\Gamma_o} = \begin{bmatrix} \gamma_{o_1} & & \\ & \ddots & \\ & & \gamma_{o_k} \end{bmatrix}$$

is the conductance of the states arranged in a matrix form, and

$$\boldsymbol{u_o} = \begin{bmatrix} 1 \\ \vdots \\ 1 \end{bmatrix}$$

is a unit vector of length corresponding to the number of open states. Because the amplitude of the power spectrum concerns the number of channels and their conductance, which are variables not related to mechanism, we fitted the experimental power spectra using a normalized form

$$G_{norm}(f) = \frac{G(f)}{G(0)} \qquad [9]$$

where $G(0)$ is a constant corresponding to the power at very low frequency. The parameters were estimated by minimizing the sum of squares.

The uniqueness in of the estimated parameters was evaluated by calculating the sum-of-square errors using the experimental spectrum as a function of each directly fitted rate constant with the other parameters fixed at their fitted values. The correlation between selected rate constants was evaluated by calculating the error surface on a two-dimensional grid with the remaining parameters fixed at their fitted values.

Double-mutant cycle analysis

The free energy of transition (ΔG) was calculated from the forward equilibrium constant using

$$\Delta G_{ij} = -RT \ln L_{ij}$$

where R and T have their usual thermodynamic meanings, L is the forward equilibrium constant and the subscript indicates the transition from state i to j. A double-mutant cycle (Carter *et al*, 1984; Horovitz, 1996) can be described by the following scheme

$$\begin{array}{ccccc} & & \Delta\Delta G_{ij}^{(0-X,Y)} & & \\ & X,Y & \rightarrow & 0,Y & \\ \Delta\Delta G_{ij}^{(X,0-Y)} & \downarrow & & \downarrow & \Delta\Delta G_{ij}^{(0,0-Y)} \\ & X,0 & \rightarrow & 0,0 & \\ & & \Delta\Delta G_{ij}^{(0-X,0)} & & \end{array}$$

where X and Y are two residues of interest and 0 denotes a mutation. The coupling energy between X and Y ($\Delta\Delta\Delta G^{XY}$) was calculated using either the X or Y mutations

$$\Delta\Delta\Delta G_{ij}^{XY} = \Delta\Delta G_{ij}^{(0-X,0)} - \Delta\Delta G_{ij}^{(0-X,Y)} = \left(\Delta G_{ij}^{(0,0)} - \Delta G_{ij}^{(X,0)}\right) - \left(\Delta G_{ij}^{(0,Y)} - \Delta G_{ij}^{(X,Y)}\right) \quad [10]$$

The standard error (σ) of the parameter estimates for each subtraction was propagated as described in the Statistics section. Deviation of $\Delta\Delta\Delta G_{ij}^{XY}$ from zero was detected using a one-sample t-test with a significance level of 0.05.

Rate-equilibrium free-energy relation analysis

The rate-equilibrium free-energy relation (Leffler, 1953; Leffler & Grunwald, 1963) consists of the following pair of relations

$$\begin{aligned} \log k_f &= \log k_i + \phi \log L \\ \log k_b &= \log k_i + (\phi - 1) \log L \end{aligned} \quad [11]$$

that describe the effect of a series of perturbations on the rate constants (k_f and k_b) as a fraction (ϕ and $\phi - 1$) of their effect on the forward equilibrium constant L. ϕ can adopt values between 0 and 1. k_i is the rate constant when L=1. The parameters ϕ and k_i were estimated by minimizing the total sum of squares for the set of equations for each transition.

Statistics

Data analysis was performed using Clampfit 10.7 (Molecular Devices), Excel (Microsoft), NumPy (https://numpy.org), and SciPy (https://scipy.org). For numerical calculations, NumPy and SciPy were

used. Parameter optimization was performed by minimizing the sum of squares using the least_squares function in SciPy. The variance of the best-fit parameters was obtained from the diagonal elements of the variance-covariance matrix (Colquhoun *et al*, 2003; van de Geer, 2005)

$$\boldsymbol{H}^{-1} = (\boldsymbol{J}^T \cdot \boldsymbol{J})^{-1}$$

multiplied by

$$\sigma^2 = \frac{\epsilon(\theta_{\text{est}})}{n_d - n_p}$$

where $\boldsymbol{H}$ and $\boldsymbol{J}$ are the Hessian and Jacobian matrices at the least squares estimates respectively, the superscript T indicates transpose, $\epsilon_G(\theta_{\text{est}})$ is the sum of squares given the estimated parameters, and n_d and n_p are the number of data points and parameters respectively. The square root of the variance was used to approximate the standard deviation error, from which the 95% confidence interval was calculated. Experimental data consisting of individual measurements are presented as mean ± SEM. Estimated parameters are presented as best-fit ± 95% confidence interval unless otherwise stated. Uncertainties were propagated using

$$\sigma_{(a+b \; or \; a-b)} = \sqrt{{\sigma_a}^2 + {\sigma_b}^2}$$

$$\frac{\sigma_{(ab \; or \; a/b)}}{|f(a,b)|} = \sqrt{\left(\frac{\sigma_a}{|a|}\right)^2 + \left(\frac{\sigma_b}{|b|}\right)^2}$$

The t-test, with a significance level of 0.05, was used for statistical comparison. No statistical methods were used to estimate the sample size. No blinding was performed.

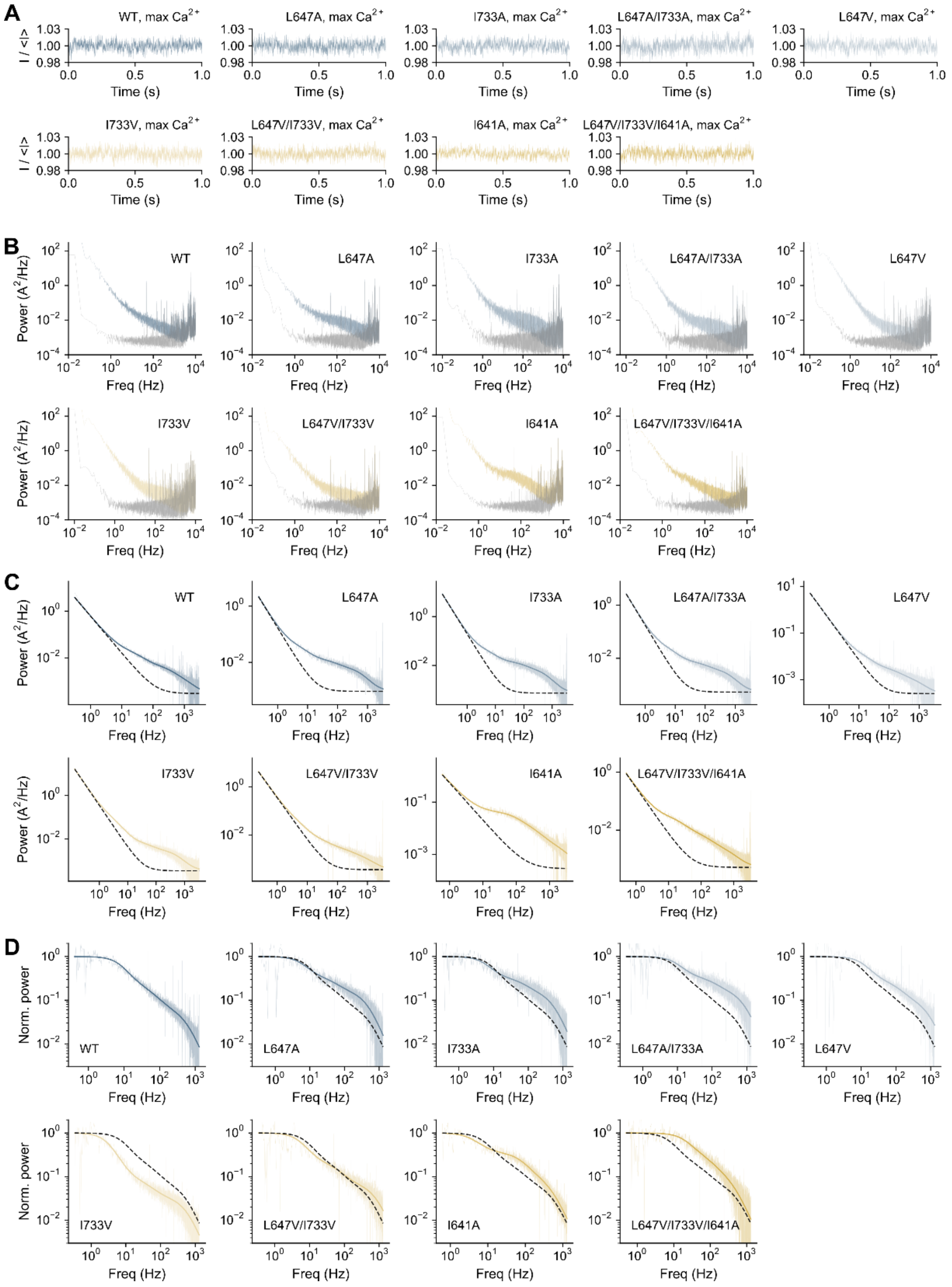

Appendix Figure S1. Autocorrelation analysis.

A. Representative section of steady-state current at a saturating Ca^{2+} concentration for the indicated mutants. For display, the traces were filtered at 2 kHz using a digital 4-pole Bessel lowpass filter.

B. Raw power spectra calculated from the steady-state currents shown in (A) and the corresponding background recorded at the reversal potential of Cl^- (grey).

C. Background-subtracted power spectra. Solid line is an empirical fit to Eq. 6 comprising the 1/f and constant components and components corresponding to fluctuations associated with channel gating. Dashed lines correspond to the estimated 1/f and constant components that are subtracted from the total spectrum to yield the gating component.

D. Normalized power spectra reflecting fluctuations due to channel gating. Solid line is a fit to Eqs. 7-9. Dashed line corresponds to the wild-type spectrum.

B-D. Data are averages of the indicated number of patches (WT, n=7; L647A, n=5; I733A, n=6; L647A/I733A, n=7; L647V, n=10; I733V, n=8; L647V/I733V, n=5; I641A, n=7; L647V/I733V/I641A, n=6).

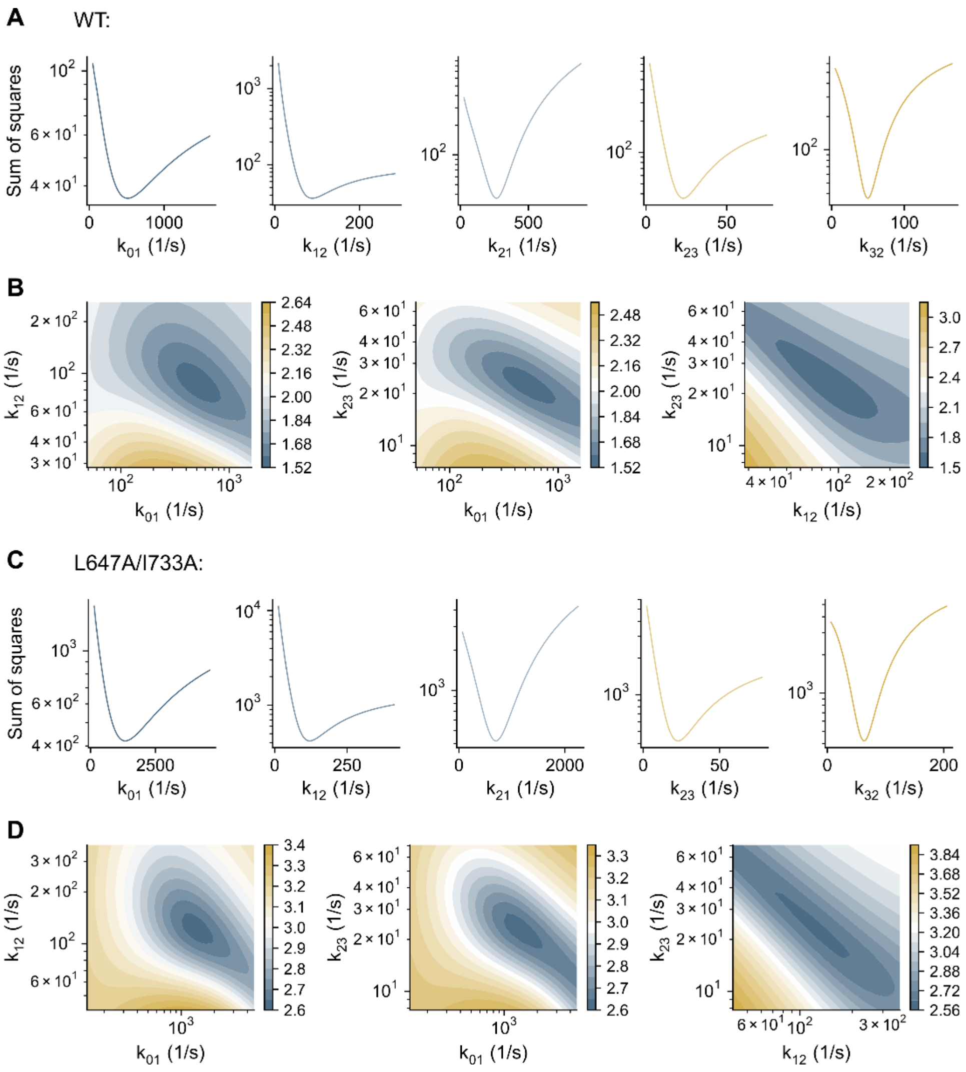

Appendix Figure S2. Estimatability of parameters from autocorrelation analysis.

A and C. Error function of the directly fitted rate constants of wild-type (A) and L647A/I733A (C). The errors were calculated using the corresponding experimental spectra with the other parameters fixed at their fitted values. A well-defined minimum is observed for each of the estimated parameters within the range consistent with the experimental characteristic frequencies.

B and D. Error surface of the backward rate constants of wild-type (B) and L647A/I733A (D). Plotted are the sum-of-squares errors calculated using the corresponding experimental spectra with the remaining three parameters fixed at their fitted values.

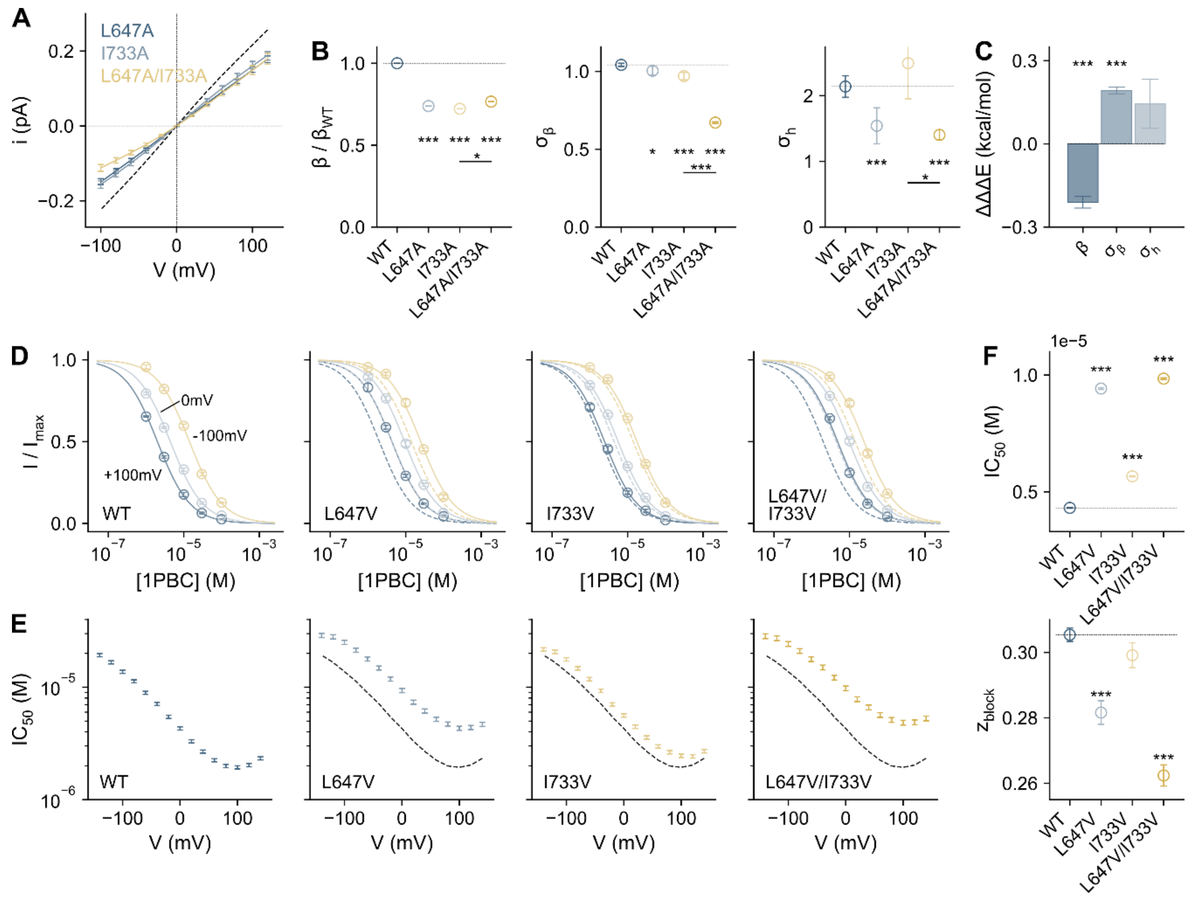

Appendix Figure S3. Pore properties of mutants.

A. Instantaneous current-voltage (I-V) relations of the indicated mutants at a saturating Ca^{2+} concentration on a unitary scale. Data were scaled according to the estimated unitary current from non-stationary noise analysis at +80 mV (Fig 1, Appendix Fig S1, and Appendix Table S2). Data are averages of 6, 7, 4, and 4 patches for WT, L647A, I733A, and L647A/I733A respectively, and errors are SEM. Solid lines are fits to a model of ion permeation (Eq. 1) shown in Fig 3B. Dashed line is the relation of wild-type.

B. Conduction parameters for the indicated barriers. Data are best-fit values, and errors are 95% confidence intervals. Dashed line indicates the value of wild-type. t-test: *, $p < 0.05$; ***, $p < 0.005$

C. Coupling energies (ΔΔΔE) for the indicated barriers. Bars indicate quantities calculated using Eqs. 2, 3, and 10, and errors are standard errors. t-test: ***, $p < 0.005$

D. Concentration-response relations of 1PBC at -100, 0, and 100 mV of the indicated mutants. Data are averages of 6, 6, 6, and 7 patches for WT, L647V, I733V, and L647V/I733V respectively, and errors are SEM. Solid lines are fits to the Hill equation. Dashed lines are the relations of wild-type.

E. Inhibition by 1PBC as a function of voltage for the indicated mutants. Data are best-fit values from a fit to the Hill equation using averaged concentration-response curves at the indicated voltages from the data shown in (D), and errors are 95% confidence intervals. Dashed line is the relation of wild-type.

F. IC_{50} at zero mV and z_{block} of the indicated constructs estimated by fitting the data in (E) using Eq. 4. Data are best-fit values, and errors are standard errors. t-test: ***, $p < 0.005$

Appendix Table S1. Concentration-response parameters at +80 mV

	EC_{50} (M)	SE	p-value	n_H	SE	p-value	No. of patches
**WT**	2.77e-07	1.83e-08	-	1.95	0.22	-	8
**L647V**	7.5E-07	4.91E-08	***	1.76	0.17	n.s.	7
**I733V**	4.51E-07	1.92E-08	***	1.71	0.11	n.s.	8
**L647V/I733V**	1.18E-06	4.25E-08	***	1.78	0.09	n.s.	8
**L647A**	1.2E-06	5.49E-08	***	1.46	0.09	n.s	8
**I733A**	1.12E-06	5.33E-08	***	1.63	0.10	n.s.	8
**L647A/I733A**	1.8E-06	4.43E-08	***	1.25	0.03	*	5

SE, standard error; n.s., non-significant; *, $p < 0.05$; ***, $p < 0.005$

Appendix Table S2. Noise analysis parameters at +80 mV

	$[Ca^{2+}]$	Po_{max}	SE	p-value	i (pA)	SE	p-value	No. of patches
**WT**	4 μM	0.775	0.027	-	0.18	0.017	-	11
**L647V**	15 μM	0.771	0.019	n.s.	0.107	0.009	***	8
**I733V**	4 μM	0.762	0.013	n.s.	0.172	0.016	n.s.	10
**L647V/I733V**	15 μM	0.663	0.029	**	0.07	0.003	***	20
**L647A**	15 μM	0.684	0.021	*	0.12	0.009	**	12
**I733A**	15 μM	0.659	0.022	***	0.132	0.011	*	10
**L647A/I733A**	50 μM	0.657	0.035	*	0.118	0.014	*	10

SE, standard error; n.s., non-significant; *, $p < 0.05$; **, $p < 0.01$; ***, $p < 0.005$

Appendix Table S3. Fitted gating parameters at saturating Ca^{2+} at +80 mV

	**WT**		**L647A**		**I733A**		**L647A/I733A**		**L647V**	
$[Ca^{2+}]$	4 μM		15 μM		15 μM		50 μM		15 μM	
	Best-fit	95% CI	Best-fit	95% CI	Best-fit	95% CI	Best-fit	95% CI	Best-fit	95% CI
k_{01} (s^{-1})	486.9	7.526	715.05	7.822	854.36	5.822	1398.4	17.406	846.5	32.564
k_{10} (s^{-1})	2593.6	221.10	2005.3	270.66	2258.7	159.58	3365.6	378.96	4052.6	836.07
k_{12} (s^{-1})	85.65	3.307	55.04	3.743	87.88	3.535	129.98	8.275	193.43	23.601
k_{21} (s^{-1})	267.31	8.842	328.35	16.68	474.07	11.913	704.76	28.311	740.96	58.7
k_{23} (s^{-1})	22.63	0.973	18.15	1.004	15.14	0.379	24.89	0.997	40	2.788
k_{32} (s^{-1})	50.25	0.856	41.61	1.079	39.16	0.468	64.77	1.243	83.45	2.551
L_{10}	5.33	0.462	2.8	0.38	2.64	0.188	2.41	0.273	4.79	1.005
L_{21}	3.12	0.159	5.97	0.506	5.39	0.256	5.42	0.408	3.83	0.556
L_{32}	2.22	0.103	2.29	0.14	2.59	0.072	2.6	0.116	2.09	0.159

	**I733V**		**L647V/I733V**		**I641A**		**L647V/I733V/ I641A**	
$[Ca^{2+}]$	4 μM		15 μM		2 μM		15 μM	
	Best-fit	95% CI	Best-fit	95% CI	Best-fit	95% CI	Best-fit	95% CI
k_{01} (s^{-1})	635.89	8.898	1320.0	44.532	219.28	2.403	203.98	6.344
k_{10} (s^{-1})	2669.1	250.76	3445.0	557.03	2552.3	137.06	2171.3	689.16
k_{12} (s^{-1})	51.98	2.959	101.7	9.106	199.15	6.024	234.89	44.638
k_{21} (s^{-1})	299.17	12.842	407.25	25.616	551.5	7.617	690.47	102.95
k_{23} (s^{-1})	26.21	0.607	18.01	1.069	4.53	0.105	108.72	11.579
k_{32} (s^{-1})	21.74	0.149	36.35	1.014	27.23	0.361	171.85	5.302
L_{10}	4.2	0.399	2.61	0.431	11.64	0.638	10.65	3.395
L_{21}	5.76	0.41	4	0.438	2.77	0.092	2.94	0.707

L_{32}	0.83	0.02	2.02	0.132	6.01	0.16	1.58	0.175

Normalized power spectra were fitted; CI, confidence interval.

Appendix Table S4. Fitted conduction parameters at saturating Ca^{2+}

	**A**		**σ_β**		**σ_h**	
	Best-fit	95% CI	Best-fit	95% CI	Best-fit	95% CI
**WT**	0.12	0.0007	1.042	0.0038	2.139	0.0592
**L647V**	0.108	0.0011	0.549	0.0034	0.727	0.0193
**I733V**	0.12	0.0008	0.965	0.0039	1.868	0.0548
**L647V/I733V**	0.11	0.001	0.308	0.002	0.343	0.0057
**L647A**	0.088	0.0013	1.004	0.0097	1.541	0.0913
**I733A**	0.086	0.0015	0.97	0.0105	2.494	0.2423
**L647A/I733A**	0.092	0.0006	0.671	0.0025	1.403	0.0334

Instantaneous currents on the absolute scale were fitted; CI, confidence interval.

Appendix

Mechanistic basis of ligand efficacy in the calcium-activated chloride channel TMEM16A

Andy K. M. Lam and Raimund Dutzler

Includes:

Appendix Supplementary Methods

Appendix Figure S1-3

Appendix Table S1-4

Table of contents

	Page
Appendix Supplementary Methods	
Analysis of current-voltage (I-V) relations	3
Non-stationary noise analysis	4
Autocorrelation analysis	5
Double-mutant cycle analysis	7
Rate-equilibrium free-energy relation analysis	8
Statistics	8
Appendix Supplementary Figures	
Appendix Figure S1	10
Appendix Figure S2	12
Appendix Figure S3	13
Appendix Supplementary Tables	
Appendix Table S1	15
Appendix Table S2	15
Appendix Table S3	15
Appendix Table S4	16

Appendix Supplementary Methods

Analysis of current-voltage (I-V) relations

I-V data were fitted to a minimal permeation model that accounts for the fundamental biophysical behavior of mouse TMEM16A as described previously (Paulino *et al.*, 2017b),

$$I = zFAe^{\frac{zFV}{2nRT}} \frac{c_i - c_o e^{-\frac{zFV}{RT}}}{e^{-zFV\frac{n-1}{nRT}} + \left(\frac{1}{\sigma_h}\right)\frac{1 - e^{-zFV\frac{n-2}{nRT}}}{e^{\frac{zFV}{nRT}} - 1} + \frac{1}{\sigma_\beta}} \qquad [1]$$

where I is the current, n is the number of barriers, c_i and c_o are the intracellular and extracellular concentrations of the charge carrier, z is the valence of Cl^-, V is the membrane voltage, and R, T, and F have their usual thermodynamic meanings. $A = \beta_0 v$ is a proportionality factor where β_0 is the value of β when $V = 0$ and v is a proportionality coefficient that has a dimension of volume. σ_h and σ_β are respectively the rate of barrier crossing at the middle and the innermost barriers relative to that at the outermost barrier (β). The best-fit values of σ_β and σ_h at a saturating Ca^{2+} concentration were used to calculate $\Delta E_{a\,(\sigma_\beta)}$ and $\Delta E_{a\,(\sigma_h)}$, the difference between the activation energy at the innermost barrier and the middle barrier relative to that of the outermost respectively, using

$$\Delta E_{a\,(\sigma_\beta)} = -RT\ln\sigma_\beta$$
$$\Delta E_{a\,(\sigma_h)} = -RT\ln\sigma_h \qquad [2]$$

When analyzed on a unitary scale,

$$\beta_0/\beta_{0\mathrm{WT}} = \mathrm{A}/\mathrm{A}_{\mathrm{WT}}$$
$$\Delta E_{a\,(\beta)} = -RT\ln\frac{\beta_0}{\beta_{0\mathrm{WT}}} \qquad [3]$$

were used to estimate the elevation of the outer barrier.

The half-maximum inhibition concentrations (IC_{50}) of 1PBC at the indicated voltages were obtained from steady-state I-V relations at a saturating Ca^{2+} concentration by fitting the concentration-response

relations to the Hill equation. The apparent valence of 1PBC (z_{block}) was estimated by fitting the voltage dependence of the IC_{50} in the exponential range (±40 mV) using

$$\text{IC}_{50} = \text{IC}_{50(0)} e^{\delta_b z_b VF/RT}$$
$$z_{\text{block}} = \delta_b z_b \qquad [4]$$

where $\text{IC}_{50(0)}$ is the IC_{50} in the absence of voltage, δ_b is the fraction of the transmembrane electric field operating on the blocker at its binding site, and z_b is the valence of the blocker.

Non-stationary noise analysis

Variance-current parabolas were calculated and analyzed as described previously (Lam & Dutzler, 2021). The current and variance were sampled by repeatedly activating and deactivating the channel using regularly spaced concentration jumps. The variance of such 50–100 aligned successive and kinetically identical currents at each time point was calculated by computing the mean of the squared successive difference (Heinemann & Conti, 1992), which mitigates the effect of non-stationarity at each isochrone and therefore allows the estimation of the variance in the presence of current rundown. The data were fitted to

$$\sigma_{\text{total}}^2 = \sigma_N^2 + \sigma_{\text{bg}}^2$$

$$\sigma_N^2 = i(\bar{I} - \bar{I}_{\text{bg}}) - \frac{(\bar{I} - \bar{I}_{\text{bg}})^2}{N} \qquad [5]$$

where σ_N^2 is the variance for N channels, i is the unitary current, $\bar{I}$ is the mean current, and the subscript bg denotes background. Data from different patches were merged by normalizing both x and y data according to the patch-specific parameter iN, the maximum achievable $\bar{I}$ for each patch if the Po was 1. Each data pair ($\bar{I}_j, \sigma_{N\,j}^2$) was sorted according to the $\bar{I}$ values and were averaged using a Gaussian moving average filter. The averaged data were re-fitted to Eq. 5 without the σ_{bg}^2 and $\bar{I}_{bg}$ terms. This procedure allows the estimated Po to be directly read from the merged σ_N^2-$\bar{I}$ plots.

Autocorrelation analysis

Power spectra were calculated and analyzed as described previously (Lam & Dutzler, 2021). Time series of steady-state currents of 50 or 100 s, recorded at a saturating Ca^{2+} concentration at +80 mV, were processed via Fast Fourier Transform (FFT) with a Hamming window applied to mitigate edge discontinuities. The data were digitized at a 20 kHz after passing through a 4-pole Bessel filter at 10 kHz. The background spectrum, recorded at 0 mV where the current reverses, was subtracted from the raw spectrum. The resulting spectrum was fitted to an empirical function consisting of a 1/f-like component, Lorentzian components, and a constant term,

$$P = \frac{a_0}{f^n} + \sum_i a_i \frac{1}{1 + \left(f/f_{c_i}\right)^2} + c \qquad [6]$$

where f is frequency, n is an exponent describing the decay, a_0 and a_i are respectively the amplitude of the 1/f-like and the Lorentzian components, f_{c_i} is the corner frequency, and c is a constant. A linear combination of three Lorentzian components was used, and the 1/f-like and the constant terms were subtracted from the background-subtracted spectrum, yielding a spectrum corresponding to fluctuations that reflect gating transitions.

The final spectrum was fitted to the following mechanism,

$$C_3 \leftrightarrow C_2 \leftrightarrow C_1 \leftrightarrow O_0$$

where C and O correspond to closed and open states respectively, and the subscripts denote the number assigned to the states. The matrix notation of this mechanism (Colquohoun & Hawkes, 1995) is

$$\boldsymbol{Q} = \begin{bmatrix} -k_{01} & k_{01} & 0 & 0 \\ k_{10} & -k_{10} - k_{12} & k_{12} & 0 \\ 0 & k_{21} & -k_{21} - k_{23} & k_{23} \\ 0 & 0 & k_{32} & -k_{32} \end{bmatrix}$$

where the subscripts indicate the transition described by the rate constant k in s^{-1}, for example k_{01} corresponds to the rate constant of the transition from state 0 to 1. k_{10} was calculated using

$$k_{10} = k_{01}\left(\frac{P_O}{1 - P_O}\right)\left(\frac{1 + L_{32} + L_{32}L_{21}}{L_{32}L_{21}}\right)$$

where L is the forward equilibrium constant with the subscript indicating the transition, and P_O was obtained from non-stationary noise analysis performed at a saturating Ca^{2+} concentration ($Po_{\max}$).

The equilibrium occupancy of states was calculated from (Colquohoun & Hawkes, 1995)

$$\boldsymbol{P}(\infty) = \boldsymbol{Y_0}(\boldsymbol{V}_{\lambda=0}\boldsymbol{V}^{-1}{}_{\lambda=0}) \quad [7]$$

where $\boldsymbol{Y_0}$ is the initial occupancy and $\boldsymbol{V}$ can be obtained from the Eigen decomposition of $\boldsymbol{Q}$

$$\boldsymbol{Q} = \boldsymbol{V\Lambda V}^{-1}$$

and

$$\boldsymbol{\Lambda} = \begin{bmatrix} \lambda_1 & & \\ & \ddots & \\ & & \lambda_n \end{bmatrix}$$

$$\boldsymbol{V} = \begin{bmatrix} v_{11} & \cdots & v_{n1} \\ \vdots & \ddots & \vdots \\ v_{1n} & \cdots & v_{nn} \end{bmatrix}$$

are the Eigenvalue and Eigenvector matrices respectively. The corresponding spectral matrices are given by

$$\boldsymbol{A_i} = \boldsymbol{V}_{i^{th}col}\boldsymbol{V}^{-1}{}_{i^{th}row}$$

The data were fitted to the single-sided power spectrum due to Markovian fluctuations (Colquhoun & Hawkes, 1977)

$$G(f) = 4NV^2\boldsymbol{P_o}(\infty)\boldsymbol{\Gamma_o}\left[\sum_{i=2}^{n}\boldsymbol{A_{i_{oo}}}\frac{-\lambda_i^{-1}}{1+\left(\frac{2\pi f}{\lambda_i}\right)^2}\right]\boldsymbol{\Gamma_o u_o} \quad [8]$$

where N is the number of conducting units, V is the membrane potential,

$$\boldsymbol{P_o}(\infty) = \boldsymbol{P}(\infty)_{(o_1 \dots o_k)}$$

is the steady-state occupancy of open states 1 to k,

$$\boldsymbol{A_{i_{oo}}} = \boldsymbol{A_i}_{(o_1 \dots o_k,\, o_1 \dots o_k)}$$

is a submatrix of the spectral matrix and $o_1 \dots o_k$, $o_1 \dots o_k$ denote the upper left elements,

$$\boldsymbol{\Gamma_o} = \begin{bmatrix} \gamma_{o_1} & & \\ & \ddots & \\ & & \gamma_{o_k} \end{bmatrix}$$

is the conductance of the states arranged in a matrix form, and

$$\boldsymbol{u_o} = \begin{bmatrix} 1 \\ \vdots \\ 1 \end{bmatrix}$$

is a unit vector of length corresponding to the number of open states. Because the amplitude of the power spectrum concerns the number of channels and their conductance, which are variables not related to mechanism, we fitted the experimental power spectra using a normalized form

$$G_{norm}(f) = \frac{G(f)}{G(0)} \qquad [9]$$

where $G(0)$ is a constant corresponding to the power at very low frequency. The parameters were estimated by minimizing the sum of squares.

The uniqueness in of the estimated parameters was evaluated by calculating the sum-of-square errors using the experimental spectrum as a function of each directly fitted rate constant with the other parameters fixed at their fitted values. The correlation between selected rate constants was evaluated by calculating the error surface on a two-dimensional grid with the remaining parameters fixed at their fitted values.

Double-mutant cycle analysis

The free energy of transition (ΔG) was calculated from the forward equilibrium constant using

$$\Delta G_{ij} = -RT \ln L_{ij}$$

where R and T have their usual thermodynamic meanings, L is the forward equilibrium constant and the subscript indicates the transition from state i to j. A double-mutant cycle (Carter *et al*, 1984; Horovitz, 1996) can be described by the following scheme

$$
\begin{array}{ccccc}
 & & \Delta\Delta G_{ij}^{(0-X,Y)} & & \\
 & X,Y & \rightarrow & 0,Y & \\
\Delta\Delta G_{ij}^{(X,0-Y)} & \downarrow & & \downarrow & \Delta\Delta G_{ij}^{(0,0-Y)} \\
 & X,0 & \rightarrow & 0,0 & \\
 & & \Delta\Delta G_{ij}^{(0-X,0)} & &
\end{array}
$$

where X and Y are two residues of interest and 0 denotes a mutation. The coupling energy between X and Y ($\Delta\Delta\Delta G^{XY}$) was calculated using either the X or Y mutations

$$\Delta\Delta\Delta G_{ij}^{XY} = \Delta\Delta G_{ij}^{(0-X,0)} - \Delta\Delta G_{ij}^{(0-X,Y)} = \left(\Delta G_{ij}^{(0,0)} - \Delta G_{ij}^{(X,0)}\right) - \left(\Delta G_{ij}^{(0,Y)} - \Delta G_{ij}^{(X,Y)}\right) \quad [10]$$

The standard error (σ) of the parameter estimates for each subtraction was propagated as described in the Statistics section. Deviation of $\Delta\Delta\Delta G_{ij}^{XY}$ from zero was detected using a one-sample t-test with a significance level of 0.05.

Rate-equilibrium free-energy relation analysis

The rate-equilibrium free-energy relation (Leffler, 1953; Leffler & Grunwald, 1963) consists of the following pair of relations

$$\log k_f = \log k_i + \phi \log L$$
$$\log k_b = \log k_i + (\phi - 1) \log L \quad [11]$$

that describe the effect of a series of perturbations on the rate constants (k_f and k_b) as a fraction (ϕ and $\phi - 1$) of their effect on the forward equilibrium constant L. ϕ can adopt values between 0 and 1. k_i is the rate constant when L=1. The parameters ϕ and k_i were estimated by minimizing the total sum of squares for the set of equations for each transition.

Statistics

Data analysis was performed using Clampfit 10.7 (Molecular Devices), Excel (Microsoft), NumPy (https://numpy.org), and SciPy (https://scipy.org). For numerical calculations, NumPy and SciPy were

used. Parameter optimization was performed by minimizing the sum of squares using the least_squares function in SciPy. The variance of the best-fit parameters was obtained from the diagonal elements of the variance-covariance matrix (Colquhoun *et al*, 2003; van de Geer, 2005)

$$\boldsymbol{H}^{-1} = (\boldsymbol{J}^{T} \cdot \boldsymbol{J})^{-1}$$

multiplied by

$$\sigma^2 = \frac{\epsilon(\theta_{\text{est}})}{n_d - n_p}$$

where $\boldsymbol{H}$ and $\boldsymbol{J}$ are the Hessian and Jacobian matrices at the least squares estimates respectively, the superscript T indicates transpose, $\epsilon_G(\theta_{\text{est}})$ is the sum of squares given the estimated parameters, and n_d and n_p are the number of data points and parameters respectively. The square root of the variance was used to approximate the standard deviation error, from which the 95% confidence interval was calculated. Experimental data consisting of individual measurements are presented as mean ± SEM. Estimated parameters are presented as best-fit ± 95% confidence interval unless otherwise stated. Uncertainties were propagated using

$$\sigma_{(a+b\ or\ a-b)} = \sqrt{{\sigma_a}^2 + {\sigma_b}^2}$$

$$\frac{\sigma_{(ab\ or\ a/b)}}{|f(a,b)|} = \sqrt{\left(\frac{\sigma_a}{|a|}\right)^2 + \left(\frac{\sigma_b}{|b|}\right)^2}$$

The t-test, with a significance level of 0.05, was used for statistical comparison. No statistical methods were used to estimate the sample size. No blinding was performed.

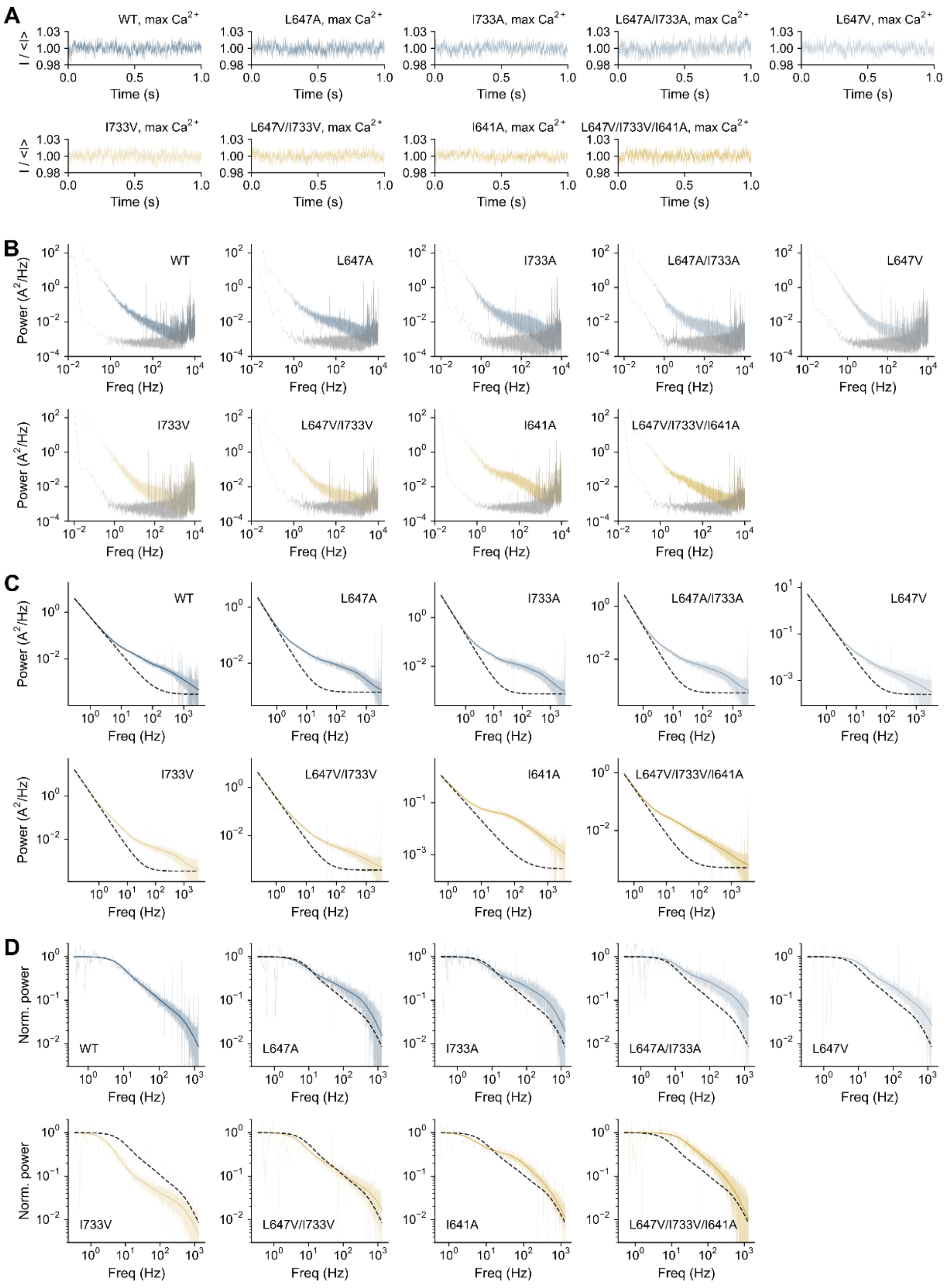

Appendix Figure S1. Autocorrelation analysis.

A. Representative section of steady-state current at a saturating Ca^{2+} concentration for the indicated mutants. For display, the traces were filtered at 2 kHz using a digital 4-pole Bessel lowpass filter.

B. Raw power spectra calculated from the steady-state currents shown in (A) and the corresponding background recorded at the reversal potential of Cl^- (grey).

C. Background-subtracted power spectra. Solid line is an empirical fit to Eq. 6 comprising the 1/f and constant components and components corresponding to fluctuations associated with channel gating. Dashed lines correspond to the estimated 1/f and constant components that are subtracted from the total spectrum to yield the gating component.

D. Normalized power spectra reflecting fluctuations due to channel gating. Solid line is a fit to Eqs. 7-9. Dashed line corresponds to the wild-type spectrum.

B-D. Data are averages of the indicated number of patches (WT, n=7; L647A, n=5; I733A, n=6; L647A/I733A, n=7; L647V, n=10; I733V, n=8; L647V/I733V, n=5; I641A, n=7; L647V/I733V/I641A, n=6).

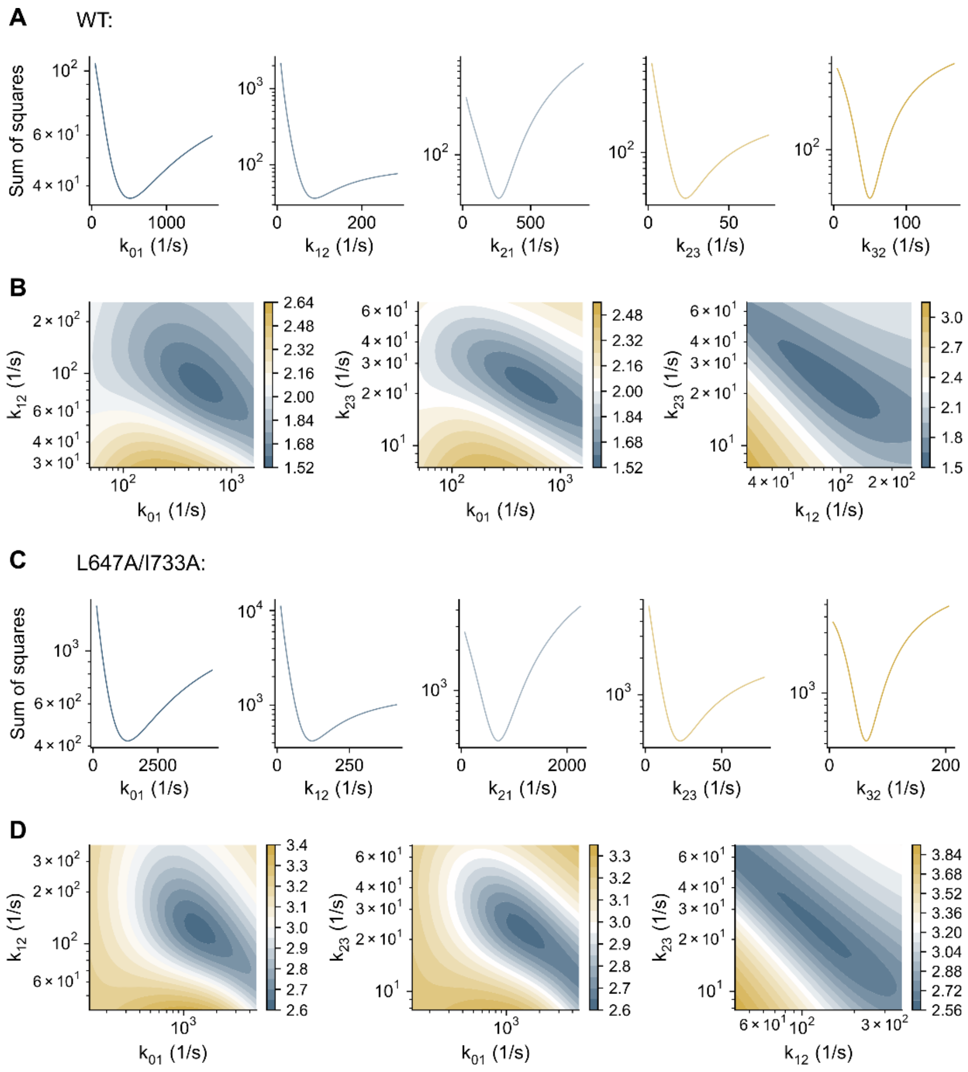

Appendix Figure S2. Estimatability of parameters from autocorrelation analysis.

A and C. Error function of the directly fitted rate constants of wild-type (A) and L647A/I733A (C). The errors were calculated using the corresponding experimental spectra with the other parameters fixed at their fitted values. A well-defined minimum is observed for each of the estimated parameters within the range consistent with the experimental characteristic frequencies.

B and D. Error surface of the backward rate constants of wild-type (B) and L647A/I733A (D). Plotted are the sum-of-squares errors calculated using the corresponding experimental spectra with the remaining three parameters fixed at their fitted values.

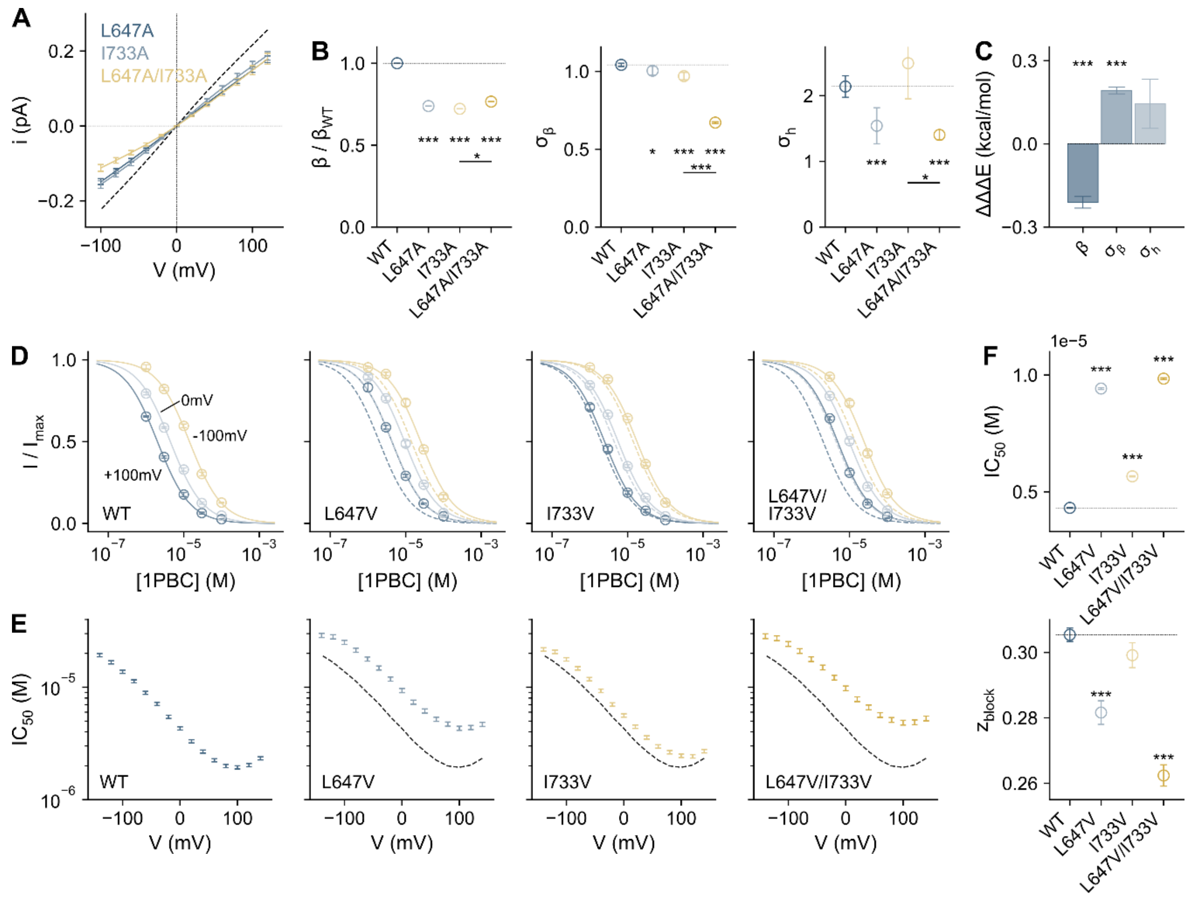

Appendix Figure S3. Pore properties of mutants.

A. Instantaneous current-voltage (I-V) relations of the indicated mutants at a saturating Ca^{2+} concentration on a unitary scale. Data were scaled according to the estimated unitary current from non-stationary noise analysis at +80 mV (Fig 1, Appendix Fig S1, and Appendix Table S2). Data are averages of 6, 7, 4, and 4 patches for WT, L647A, I733A, and L647A/I733A respectively, and errors are SEM. Solid lines are fits to a model of ion permeation (Eq. 1) shown in Fig 3B. Dashed line is the relation of wild-type.

B. Conduction parameters for the indicated barriers. Data are best-fit values, and errors are 95% confidence intervals. Dashed line indicates the value of wild-type. t-test: *, $p < 0.05$; ***, $p < 0.005$

C. Coupling energies (ΔΔΔE) for the indicated barriers. Bars indicate quantities calculated using Eqs. 2, 3, and 10, and errors are standard errors. t-test: ***, $p < 0.005$

D. Concentration-response relations of 1PBC at -100, 0, and 100 mV of the indicated mutants. Data are averages of 6, 6, 6, and 7 patches for WT, L647V, I733V, and L647V/I733V respectively, and errors are SEM. Solid lines are fits to the Hill equation. Dashed lines are the relations of wild-type.

E. Inhibition by 1PBC as a function of voltage for the indicated mutants. Data are best-fit values from a fit to the Hill equation using averaged concentration-response curves at the indicated voltages from the data shown in (D), and errors are 95% confidence intervals. Dashed line is the relation of wild-type.

F. IC_{50} at zero mV and z_{block} of the indicated constructs estimated by fitting the data in (E) using Eq. 4. Data are best-fit values, and errors are standard errors. t-test: ***, $p < 0.005$

Appendix Table S1. Concentration-response parameters at +80 mV

	EC_{50} (M)	SE	p-value	n_H	SE	p-value	No. of patches
**WT**	2.77e-07	1.83e-08	-	1.95	0.22	-	8
**L647V**	7.5E-07	4.91E-08	***	1.76	0.17	n.s.	7
**I733V**	4.51E-07	1.92E-08	***	1.71	0.11	n.s.	8
**L647V/I733V**	1.18E-06	4.25E-08	***	1.78	0.09	n.s.	8
**L647A**	1.2E-06	5.49E-08	***	1.46	0.09	n.s	8
**I733A**	1.12E-06	5.33E-08	***	1.63	0.10	n.s.	8
**L647A/I733A**	1.8E-06	4.43E-08	***	1.25	0.03	*	5

SE, standard error; n.s., non-significant; *, $p < 0.05$; ***, $p < 0.005$

Appendix Table S2. Noise analysis parameters at +80 mV

	[Ca^{2+}]	Po_{max}	SE	p-value	i (pA)	SE	p-value	No. of patches
**WT**	4 μM	0.775	0.027	-	0.18	0.017	-	11
**L647V**	15 μM	0.771	0.019	n.s.	0.107	0.009	***	8
**I733V**	4 μM	0.762	0.013	n.s.	0.172	0.016	n.s.	10
**L647V/I733V**	15 μM	0.663	0.029	**	0.07	0.003	***	20
**L647A**	15 μM	0.684	0.021	*	0.12	0.009	**	12
**I733A**	15 μM	0.659	0.022	***	0.132	0.011	*	10
**L647A/I733A**	50 μM	0.657	0.035	*	0.118	0.014	*	10

SE, standard error; n.s., non-significant; *, $p < 0.05$; **, $p < 0.01$; ***, $p < 0.005$

Appendix Table S3. Fitted gating parameters at saturating Ca^{2+} at +80 mV

	**WT**		**L647A**		**I733A**		**L647A/I733A**		**L647V**	
**[Ca^{2+}]**	4 μM		15 μM		15 μM		50 μM		15 μM	
	Best-fit	95% CI	Best-fit	95% CI	Best-fit	95% CI	Best-fit	95% CI	Best-fit	95% CI
**k_{01} (s^{-1})**	486.9	7.526	715.05	7.822	854.36	5.822	1398.4	17.406	846.5	32.564
**k_{10} (s^{-1})**	2593.6	221.10	2005.3	270.66	2258.7	159.58	3365.6	378.96	4052.6	836.07
**k_{12} (s^{-1})**	85.65	3.307	55.04	3.743	87.88	3.535	129.98	8.275	193.43	23.601
**k_{21} (s^{-1})**	267.31	8.842	328.35	16.68	474.07	11.913	704.76	28.311	740.96	58.7
**k_{23} (s^{-1})**	22.63	0.973	18.15	1.004	15.14	0.379	24.89	0.997	40	2.788
**k_{32} (s^{-1})**	50.25	0.856	41.61	1.079	39.16	0.468	64.77	1.243	83.45	2.551
**L_{10}**	5.33	0.462	2.8	0.38	2.64	0.188	2.41	0.273	4.79	1.005
**L_{21}**	3.12	0.159	5.97	0.506	5.39	0.256	5.42	0.408	3.83	0.556
**L_{32}**	2.22	0.103	2.29	0.14	2.59	0.072	2.6	0.116	2.09	0.159

	**I733V**		**L647V/I733V**		**I641A**		**L647V/I733V/ I641A**	
**[Ca^{2+}]**	4 μM		15 μM		2 μM		15 μM	
	Best-fit	95% CI	Best-fit	95% CI	Best-fit	95% CI	Best-fit	95% CI
**k_{01} (s^{-1})**	635.89	8.898	1320.0	44.532	219.28	2.403	203.98	6.344
**k_{10} (s^{-1})**	2669.1	250.76	3445.0	557.03	2552.3	137.06	2171.3	689.16
**k_{12} (s^{-1})**	51.98	2.959	101.7	9.106	199.15	6.024	234.89	44.638
**k_{21} (s^{-1})**	299.17	12.842	407.25	25.616	551.5	7.617	690.47	102.95
**k_{23} (s^{-1})**	26.21	0.607	18.01	1.069	4.53	0.105	108.72	11.579
**k_{32} (s^{-1})**	21.74	0.149	36.35	1.014	27.23	0.361	171.85	5.302
**L_{10}**	4.2	0.399	2.61	0.431	11.64	0.638	10.65	3.395
**L_{21}**	5.76	0.41	4	0.438	2.77	0.092	2.94	0.707

L_{32}	0.83	0.02	2.02	0.132	6.01	0.16	1.58	0.175

Normalized power spectra were fitted; CI, confidence interval.

Appendix Table S4. Fitted conduction parameters at saturating Ca^{2+}

	**A**		**σ_β**		**σ_h**	
	Best-fit	95% CI	Best-fit	95% CI	Best-fit	95% CI
**WT**	0.12	0.0007	1.042	0.0038	2.139	0.0592
**L647V**	0.108	0.0011	0.549	0.0034	0.727	0.0193
**I733V**	0.12	0.0008	0.965	0.0039	1.868	0.0548
**L647V/I733V**	0.11	0.001	0.308	0.002	0.343	0.0057
**L647A**	0.088	0.0013	1.004	0.0097	1.541	0.0913
**I733A**	0.086	0.0015	0.97	0.0105	2.494	0.2423
**L647A/I733A**	0.092	0.0006	0.671	0.0025	1.403	0.0334

Instantaneous currents on the absolute scale were fitted; CI, confidence interval.
