# Supplementary material for: Mechanistic basis of ligand efficacy in the calcium‐activated chloride channel TMEM16A
Source: EMBO J. 2023 Nov 20;42(24):e115030. doi: 10.15252/embj.2023115030 (PMC10711664; doi:10.15252/embj.2023115030)
Supplement: Supplementary file 8 [file EMBJ-42-e115030-s003.pdf]

THE EMBO JOURNAL

Mechanistic basis of ligand efficacy in the calcium-activated chloride channel TMEM16A

Andy Lam and Raimund Dutzler
DOI: 10.15252/embj.2023115030

Corresponding author(s): Raimund Dutzler (dutzler@bioc.uzh.ch) , Andy Lam (a.lam@bioc.uzh.ch)

Review Timeline:

Submission Date:	19th Jul 23
Editorial Decision:	28th Aug 23
Revision Received:	12th Oct 23
Editorial Decision:	24th Oct 23
Revision Received:	27th Oct 23
Accepted:	2nd Nov 23

Editor: William Teale

Transaction Report:

(Note: With the exception of the correction of typographical or spelling errors that could be a source of ambiguity, letters and reports are not edited. Depending on transfer agreements, referee reports obtained elsewhere may or may not be included in this compilation. Referee reports are anonymous unless the Referee chooses to sign their reports.)

Dear Raimund,

Thank you again for the submission of your manuscript entitled "Structural determinants of ligand efficacy in the calcium-activated chloride channel TMEM16A". We have now received the reports from two referees, which I have copied to the bottom of this message.

As you can see from their comments, both referees appreciate the significance of the mechanism of TMEM16A channel gating that you describe and are generally supportive of publication. Therefore, based on this overall interest, I would like to invite you to address the comments of all referees in a revised version of the manuscript. As you will see, Referee 1 raises some queries which will need to be addressed and Referee 2 has concerns about the general readability of the manuscript. If you would like to get my input on this, I would be happy to have a chat over Zoom.

I should add that it is The EMBO Journal policy to allow only a single major round of revision and that it is therefore important to resolve these concerns at this stage.

I would also like to point out that as a matter of policy, competing manuscripts published during this period will not be taken into consideration in our assessment of the novelty presented by your study ("scooping" protection). We have extended this 'scooping protection policy' beyond the usual 3 month revision timeline to cover the period required for a full revision to address the essential experimental issues. Please contact me if you see a paper with related content published elsewhere to discuss the appropriate course of action.

When preparing your letter of response to the referees' comments, please bear in mind that this will form part of the Review Process File, and will therefore be available online to the community. For more details on our Transparent Editorial Process, please visit our website: https://www.embopress.org/page/journal/14602075/authorguide#transparentprocess

Again, please contact me at any time during revision if you need any help or have further questions.

Thank you very much again for the opportunity to consider your work for publication. I look forward to your revision.

Best regards,

Will

William Teale, Ph.D.
Editor
The EMBO Journal

When submitting your revised manuscript, please carefully review the instructions below and include the following items:

1) a .docx formatted version of the manuscript text (including legends for main figures, EV figures and tables). Please make sure that the changes are highlighted to be clearly visible.

2) individual production quality figure files as .eps, .tif, .jpg (one file per figure).

3) a .docx formatted letter INCLUDING the reviewers' reports and your detailed point-by-point response to their comments. As part of the EMBO Press transparent editorial process, the point-by-point response is part of the Review Process File (RPF), which will be published alongside your paper.

4) a complete author checklist, which you can download from our author guidelines (https://wol-prod-cdn.literatumonline.com/pb-assets/embo-site/Author Checklist%20-%20EMBO%20J-1561436015657.xlsx). Please insert information in the checklist that is also reflected in the manuscript. The completed author checklist will also be part of the RPF.

5) Please note that all corresponding authors are required to supply an ORCID ID for their name upon submission of a revised manuscript.

6) We require a 'Data Availability' section after the Materials and Methods. Before submitting your revision, primary datasets produced in this study need to be deposited in an appropriate public database, and the accession numbers and database listed under 'Data Availability'. Please remember to provide a reviewer password if the datasets are not yet public (see https://www.embopress.org/page/journal/14602075/authorguide#datadeposition). If no data deposition in external databases is needed for this paper, please then state in this section: This study includes no data deposited in external repositories. Note that

the Data Availability Section is restricted to new primary data that are part of this study.

Note - All links should resolve to a page where the data can be accessed.

7) When assembling figures, please refer to our figure preparation guideline in order to ensure proper formatting and readability in print as well as on screen:
http://bit.ly/EMBOPressFigurePreparationGuideline

Please remember: Digital image enhancement is acceptable practice, as long as it accurately represents the original data and conforms to community standards. If a figure has been subjected to significant electronic manipulation, this must be noted in the figure legend or in the 'Materials and Methods' section. The editors reserve the right to request original versions of figures and the original images that were used to assemble the figure.

8) For data quantification: please specify the name of the statistical test used to generate error bars and P values, the number (n) of independent experiments (specify technical or biological replicates) underlying each data point and the test used to calculate p-values in each figure legend. The figure legends should contain a basic description of n, P and the test applied. Graphs must include a description of the bars and the error bars (s.d., s.e.m.).

9) We would also encourage you to include the source data for figure panels that show essential data. Numerical data can be provided as individual .xls or .csv files (including a tab describing the data). For 'blots' or microscopy, uncropped images should be submitted (using a zip archive or a single pdf per main figure if multiple images need to be supplied for one panel). Additional information on source data and instruction on how to label the files are available at .

10) We replaced Supplementary Information with Expanded View (EV) Figures and Tables that are collapsible/expandable online (see examples in https://www.embopress.org/doi/10.15252/embj.201695874). A maximum of 5 EV Figures can be typeset. EV Figures should be cited as 'Figure EV1, Figure EV2" etc. in the text and their respective legends should be included in the main text after the legends of regular figures.

- For the figures that you do NOT wish to display as Expanded View figures, they should be bundled together with their legends in a single PDF file called *Appendix*, which should start with a short Table of Content. Appendix figures should be referred to in the main text as: "Appendix Figure S1, Appendix Figure S2" etc. See detailed instructions regarding expanded view here: .

- Additional Tables/Datasets should be labelled and referred to as Table EV1, Dataset EV1, etc. Legends have to be provided in a separate tab in case of .xls files. Alternatively, the legend can be supplied as a separate text file (README) and zipped together with the Table/Dataset file.

11) At EMBO Press we ask authors to provide source data for the main manuscript figures. Our source data coordinator will contact you to discuss which figure panels we would need source data for and will also provide you with helpful tips on how to upload and organize the files.

12) Our journal encourages inclusion of *data citations in the reference list* to directly cite datasets that were re-used and obtained from public databases. Data citations in the article text are distinct from normal bibliographical citations and should directly link to the database records from which the data can be accessed. In the main text, data citations are formatted as follows: "Data ref: Smith et al, 2001" or "Data ref: NCBI Sequence Read Archive PRJNA342805, 2017". In the Reference list, data citations must be labeled with "[DATASET]". A data reference must provide the database name, accession number/identifiers and a resolvable link to the landing page from which the data can be accessed at the end of the reference. Further instructions are available at .

Further instructions for preparing your revised manuscript:

Please make sure you upload a letter of response to the referees' comments together with the revised manuscript.

Please also check that the title and abstract of the manuscript are brief, yet explicit, even to non-specialists.

When assembling figures, please refer to our figure preparation guideline in order to ensure proper formatting and readability in print as well as on screen:
https://bit.ly/EMBOPressFigurePreparationGuideline
See also guidelines for figure legends: https://www.embopress.org/page/journal/14602075/authorguide#figureformat

At EMBO Press we ask authors to provide source data for the main manuscript figures. Our source data coordinator will contact you to discuss which figure panels we would need source data for and will also provide you with helpful tips on how to upload and organize the files.

IMPORTANT: When you send the revision we will require

- a point-by-point response to the referees' comments, with a detailed description of the changes made (as a word file).
- a word file of the manuscript text.
- individual production quality figure files (one file per figure)
- a complete author checklist, which you can download from our author guidelines (https://www.embopress.org/page/journal/14602075/authorguide).
- Expanded View files (replacing Supplementary Information)
Please see out instructions to authors
https://www.embopress.org/page/journal/14602075/authorguide#expandedview

Please remember: Digital image enhancement is acceptable practice, as long as it accurately represents the original data and conforms to community standards. If a figure has been subjected to significant electronic manipulation, this must be noted in the figure legend or in the 'Materials and Methods' section. The editors reserve the right to request original versions of figures and the original images that were used to assemble the figure.

Further information is available in our Guide For Authors: https://www.embopress.org/page/journal/14602075/authorguide

We realize that it is difficult to revise to a specific deadline. In the interest of protecting the conceptual advance provided by the work, we recommend a revision within 3 months (26th Nov 2023). Please discuss the revision progress ahead of this time with the editor if you require more time to complete the revisions. Use the link below to submit your revision:

https://emboj.msubmit.net/cgi-bin/main.plex

Referee #1:

The convincing work by the Dutzler lab shows how a gating helix allosterically regulates Ca2+-dependent opening of the TMEM16A channel. By mutagenesis of the α6 gate they show that it effects the agonist-coupled rearrangements, by allosterically effecting channel opening by lowering the open state probability, and favouring a pre-opening state instead. This interpretation is consistent with an Cryo EM structure of the α6 gate mutant, which shows a conformational difference that supports the α-to-π-helix transition model for pore opening. Overall, they show that the gating helix sensitivity to Ca2+ -induced-rearrangements is directly linked to channel opening, which is an important contribution to the activation mechanism of TMEM16 channels.

Minor comments:

1. The α6 helix is a TM segment that sits within the membrane bilayer. The intrinsic dynamics of α6 helix should also contribute to its allosteric regulation. TMEM16A is an epithelial channel and HEK cells are adherent epithelial cells, and so presumably the lipid composition should be compatible? Based on electrophysiology recordings are IV curves similar to TMTM16A recordings in more native tissues? Essentially how much could the lipid bilayer composition influence this helix and its sensitivity to Ca2+?

2. In the electrophysiology measurements can Ca2+ be added to the inside directly?

3. From what I understand C2 symmetry was applied for 3D reconstruction. I am interested in the 3DVA of the WT vs mutant as I think it would be helpful for comparing the conformational differences. That is, that the mutants shift the conformational equilibrium, rather than a single reconstruction shown that could potentially be biased towards election of 3D classes.

Referee #2:

In this report by Lam and Dutzler, the authors employ electrophysiological and structural approaches to evaluate the role between a hydrophobic lock formed by Leu647 and Ile733 in channel gating. Together, these analyses nicely reveal how destabilization of this hydrophobic lock limits adoption of the fully activated state by disrupting the coupling between the Ca2+-binding site and the gate. While the data are strong, their presentation is confusing in places and textual revisions could significantly improve the manuscript for a general audience. With an improved presentation, the manuscript would be suitable for publication in EMBO Journal.

1. A rationale for evaluating the role of Leu647 and Ile733 would be helpful in guiding the reader as these mutations are a major thrust of this report. The authors introduce the mutations in the first paragraph of the results by stating "We now show that, amongst these residues, a hydrophobic interaction at the binding site and within the π-helical region, mediated by Leu 647 on α6 and Ile 733 on α8, is crucial in stabilizing this agonist-coupled rearrangement." This is the major conclusion of the manuscript and should be stating at the end, once the data supporting this conclusion. An alternative introduction that provides the reader

with rationale for why these residues were evaluated in a detailed manner should be included.
2. It is not clear from the structures presented in Figure 1A if Leu647 and Ile733 are in direct contact in the presented structure, nor in any of the alternative structures that have been described. Further comments on their interaction would also clarify their proposed roles in channel gating.
3. Do higher Ca concentrations enable the mutants to achieve the same MaxPo as WT? In the supplement WT appears to achieve max Po ~10-6. For the mutants, it is not clear that the mutants have achieved saturated activity in the evaluated conditions. Additional measurements at higher Ca2+ concentrations would be helpful in evaluating if they they do have a lower max Po or if they can achieve the same max Po as WT, but need higher Ca2+ concentrations to do so.
4. It is unclear why previous data is presented in Figure 1, rather than the new data presented in the supplement.
5. The meaning of truncations in the sentence "By analyzing different truncations, we further observed that the severity of the gating defects correlates with the hydrophobic volume mediating this interaction, as exemplified in the trends of the potency (EC50), cooperativity (nH), and Pomax changes" is lost on this reviewer. Please clarify this sentence.
6. The effect of the I773V mutation on the IC50 of 1PBC is very small (~20%) and potentially within the error of the measurements. It also seems to be consistent with across the measured voltage range. Why then is there a change in the position of the ligand with respect the electric field? Shouldn't a change in the depth of the ligand in the field result in a voltage-dependent shift in the IC50 as ligands? If not, then the calculations performed here, and their interpretation should be clarified.
7. It might be easier for the reader to follow if the cryo-EM analysis were presented earlier in the manuscript, potentially following Figure 1, as the structure would provide a framework for discussing the altered features of the mutant channel, such as the narrowed inner gate that are evaluated in Figures 2 and 3.
8. A direct comparison of the pore dimensions of the mutant with previously reported structures would further improve the presentation of the structure.
9. Noting the moderate and somewhat anisotropic resolution (see next point) of the presented L647V/I733V reconstruction, it would be helpful to present the densities corresponding to the ion binding site in the main figure. It is nice to have them in the supplement, but the raw densities are critical to evaluating the occupancy of the sites.
10. I would disagree that the reconstruction is isotropic. The 3DFSC plot indicates that the resolution in the Z-direction is considerably poorer (>1/2.5Å) than the reported overall resolution. The authors attempted to remedy the anisotropy by collecting data from tilted grids, however, most of these particles were discarded only the tilted data only contributed 4.3% of the final particles. (Note, the particle numbers in Table 1 and Figure S5 are different). While the anisotropy may not influence the interpretation of the map, it does appear to be present and should be described properly in the text.
11. How similar are the L647V/I733V and I551A structures? Both are described as partially activated but there are no direct comparisons.

Minor
1. "Shortening these residues to valine or mutating them to alanine severely impairs channel activation, as indicated by a pronounced right-shift in the potency of Ca2+" Both of these perturbations are mutations. Please simplify this comment.
2. The models should be labeled in Figure 4D-F.

We thank the reviewers for their constructive comments, and have incorporated the following changes in our manuscript.

Referee #1:

The convincing work by the Dutzler lab shows how a gating helix allosterically regulates Ca2+-dependent opening of the TMEM16A channel. By mutagenesis of the α6 gate they show that it effects the agonist-coupled rearrangements, by allosterically effecting channel opening by lowering the open state probability, and favouring a pre-opening state instead. This interpretation is consistent with an Cryo EM structure of the α6 gate mutant, which shows a conformational difference that supports the α-to-π-helix transition model for pore opening. Overall, they show that the gating helix sensitivity to Ca2+ -induced-rearrangements is directly linked to channel opening, which is an important contribution to the activation mechanism of TMEM16 channels.

Minor comments:

1. The α6 helix is a TM segment that sits within the membrane bilayer. The intrinsic dynamics of α6 helix should also contribute to its allosteric regulation. TMEM16A is an epithelial channel and HEK cells are adherent epithelial cells, and so presumably the lipid composition should be compatible? Based on electrophysiology recordings are IV curves similar to TMTM16A recordings in more native tissues? Essentially how much could the lipid bilayer composition influence this helix and its sensitivity to Ca2+?

The IV curves of TMEM16A expressed in HEK cells are similar to those obtained in more native tissues, for instance in pulmonary arterial smooth muscle cells, where a pseudo-linear instantaneous IV curve is observed in symmetrical chloride and where the activation time constants in response to voltage steps are in the same order of magnitude (Manoury et al 2010, J Physiol). Currently, we do not know if α6 might bind lipids directly but it is possible that the dynamics of this helix would be sensitive to the lipid bilayer composition and that the equilibrium between the resting and activated conformations might also be influenced by the bilayer composition, which could in turn affect the sensitivity to Ca^{2+}.

2. In the electrophysiology measurements can Ca2+ be added to the inside directly?

Yes, it can be. We recorded in the inside-out configuration, which exposes the intracellular side of the protein, and Ca^{2+} was perfused directly to the inside.

3. From what I understand C2 symmetry was applied for 3D reconstruction. I am interested in the 3DVA of the WT vs mutant as I think it would be helpful for comparing the conformational differences. That is, that the mutants shift the conformational equilibrium, rather than a single reconstruction shown that could potentially be biased towards election of 3D classes.

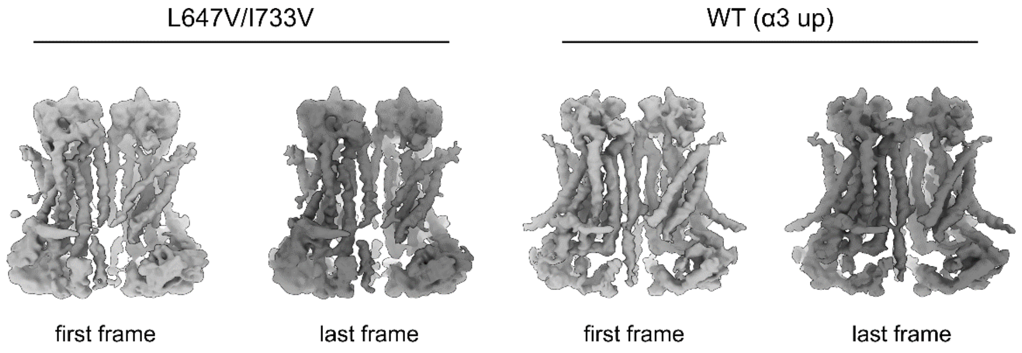

Fig. r1. 3DVA of L647V/I733V and WT. The two end frames are shown.

We performed 3DVA in C1 on the refined particles for both WT and the mutant and did not observe densities that might indicate the sampling of two discrete conformations of α6 (Fig. r1). As shown in Fig EV2, we performed 3D classification without any symmetry applied and did not observe classes that exhibit deviations from the final reconstruction. We also performed 3D refinement without symmetry applied and the resulting map superposes very well with the reconstruction obtained after imposing C2 symmetry. We note that the α6 conformation in the mutant has an additional off-plane component relative to the plane defined by the resting and activated states of α6, which supports a distinct conformation (see Fig. EV5).

Referee #2:

In this report by Lam and Dutzler, the authors employ electrophysiological and structural approaches to evaluate the role between a hydrophobic lock formed by Leu647 and Ile733 in channel gating. Together, these analyses nicely reveal how destabilization of this hydrophobic lock limits adoption of the fully activated state by disrupting the coupling between the Ca2+-binding site and the gate. While the data are strong, their presentation is confusing in places and textual revisions could significantly improve the manuscript for a general audience. With an improved presentation, the manuscript would be suitable for publication in EMBO Journal.

We have tried to improve the presentation of our manuscript and introduced changes in the text throughout.

1. A rationale for evaluating the role of Leu647 and Ile733 would be helpful in guiding the reader as these mutations are a major thrust of this report. The authors introduce the mutations in the first paragraph of the results by stating "We now show that, amongst these residues, a hydrophobic interaction at the binding site and within the π-helical region, mediated by Leu 647 on α6 and Ile 733 on α8, is crucial in stabilizing this agonist-coupled rearrangement." This is the major conclusion of the manuscript and should be stating at the end, once the data supporting this conclusion. An alternative introduction that provides the reader with rationale for why these residues were evaluated in a detailed manner should be included.

We have provided an alternative introduction with a rationale for why these residues were evaluated,

Line 101-105:

'In addition to this ionic interaction, our previous study has identified several residues around the Ca^{2+} binding site as important determinants of channel activation, which is manifested by a profound decrease in the potency of Ca^{2+} upon mutation to alanine (Lam *et al.*, 2021). Here, we focus on Leu 647 on α6 and Ile 733 on α8 within the π-helical region, which are brought into van der Waals contact in the Ca^{2+}-bound state upon α6 activation (Fig 1 and Fig EV1).'

and have moved the conclusion to the end of the paragraph.

Line 122-126:

'Together, these results suggest a hydrophobic interaction within the π-helical region close to the Ca^{2+} binding site, which is mediated by Leu 647 on α6 and Ile 733 on α8, to be crucial in stabilizing the agonist-coupled rearrangement of α6. The impairment of this interaction has evidently structural consequences that impact on the efficacy of pore opening, the agonist binding process, and channel conductance in TMEM16A.'

2. It is not clear from the structures presented in Figure 1A if Leu647 and Ile733 are in direct contact in the presented structure, nor in any of the alternative structures that have been described. Further comments on their interaction would also clarify their proposed roles in channel gating.

The distance between the closest contacts of Leu647 and Ile733 is approximately 4.5 Å in the Ca^{2+}-bound state (PDB: 7ZK3), which is within the range of a van der Waals interaction. We now show the sidechains of both residues with their molecular surface superimposed and provide a closeup view of the interaction region in the same panel (Fig 1A), which better illustrate the contact between the two residues.

We have also clarified in line 104-105:

'… Leu 647 on α6 and Ile 733 on α8 within the π-helical region, which are brought into van der Waals contact in the Ca^{2+}-bound state upon α6 activation (Fig 1 and Fig EV1).'

3. Do higher Ca concentrations enable the mutants to achieve the same MaxPo as WT? In the supplement WT appears to achieve max Po ~10-6. For the mutants, it is not clear that the mutants have achieved saturated activity in the evaluated conditions. Additional measurements at higher Ca2+ concentrations would be helpful in evaluating if they they do have a lower max Po or if they can achieve the same max Po as WT, but need higher Ca2+ concentrations to do so.

The maximum Po at the plateau of the concentration-response relations from Fig 1B for WT, L647V/I733V, and L647A/I733A are 0.78 ± 0.02, 0.66 ± 0.01, and 0.66 ± 0.005 (best-fit ± 95% CI) respectively. The Po data shown in Fig. 1C and D are practically identical to these values, indicating that, at the Ca^{2+} concentration where the Po was determined (15 and 50 µM Ca^{2+} for L647V/I733V and L647A/I733A respectively), all the constructs have achieved saturated activity. Therefore, we conclude from the data that the mutants cannot achieve the same maximum Po as WT even at a higher Ca^{2+} concentration because the activity is already saturated. It should also be noted that TMEM16A exhibits an additional activation phase that is not related to the gating process triggered through the principal Ca^{2+} binding site (Lim,

Lam, and Dutzler 2016 JGP) that we studied here. Thus, we typically avoid determining the Po at unnecessarily high Ca^{2+} concentrations, which causes faster current rundown that prevents an accurate determination of the noise parabolas, and we scale the concentration-response relations accordingly in order to estimate rigorously the Po at plateau, as shown in Fig 1B-D.

We have clarified in line 110-114:

'While the potency is influenced by both Ca^{2+} binding and channel gating, non-stationary-noise analysis indicates that this impairment in activation is due in part to a reduction in the channel's maximum open probability (Po_{max}) measured at a saturating ligand concentration, suggesting that Ca^{2+} has become less efficacious in these mutants (Fig 1D and E, Fig EV1C and E, and Appendix Table S2)'

And in line 522-524:

'Data are averages of 20 and 10 patches for L647V/I733V and L647A/I733A obtained at 15 µM and 50 µM Ca^{2+} respectively.'

4. It is unclear why previous data is presented in Figure 1, rather than the new data presented in the supplement.

We have replaced the data in Fig 1 with the new data.

5. The meaning of truncations in the sentence "By analyzing different truncations, we further observed that the severity of the gating defects correlates with the hydrophobic volume mediating this interaction, as exemplified in the trends of the potency (EC50), cooperativity (nH), and Pomax changes" is lost on this reviewer. Please clarify this sentence.

We have clarified in line 117-118:

'By analyzing different substituted aliphatic sidechains, we further observed that the severity of the gating defects correlates with…'

6. The effect of the I773V mutation on the IC50 of 1PBC is very small (~20%) and potentially within the error of the measurements. It also seems to be consistent with across the measured voltage range. Why then is there a change in the position of the ligand with respect the electric field? Shouldn't a change in the depth of the ligand in the field result in a voltage-dependent shift in the IC50 as ligands? If not, then the calculations performed here, and their interpretation should be clarified.

We analyzed the IC50 and zblock in the group of L647V, I733V, and L647V/I733V, and, while the effect of I733V is small, the main conclusion we make is that there exists a correlation between the degree of obstruction (conductance and rectification) and the depth of the 1PBC binding site (zblock) (Fig 3F), consistent with a narrowing of the pore being the origin of both phenomena that we summarized in Fig 3G. The degree of these defects depends on the severity of the mutations, which is small in I733V but is more profound in L647V/I733V. We have clarified in the following.

Line 211-214:

'…impaired α6 rearrangement is associated with putative structural changes at the outer entrance of the narrow neck, as suggested by the lower affinity of 1PBC in L647V/I733V and the related mutants, with varying degrees of severity (Fig 3E and F and Appendix Fig S3D-F).'

Line 217-218:

'In this group of mutants, the lowering of z_{block} correlates strongly with the elevation of the energy barrier inside the narrow neck…'

7. It might be easier for the reader to follow if the cryo-EM analysis were presented earlier in the manuscript, potentially following Figure 1, as the structure would provide a framework for discussing the altered features of the mutant channel, such as the narrowed inner gate that are evaluated in Figures 2 and 3.

We thank the reviewer for this suggestion. While we consider our structure to provide important and conclusive insight into the described mechanism, it is not in the center of the study. We thus decided to keep the current order to have a more thorough characterization of the functional defects in the mutants before providing a structural basis for the observed properties. To better reflect this focus, we have changed the title to 'Mechanistic basis of ligand efficacy in the calcium-activated chloride channel TMEM16A'.

8. A direct comparison of the pore dimensions of the mutant with previously reported structures would further improve the presentation of the structure.

We have included a direct comparison of the pore dimensions in Fig EV3 and have referenced in the following.

Line 240-243:

'The incomplete rearrangement of α6 is accompanied by a partial collapse of the extracellular part of the pore-lining helix α4 towards α6 (Fig 4C, left and Fig EV3), which is evident when compared with the 1PBC-bound, outer-pore-open conformation (Fig 4C, right and Fig EV3).'

9. Noting the moderate and somewhat anisotropic resolution (see next point) of the presented L647V/I733V reconstruction, it would be helpful to present the densities corresponding to the ion binding site in the main figure. It is nice to have them in the supplement, but the raw densities are critical to evaluating the occupancy of the sites.

We have added the density for the Ca^{2+} ions in the main figure, see Fig 4E. The density of the entire region is shown in Fig. EV2H.

10. I would disagree that the reconstruction is isotropic. The 3DFSC plot indicates that the resolution in the Z-direction is considerably poorer (>1/2.5Å) than the reported overall resolution. The authors attempted to remedy the anisotropy by collecting data from tilted grids, however, most of these particles were discarded only the tilted data only contributed

4.3% of the final particles. (Note, the particle numbers in Table 1 and Figure S5 are different). While the anisotropy may not influence the interpretation of the map, it does appear to be present and should be described properly in the text.

We agree that anisotropy is present and have acknowledged this in the text. Although the FSC curve in the Z-direction is of lower resolution, the density is of high quality and its distortion is comparably not pronounced (see Fig EV2 G and H). Although the number of particles contributed by the tilted dataset is small, they have contributed to the overall quality of the map. The numbers in Fig EV2 correspond to the particle images used in 3D classification, whereas those in Table 1 refer to the particles that contributed to the final reconstruction. We have made the following change.

Line 228-232:

'The reconstruction was obtained by combining datasets collected from samples prepared on cryo-EM grids with distinct chemical properties, each showing a distinct preferred orientation of particles, and at different tilts. Together, the pooled datasets yielded complementary particle views for the final map which, despite the remaining anisotropy, was much improved and provided a largely undistorted view of the protein (Table 1 and Fig EV2).'

11. How similar are the L647V/I733V and I551A structures? Both are described as partially activated but there are no direct comparisons.

We have included a superposition of the L647V/I733V and I551A structures in Fig EV5A and have referenced in the following.

Line 332-336:

'The correspondence between incomplete binding site closure and impeded channel opening in L647V/I733V, where α6 rearrangement is impaired, and the previously described apo I551A (Lam *et al.*, 2021), where the disruption of the gate results in a partial activation of α6 even in the absence of Ca^{2+} (Fig EV5A), suggests a general mechanism that governs agonist efficacy in TMEM16A.'

Minor

1. "Shortening these residues to valine or mutating them to alanine severely impairs channel activation, as indicated by a pronounced right-shift in the potency of Ca2+" Both of these perturbations are mutations. Please simplify this comment.

We have changed in line 106-108:

'Decreasing the sidechain volume of these residues by mutation to valine or alanine severely impairs channel activation, as indicated by a pronounced right-shift in the potency of Ca^{2+} (Fig 1B and C and Appendix Table S1).'

2. The models should be labeled in Figure 4D-F.

We have labelled the models in Fig 4D-F.

1st Revision - Editorial Decision **24th Oct 2023**

Dear Raimund,

Thank you for submitting a revised version of your manuscript. It was sent to both of the original referees; I have now heard back from one of them. This brief statement is attached to the bottom of this email. As you will see, you have addressed all of the original concerns adequately; therefore, barring any unexpected and critical interventions from either Referee 1, I am happy to proceed towards publication with your manuscript. There are some remaining editorial issues which need to be addressed. In this regard, would you please:

include up to five keywords,
remove the author credit section,
rename the dataset file 'EV1' with the corresponding callout in the text; we were unable to play the video zipped in the Dataset file,
as no SD files were uploaded with the revision (although there is a completed SD checklist), please confirm whether the SD files have been deposited in external repositories,
provide the accession IDs and URLs for the 'PDB and EMDB' datasets in the data availability statement,
define 'n' in the legend of figures 1c, e, f; 2c, d; 3d, f; 5c, e, f, h; and EV1d-e, and
place main and EV Figure legends after the References

Additionally, could you please supply the synopsis image in jpeg, TIFF or png format, and remove the bullet points for the summary form the main file. We include a two sentence synopsis of the paper (see http://emboj.embopress.org/). Please provide me with this summary along with 3-5 bullet points that capture the key findings of the paper.

I look forward to receiving these changes. EMBO Press is an editorially independent publishing platform for the development of EMBO scientific publications.

Best wishes,

Will

William Teale, PhD
Editor
The EMBO Journal
w.teale@embojournal.org

Instructions for preparing your revised manuscript:

Please check that the title and abstract of the manuscript are brief, yet explicit, even to non-specialists.

When assembling figures, please refer to our figure preparation guideline in order to ensure proper formatting and readability in print as well as on screen:
https://bit.ly/EMBOPressFigurePreparationGuideline
See also figure legend guidelines: https://www.embopress.org/page/journal/14602075/authorguide#figureformat

IMPORTANT: When you send the revision we will require
- a point-by-point response to the referees' comments, with a detailed description of the changes made (as a word file).
- a word file of the manuscript text.
- individual production quality figure files (one file per figure)
- a complete author checklist, which you can download from our author guidelines (https://www.embopress.org/page/journal/14602075/authorguide).
- Expanded View files (replacing Supplementary Information)
Please see out instructions to authors
https://www.embopress.org/page/journal/14602075/authorguide#expandedview

Please remember: Digital image enhancement is acceptable practice, as long as it accurately represents the original data and conforms to community standards. If a figure has been subjected to significant electronic manipulation, this must be noted in the figure legend or in the 'Materials and Methods' section. The editors reserve the right to request original versions of figures and the original images that were used to assemble the figure.

Further information is available in our Guide For Authors: https://www.embopress.org/page/journal/14602075/authorguide

We realize that it is difficult to revise to a specific deadline. In the interest of protecting the conceptual advance provided by the work, we recommend a revision within 3 months (22nd Jan 2024). Please discuss the revision progress ahead of this time with the editor if you require more time to complete the revisions. Use the link below to submit your revision:

https://emboj.msubmit.net/cgi-bin/main.plex

Referee #2:

The authors have addressed all issues and the manuscript is suitable for publication in the EMBO Journal.

All editorial and formatting issues were resolved by the authors.

2nd Revision - Editorial Decision 2nd Nov 2023

Dear Raimund,

I am pleased to inform you that your manuscript has been accepted for publication in the EMBO Journal.

Congratulations to you both on a really insightful study!

Please note that it is EMBO Journal policy for the transcript of the editorial process (containing referee reports and your response letter) to be published as an online supplement to each paper. If you do NOT want this, you will need to inform the Editorial Office via email immediately. More information is available here: https://www.embopress.org/page/journal/14602075/authorguide#transparentprocess

Your manuscript will be processed for publication in the journal by EMBO Press. Manuscripts in the PDF and electronic editions of The EMBO Journal will be copy edited, and you will be provided with page proofs prior to publication. Please note that supplementary information is not included in the proofs.

You will be contacted by Wiley Author Services to complete licensing and payment information. The required 'Page Charges Authorization Form' is available here: https://www.embopress.org/pb-assets/embo-site/tej_apc.pdf - please download and complete the form and return to embopressproduction@wiley.com

EMBO Press participates in many Publish and Read agreements that allow authors to publish Open Access with reduced/no publication charges. Check your eligibility: https://authorservices.wiley.com/author-resources/Journal-Authors/open-access/affiliation-policies-payments/index.html

Should you be planning a Press Release on your article, please get in contact with embojournal@wiley.com as early as possible, in order to coordinate publication and release dates.

If you have any questions, please do not hesitate to call or email the Editorial Office. Thank you for your contribution to The EMBO Journal.

Yours sincerely,

William

William Teale, PhD
Editor
The EMBO Journal
w.teale@embojournal.org

** Click here to be directed to your login page: https://emboj.msubmit.net

EMBO Press Author Checklist

Corresponding Author Name: Andy K. M. Lam, Raimund Dutzler
Journal Submitted to: The EMBO Journal
Manuscript Number: EMBOJ-2023-115030

USEFUL LINKS FOR COMPLETING THIS FORM
The EMBO Journal - Author Guidelines
EMBO Reports - Author Guidelines
Molecular Systems Biology - Author Guidelines
EMBO Molecular Medicine - Author Guidelines

Reporting Checklist for Life Science Articles (updated January
This checklist is adapted from Materials Design Analysis Reporting (MDAR) Checklist for Authors. MDAR establishes a minimum set of requirements in transparent reporting in the life sciences (see Statement of Task: 10.31222/osf.io/9sm4x). Please follow the journal's guidelines in preparing your
Please note that a copy of this checklist will be published alongside your article.

Abridged guidelines for figures
1. Data
The data shown in figures should satisfy the following conditions:
- the data were obtained and processed according to the field's best practice and are presented to reflect the results of the experiments in an accurate and unbiased manner.
- ideally, figure panels should include only measurements that are directly comparable to each other and obtained with the same assay.
- plots include clearly labeled error bars for independent experiments and sample sizes. Unless justified, error bars should not be shown for technical
- if n<5, the individual data points from each experiment should be plotted. Any statistical test employed should be justified.
- Source Data should be included to report the data underlying figures according to the guidelines set out in the authorship guidelines on Data

2. Captions
Each figure caption should contain the following information, for each panel where they are relevant:
- a specification of the experimental system investigated (eg cell line, species name).
- the assay(s) and method(s) used to carry out the reported observations and measurements.
- an explicit mention of the biological and chemical entity(ies) that are being measured.
- an explicit mention of the biological and chemical entity(ies) that are altered/varied/perturbed in a controlled manner.
- the exact sample size (n) for each experimental group/condition, given as a number, not a range;
- a description of the sample collection allowing the reader to understand whether the samples represent technical or biological replicates (including how many animals, litters, cultures, etc.).
- a statement of how many times the experiment shown was independently replicated in the laboratory.
- definitions of statistical methods and measures:
  - common tests, such as t-test (please specify whether paired vs. unpaired), simple χ2 tests, Wilcoxon and Mann-Whitney tests, can be unambiguously identified by name only, but more complex techniques should be described in the methods section;
  - are tests one-sided or two-sided?
  - are there adjustments for multiple comparisons?
  - exact statistical test results, e.g., P values = x but not P values < x;
  - definition of 'center values' as median or average;
  - definition of error bars as s.d. or s.e.m.

Please complete ALL of the questions below.
Select "Not Applicable" only when the requested information is not relevant for your study.

Materials

**Newly Created Materials**	**Information included in the manuscript?**	**In which section is the information available?** (Reagents and Tools Table, Materials and Methods, Figures, Data Availability Section)
New materials and reagents need to be available; do any restrictions apply?	Not Applicable	

**Antibodies**	**Information included in the manuscript?**	**In which section is the information available?** (Reagents and Tools Table, Materials and Methods, Figures, Data Availability Section)
For **antibodies** provide the following information:<br>- Commercial antibodies: RRID (if possible) or supplier name, catalogue number and or/clone number<br>- Non-commercial: RRID or citation	Not Applicable	

**DNA and RNA sequences**	**Information included in the manuscript?**	**In which section is the information available?** (Reagents and Tools Table, Materials and Methods, Figures, Data Availability Section)
**Short novel DNA or RNA including primers, probes**: provide the sequences.	Yes	Materials and Methods

**Cell materials**	**Information included in the manuscript?**	**In which section is the information available?** (Reagents and Tools Table, Materials and Methods, Figures, Data Availability Section)
**Cell lines:** Provide species information, strain. Provide accession number in repository **OR** supplier name, catalog number, clone number, and/**OR** RRID.	Yes	Materials and Methods
**Primary cultures:** Provide species, strain, sex of origin, genetic modification status.	Not Applicable	
Report if the cell lines were recently **authenticated** (e.g., by STR profiling) and tested for mycoplasma contamination.	Yes	Materials and Methods

**Experimental animals**	**Information included in the manuscript?**	**In which section is the information available?** (Reagents and Tools Table, Materials and Methods, Figures, Data Availability Section)
**Laboratory animals or Model organisms:** Provide species, strain, sex, age, genetic modification status. Provide accession number in repository **OR** supplier name, catalog number, clone number, **OR** RRID.	Not Applicable	
**Animal observed in or captured from the field:** Provide species, sex, and age where possible**.**	Not Applicable	
Please detail **housing and husbandry conditions**.	Not Applicable	

**Plants and microbes**	**Information included in the manuscript?**	**In which section is the information available?** (Reagents and Tools Table, Materials and Methods, Figures, Data Availability Section)
**Plants:** provide species and strain, ecotype and cultivar where relevant, unique accession number if available, and source (including location for collected wild specimens).	Not Applicable	
**Microbes:** provide species and strain, unique accession number if available, and source.	Not Applicable	

**Human research participants**	**Information included in the manuscript?**	**In which section is the information available?** (Reagents and Tools Table, Materials and Methods, Figures, Data Availability Section)
If collected and within the bounds of privacy constraints report on age, sex and gender or ethnicity for all study participants.	Not Applicable	

**Core facilities**	**Information included in the manuscript?**	**In which section is the information available?** (Reagents and Tools Table, Materials and Methods, Figures, Data Availability Section)
If your work benefited from core facilities, was their service mentioned in the acknowledgments section?	Yes	Acknowledgement

Design

Study protocol	Information included in the manuscript?	In which section is the information available? (Reagents and Tools Table, Materials and Methods, Figures, Data Availability Section)
If study protocol has been **pre-registered, provide DOI in the manuscript**. For clinical trials, provide the trial registration number **OR** cite DOI.	Not Applicable	
Report the **clinical trial registration number** (at ClinicalTrials.gov or equivalent), where applicable.	Not Applicable	

Laboratory protocol	Information included in the manuscript?	In which section is the information available? (Reagents and Tools Table, Materials and Methods, Figures, Data Availability Section)
Provide DOI OR other citation details if **external detailed step-by-step protocols** are available.	Not Applicable	

Experimental study design and statistics	Information included in the manuscript?	In which section is the information available? (Reagents and Tools Table, Materials and Methods, Figures, Data Availability Section)
Include a statement about **sample size** estimate even if no statistical methods were used.	Yes	Materials and Methods
Were any steps taken to minimize the effects of subjective bias when allocating animals/samples to treatment (e.g. **randomization procedure**)? If yes, have they been described?	Not Applicable	
Include a statement about **blinding** even if no blinding was done.	Yes	Materials and Methods
Describe **inclusion/exclusion criteria** if samples or animals were excluded from the analysis. Were the criteria pre-established?<br><br>If sample or data points were omitted from analysis, report if this was due to attrition or intentional exclusion and provide justification.	Not Applicable	
For every figure, are **statistical tests** justified as appropriate? Do the data meet the assumptions of the tests (e.g., normal distribution)? Describe any methods used to assess it. Is there an estimate of variation within each group of data? Is the variance similar between the groups that are being statistically compared?	Yes	Materials and Methods

Sample definition and in-laboratory replication	Information included in the manuscript?	In which section is the information available? (Reagents and Tools Table, Materials and Methods, Figures, Data Availability Section)
In the figure legends: state number of times the experiment was **replicated** in laboratory.	Yes	Figure legends
In the figure legends: define whether data describe **technical or biological replicates**.	Yes	Figure legends

Ethics

Ethics	Information included in the manuscript?	In which section is the information available? (Reagents and Tools Table, Materials and Methods, Figures, Data Availability Section)
Studies involving **human participants**: State details of **authority granting ethics approval** (IRB or equivalent committee(s), provide reference number for approval.	Not Applicable	
Studies involving **human participants**: Include a statement confirming that **informed consent** was obtained from all subjects and that the experiments conformed to the principles set out in the WMA Declaration of Helsinki and the Department of Health and Human Services Belmont Report.	Not Applicable	
Studies involving **human participants:** For publication of **patient photos**, include a statement confirming that consent to publish was obtained.	Not Applicable	
Studies involving experimental **animals**: State details of **authority granting ethics approval** (IRB or equivalent committee(s), provide reference number for approval. Include a statement of compliance with ethical regulations.	Not Applicable	
Studies involving **specimen and field samples:** State if relevant **permits** obtained, provide details of authority approving study; if none were required, explain why.	Not Applicable	

Dual Use Research of Concern (DURC)	Information included in the manuscript?	In which section is the information available? (Reagents and Tools Table, Materials and Methods, Figures, Data Availability Section)
Could your study fall under dual use research restrictions? Please check biosecurity documents and list of **select agents and toxins** (CDC): https://www.selectagents.gov/sat/list.htm	Not Applicable	
If you used a select agent, is the security level of the lab appropriate and reported in the manuscript?	Not Applicable	
If a study is subject to dual use research of concern regulations, is the name of the **authority granting approval and reference number** for the regulatory approval provided in the manuscript?	Not Applicable	

Reporting

The MDAR framework recommends adoption of discipline-specific guidelines, established and endorsed through community initiatives. Journals have their own policy about requiring specific guidelines and recommendations to complement MDAR.

Adherence to community standards	Information included in the manuscript?	In which section is the information available? (Reagents and Tools Table, Materials and Methods, Figures, Data Availability Section)
State if relevant guidelines or checklists (e.g., **ICMJE, MIBBI, ARRIVE, PRISMA**) have been followed or provided.	Not Applicable	
For **tumor marker prognostic studies,** we recommend that you follow the **REMARK** reporting guidelines (see link list at top right). See author guidelines, under 'Reporting Guidelines'. Please confirm you have followed these guidelines.	Not Applicable	
For **phase II and III randomized controlled trials**, please refer to the **CONSORT** flow diagram (see link list at top right) and submit the CONSORT checklist (see link list at top right) with your submission. See author guidelines, under 'Reporting Guidelines'. Please confirm you have submitted this list.	Not Applicable	

Data Availability

Data availability	Information included in the manuscript?	In which section is the information available? (Reagents and Tools Table, Materials and Methods, Figures, Data Availability Section)
Have **primary datasets** been deposited according to the journal's guidelines (see 'Data Deposition' section) and the respective accession numbers provided in the Data Availability Section?	Yes	Data Availability
Were **human clinical and genomic datasets** deposited in a public access-controlled repository in accordance to ethical obligations to the patients and to the applicable consent agreement?	Not Applicable	
Are **computational models** that are central and integral to a study available without restrictions in a machine-readable form? Were the relevant accession numbers or links provided?	Not Applicable	
If publicly available data were reused, provide the respective **data citations in the reference list**.	Not Applicable	
